# A long-lasting prolactin stimulates galactopoiesis in mice

**DOI:** 10.1016/j.isci.2025.113112

**Published:** 2025-07-15

**Authors:** Kasia Kready, Kailyn E. Doiron, Katherine Redfield Chan, Jeffrey C. Way, Quincey Justman, Camille E. Powe, Pamela A. Silver

**Affiliations:** 1 Department of Systems Biology, Harvard Medical School, Boston, MA 02115, USA; 2 Wyss Institute for Biologically Inspired Engineering, Harvard University, Boston, MA 02115, USA; 3 Synthetic Biology Hive, Harvard Medical School, Boston, MA 02115, USA; 4 Department of Anesthesiology, Pain and Perioperative Medicine, Brigham and Women’s Hospital, Boston, MA 02115, USA; 5 Diabetes Unit, Division of Endocrinology, Massachusetts General Hospital, Boston, MA 02114, USA; 6 Departments of Medicine and of Obstetrics, Gynecology, and Reproductive Biology, Harvard Medical School, Boston, MA 02115, USA; 7 Broad Institute, Cambridge, MA 02142, USA

**Keywords:** Biochemistry, Physiology, Biochemical engineering, Pharmaceutical Engineering

## Abstract

Prolactin is the main hormonal driver of mammalian lactation. To sustain milk production, basal prolactin levels must remain elevated compared to nonpregnant states. However, prolactin (23 kDa) is short-lived in circulation due to rapid renal excretion. Here, we design and test the galactopoietic effects of an engineered long-lasting prolactin in mice. The engineered variant, prolactin-extra long-acting (Prolactin-XL), is comprised of endogenously active human prolactin fused to an engineered human immunoglobulin G1 (IgG1) Fc domain. Prolactin-XL has a serum half-life of 70.9 h in mice, 2,625-fold longer than endogenously active human prolactin alone (70.9 h vs. 0.02 h). Prolactin-XL is engineered to be more susceptible to gastrointestinal proteases to reduce its uptake by nursing neonates. We demonstrate that Prolactin-XL increases lactation and restores growth of pups fed by dams with pharmacologically ablated lactation. We propose that Prolactin-XL is a potential tool for the study and pharmacologic stimulation of galactopoiesis.

## Highlights

• Prolactin-XL was engineered to be breastfeeding-compatible
• Prolactin-XL has a 2,625-fold longer half-life than endogenously active prolactin in mice
• Prolactin-XL has low oral bioavailability to limit infant drug exposure
• Prolactin-XL restores lactation in a mouse model of pharmacologically ablated lactation

## Graphical abstract

**Figure:**
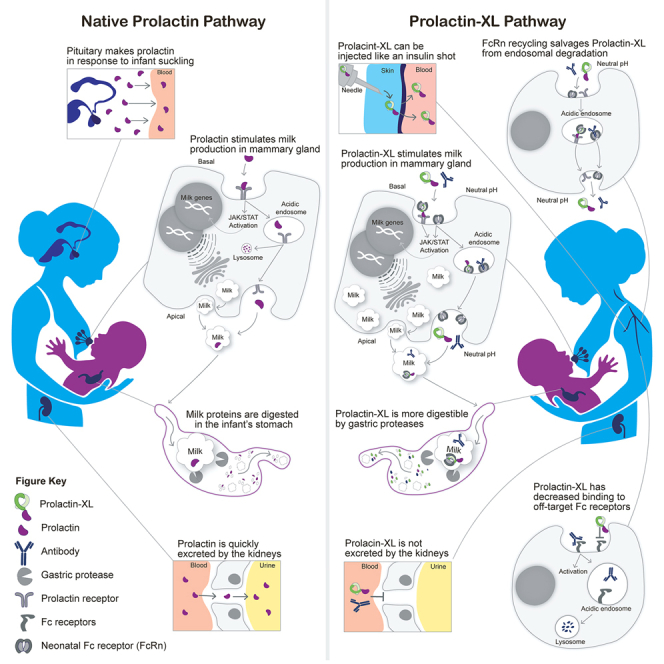

## Introduction

Prolactin is the key hormonal driver of galactopoiesis, the process of milk production. Galactopoiesis in all mammals is dependent on milk removal and suckling-induced prolactin secretion from the anterior pituitary. If a mammal is not nursing, experiences weak suckling-induced stimulation of the mammary gland, or has inadequate milk removal, then prolactin levels decrease, leading to weaning.^1^ In rare cases in humans, postpartum persons with very low or no prolactin have failed lactogenesis. This can be idiopathic or the result of a genetic mutation in the prolactin gene, postpartum pituitary necrosis caused by peripartum hemorrhage (Sheehan’s syndrome), or autoantibodies against lactotrophs, the prolactin-producing cells in the anterior pituitary.^1^^,^^2^^,^^3^^,^^4^^,^^5^ Other common risk factors for lactation insufficiency include insufficient glandular tissue, infertility, breast surgery, endocrine disorders, and obesity.^6^ For some with these risk factors and lactation insufficiency, it is likely that relative prolactin deficiency is the clinical end-result, driven by prolactin’s pharmacokinetics.^7^

Prolactin is primarily synthesized in the anterior pituitary and is largely eliminated passively by the kidneys due to its small size.^8^ It has a short serum half-life of ∼40 min in humans.^8^ Nursing maintains high basal prolactin levels (100–300 ng/mL) during galactopoiesis in humans.^1^ The biologically active form of human prolactin (PRL) is a monomeric 23 kDa protein that binds to prolactin receptor (PRLR) dimers expressed in milk-making alveolar cells of the mammary gland.^9^ When prolactin binds PRLR dimers, it induces pro-survival signaling via the JAK/STAT pathway, which is essential for maintaining milk production.^9^ In mice, milk-making cells must receive prolactin’s pro-survival signaling within 12–48 h, or a reversible phase of involution begins.^10^ If a lactating person, like other mammals, is not nursing or has inadequate nipple stimulation or milk removal, prolactin levels decrease causing relative prolactin deficiency, low milk supply, and premature weaning.^1^

Frequent exogenous prolactin administration can rescue lactation insufficiency in mice and other mammals.^11^ In the case of mice and other animal models, galactopoiesis is pharmacologically ablated with dopaminergic agonists that block prolactin secretion from the anterior pituitary gland.^11^ In a phase 2 clinical trial in humans, the recombinant form of human prolactin (r-PRL) was shown to increase milk supply in postpartum parents with prolactin deficiency and/or lactation insufficiency when given twice daily, with no adverse side effects to nursing parent or infant.^12^ However, R-PRL’s short serum half-life makes it an unfavorable pharmacologic candidate due to the need for frequent injections.

Recent successes demonstrate that a protein’s serum half-life can be increased by fusing it to the fragment crystallizable (Fc) region of the constant domain of human immunoglobulins (IgGs).^13^^,^^14^^,^^15^^,^^16^ We therefore engineered a long-acting, human Fc-prolactin fusion called Prolactin-XL. Prolactin-XL is comprised of endogenously active human prolactin fused to an engineered human IgG1 Fc domain. Its key features include: (1) an active, monomeric, non-glycosylated human prolactin mutant, (2) a 5-amino acid flexible linker, and (3) non-glycosylated, heterodimeric human Fc mutants with asymmetric binding to Protein A. Because lactating parents often forgo medications or stop breastfeeding due to concerns of infant drug exposure, Prolactin-XL is engineered for enhanced degradation in the gastric system to minimize neonatal systemic exposure. Here, we demonstrate that Prolactin-XL has a long serum half-life and promotes galactopoiesis in mice.

## Results

### Design of human Fc-prolactin fusion variants

To engineer a long-acting human Fc-prolactin fusion variant, our design challenge was 4-fold: (1) fuse an endogenously active human prolactin variant to an Fc domain without disrupting PRLR signaling, (2) decrease human Fc domain’s binding to off-target Fc receptors, (3) improve the serum half-life vs. prolactin, and (4) minimize uptake by the nursing neonates by decreasing oral bioavailability. To address these challenges, we used existing literature and structural information from solved structures of human prolactin, human PRLR, and homologous hormones and receptors in highly similar complexes (PDBs: 1f6f, 3d48, 1rw5) to rationally engineer and score 29 human Fc-prolactin fusion variants.^2^^,^^17^^,^^18^^,^^19^^,^^20^^,^^21^^,^^22^^,^^23^^,^^24^^,^^25^^,^^26^ In principle, the resulting fusions integrate the multiple functions and pharmacokinetics of both the Fc domain and prolactin to increase basal prolactin levels, without impeding the natural suckling-stimulated secretion of pituitary-derived prolactin. For more details, refer to Tables S1–S3 and S4 for more information on (1) functional descriptions of relevant human endogenous molecules impacting the pharmacokinetics of Fc-prolactin variants, (2) a list of mutations used in Fc-prolactin variants, (3) design schemes used in the Fc-prolactin variants, and (4) the specific Fc-prolactin fusion variants used in this study. A brief summary of Fc-prolactin variant design is below.

**Figure 1:**
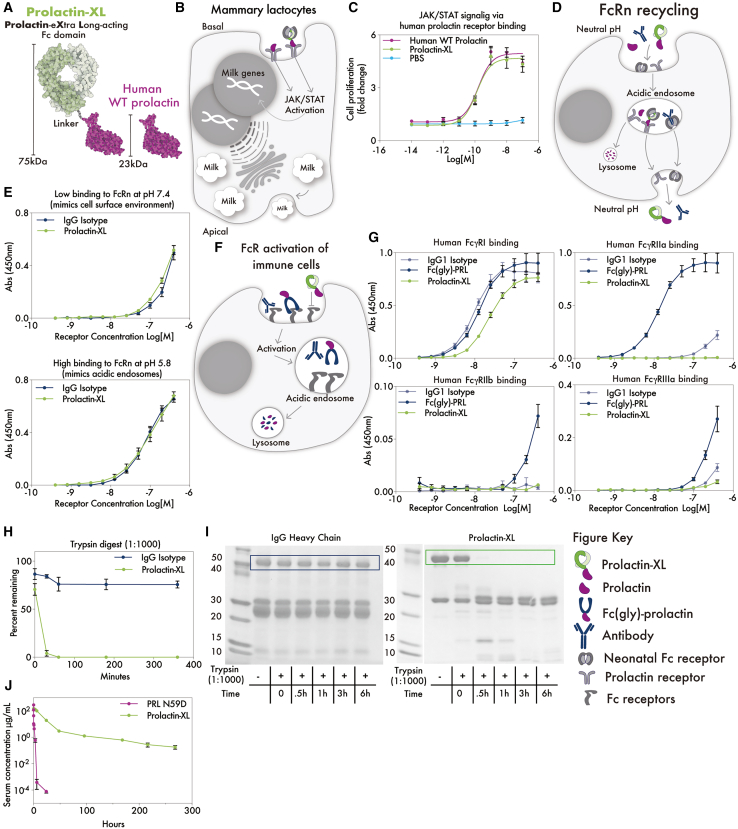
Prolactin-XL is the top-performing variant in *in vitro* screens and has the longest *in vivo* serum half-life (A) A cartoon of Prolactin-XL compared to human WT prolactin. (B) A cartoon of a Fc-prolactin fusion binding to prolactin receptor (PRLR) to activate the JAK/STAT proliferation on mammary alveolar cells. Human WT His-tagged Prolactin was used as a positive control. (C) A cell-based assay measuring Fc-prolactin fusions induction of *in vitro* signaling via human PRLR stably expressed in Ba/F3 cells. Data are represented as mean ± SEM of triplicates, and PRISM was used to fit non-linear curves. (D) A cartoon of FcRn-mediated recycling of Fc-prolactin fusions. (E) Binding of Fc-prolactin fusions to human FcRn:β2m at pH 7.4 or pH 5.8 measured by ELISA. Data are represented as mean ± SEM of triplicates. (F) A cartoon of Fc-prolactin fusions interacting with Fc receptors to activate immune effector cells. (G) Binding of Fc-prolactin fusions to human FcɣRI, human FcɣRIIa, human FcɣRIIb, or human FcɣRIIIa measured by ELISA. Data are represented as mean ± SEM of triplicates. (H and I) IgG isotype control or Prolactin-XL was digested by Trypsin at a ratio 1:1,000 (enzyme:protein). In (I), the IgG heavy chain or the Fc-Prolactin-XL monomer is outlined and was used for densitometry. The percent of the remaining fusion were analyzed by SDS-PAGE (I) and measured by densitometry (H). Data are represented as mean ± SEM of triplicates. (J) Nulliparous Tg276 mice were injected with 5 mg/kg i.v. of Prolactin-XL (*n* = 4) and PRL N59D (*n* = 5). Blood was collected by tail nick post injection, and the concentration of the fusions in serum was measured by ELISA. The data are depicted as mean ± SEM. PRISM was used to fit either a one-phase decay (PRL N59D) or a two-phase decay (Prolactin-XL).

The 29 engineered variants vary in domain topology, linker regions, and are composed of homo- and heterodimers to maximize the likelihood of active PRLR signaling (Figure 1B; Tables S1–S3 and S4).^20^^,^^21^^,^^22^ For example, prolactin exists in both active non-glycosylated and inactive glycosylated isoforms, where glycosylation sterically interferes with PRLR binding (Table S1).^27^ To address this, we removed the glycosylation site by identifying the naturally occurring, deglycosylated PRL N59D mutant from bovine, mouse, rat, water buffalo, and yak prolactin sequences (Tables S2 and S3). All variants contain this mutation, except Fc-PRL-2. Additionally, both termini of prolactin are key PRLR binding sites, so we designed N-prolactin-Fc-C and N-Fc-prolactin-C variants to determine the optimal termini for fusion (Tables S2 and S3). To prevent the Fc domain from clashing with PRLR, some variants were made with a flexible GGsGG linker. Since prolactin also exists in another naturally occurring inactive isoform, as dimers, we constructed Fc-prolactin heterodimers (Tables S2 and S3). These heterodimeric fusion variants consist of a Fc-prolactin monomer covalently linked via disulfide bonds to a Fc-only monomer, whereas homodimeric fusion variants consist of two Fc-prolactin monomers covalently linked via disulfide bonds.

The 29 engineered variants also vary in Fc mutations to prevent off-target binding to Fc receptors, enhance half-life via FcRn, and reduce oral bioavailability. Fc domains can bind to at least six Fc receptors variably expressible on immune cells, triggering downstream signaling and receptor-mediated endosomal degradation (Table S1).^28^ To reduce off-target Fc receptor binding, we incorporated well-characterized IgG1 Fc mutations in some variants (Tables S2 and S3).^29^^,^^30^^,^^31^ Fc domains can increase serum half-life via at least two mechanisms: by promoting endosomal recycling over lysosomal degradation at the cellular level and by raising the construct’s molecular weight over 70 kDa to prevent renal clearance at the organ level.^13^ Our engineered variants have molecular weights of at least 75 kDa, and some contain Fc domain mutations previously shown to enhance FcRn-mediated endosomal recycling, such as “YTE” or “EDHS” mutations.^24^ In parallel, to maximize half-life in maternal circulation, we sought to minimize infant exposure via breast milk transmission. To do so, we aimed to reduce oral bioavailability by removing the IgG1 F(ab) domain and Fc glycosylation site (Tables S2 and S3).^29^

### Identification of a long-acting human Fc-prolactin fusion variant, Prolactin-XL

We scored performance of all the 29 Fc-prolactin variants in *in vitro* screens and report the results in Figures S1–S8 and Tables S5–S7. Patterns emerging from the screening of all 29 engineered variants are reported in Table S5. Below is a brief summary of the *in vitro* screening process.

First, variants were transiently expressed in Hek293F cells, purified via His or Protein A purification, and assessed for expression and purity (Figures S1 and S2). All variants were successfully expressed, with yields ranging from ∼1 to 40 μg/L (Figure S2). Patterns emerged in yields across the variants and are discussed in Table S5. For example, homodimer variants generally had higher yields than heterodimer variants (compare Figure S3A; Fc-PRL-# 1–7 vs. Fc-PRL-# 8–29). Additionally, N-Fc-prolactin-C heterodimers showed higher yields than N-prolactin-Fc-C heterodimers (Figure S3A; compare Fc-PRL-# 8–17 vs. Fc-PRL-# 18–29). After expression and purity screening, all variants were tested for *in vitro* binding and signaling assays.

Engineered variants were next scored for their ability to signal via the human or mouse PRLR by measuring *in vitro* proliferation of murine Ba/F3 cells stably expressing PRLR (Figures S4 and S5; Tables S6 and S7). For human PRLR, the potency of the variants ranged from −6.9 M to −10.3 M, and the efficacy ranged from 49% to 127%. Patterns among the potency (Log(EC_50_)) and efficacy (% WT human prolactin Emax) of the 29 variants are discussed in Table S5. Notably, homodimers were found to be more potent and efficacious than heterodimers (Table S6; compare Fc-PRL-# 1–7 vs. Fc-PRL-# 8–29). Additionally, a GGsGG flexible linker decreased potency and efficacy in homodimers (Table S6; compare Fc-PRL-4 vs. Fc-PRL-# 1–3, 5–7), whereas it increased efficacy in heterodimers (Table S6; compare Fc-PRL-8 vs. Fc-PRL-# 9–29). After establishing that fusing Fc to prolactin leads to active PRLR binding and signaling, fusions were further evaluated for Fc-mediated interactions.

As such, variants were first scored for their ability to bind to human or mouse FcRn *in vitro* at neutral and acidic pH, to mimic cell surface and endosome conditions, respectively, via ELISA (Figure S6). All variants bound human FcRn in a pH-dependent manner (Figure S6). Notably, Fc-prolactin variants with YTE mutations showed enhanced pH-dependent human FcRn binding *in vitro* (Figures S7A and S7B; compare Fc-PRL-7, 17, and 29 vs. Fc-PRL-1, 3, and 13). Some heterodimeric variants had incorporated an RF mutation to abolish FcRn binding on the Fc-only monomer to improve purity.^23^ While RF mutations did not affect human FcRn binding *in vitro* (Figures S7A and S7B), they might abolish mouse FcRn binding *in vivo* (Figure S7C; compare Fc-PRL-1 and 7 vs. Fc-PRL-13). After confirming “on-target” FcRn binding, we further screened fusions for off-target FcR binding.

Variants were scored for reduced off-target binding to human and mouse FcRs *in vitro* via ELISA (Figure S7). The glycosylated Fc-prolactin variant (Fc-PRL-1) exhibited higher binding to human FcRs than WT IgG1 isotype controls, particularly outcompeting isotype controls for human FcɣRI binding (Figure S8). All variants, besides Fc-PRL-1, have a deglycosylated Fc domain. Deglycosylating Fc generally reduced human and mouse FcR binding except for human FcɣRI. But in the presence of competing IgG, deglycosylated Fc has drastically lower human FcɣRI binding than controls (Figures S8A vs S8B). After scoring the fusions for reduced FcR binding, we next evaluated variants for gastrointestinal and serum stability.

To reduce infant exposure while maintaining long-lasting persistence in maternal serum, we screened the variants for increased gastrointestinal (GI) protease cleavage. The Fc used in the fusions is a subdomain of IgG1, which has natively low oral bioavailability measured to be <25% in infants.^29^^,^^32^ Our variants were further engineered to be more digestible in the stomach by removing the F(ab) domain and de-glycosylating the Fc domain to enhance digestion by GI proteases.^29^ Engineered variants were scored for their *in vitro* susceptibility to digestion by the canonical GI proteases, trypsin, chymotrypsin, and pepsin via SDS-PAGE (Figures S8 and S9).^29^ For all three GI proteases, there was identifiable cleavage at the interface between Fc and prolactin, as well as internally within both molecules (Figures S8 and S9). To confirm these modifications did not affect serum proteolysis, we incubated Fc-PRl-13 with mouse serum *in vitro* and found no identifiable cleavage products by western blot (Figure S10). After scoring fusions for GI and serum stability, we completed the suite of *in vitro* screening.

Next we selected the top four performing variants across *in vitro* screens by ranking all variants and selecting the highest-ranking ones from across the screens. They were Fc-PRL-3, Fc-PRL-7, Fc-PRL-13, and Fc-PRL-17. These variants are all N-Fc-prolactin-C fusions. Fc-PRL-3 and Fc-PRL-7 are homodimer variants, and Fc-PRL-13 and Fc-PRL-17 are heterodimer variants. Fc-PRL-13 and Fc-PRL-17 have a flexible linker. Fc-PRL-7 and Fc-PRL-17 contain “YTE” mutations in the Fc domain. Theses fusions were then expressed in higher volumes to produce enough material for *in vivo* pharmacokinetic studies.

We then tested for long-lasting persistence *in vivo* by evaluating the top four performing variants in humanized mice. We performed pharmacokinetic studies in nulliparous Tg276 mice that have mouse FcRn knocked-out and are transgenic for human FcRn. Tg276 mice are commonly used by the drug development industry to study antibody pharmacokinetics because they are among the best preclinical predictors of pharmacokinetics in humans.^33^ Tg276 mice were administered a single dose (intravenous 5 mg/kg) of the Fc-prolactin fusions or the endogenously active, deglycosylated prolactin mutant (PRL-N59D). PRL N59D was dosed at ∼3-fold molar access over Fc-PRL-13 and Fc-PRL-17 and ∼4-fold molar access over Fc-PRL-3 and Fc-PRL-7. Then serum half-life (β-phase T_1/2_) was measured by ELISA (Figures S11A and S11D). The serum half-lives of the Fc-prolactin variants ranged from 35.1 h to 70.9 h (Figures S11A and S11D), which were all longer than PRL N59D (0.027 h). Fc-PRL-13 was identified as our top performing candidate because it had the longest *in vivo* half-life. Hereafter it is referred to as Prolactin-XL.

Prolactin-XL’s performance in initial *in vitro* screening and pharmacokinetics is summarized in Figure 1. Prolactin-XL’s key features include (1) a monomeric, non-glycosylated human prolactin mutant, (2) a GGsGG flexible linker, and (3) non-glycosylated, heterodimeric human Fc mutants with asymmetric binding to Protein A. This design scheme did not disrupt *in vitro* human or mouse PRLR signaling (Figure 1C). Likewise, Prolactin-XL’s binding to human FcRn was indistinguishable from isotype control (Figure 1E). But Prolactin-XL’s binding to off-target human and mouse FcRs was reduced (Figure 1G), especially in the presence of competing IgG (Figure S7B). Additionally, Prolactin-XL was more susceptible to degradation by classic GI proteases than isotype controls (Figures 1H–1I, S8, and S9). When compared to PRLN59D, Prolactin-XL had the most improved pharmacokinetics. In comparison to PRL N59D, Prolactin-XL has a 2,625-fold longer half-life (70.9 h vs. 0.027 h), 96-fold higher total drug exposure time (AUC_inf_), and 105-fold lower clearance rate (Figure 1J; Table 1). Based on these promising results, Prolactin-XL was carried forward for *in vivo* pharmacokinetic studies to further characterize its pharmacokinetic parameters in nulliparous mice, lactating mice, and nursing pups.

**Table 1:** Pharmacokinetic parameters of Prolactin-XL and Prolactin N59D in Tg276 nulliparous mice

Protein	N	AUC_inf_ (μg days/mL)	Clearance (mL/day)	β-phase T_1/2_ (h)	V_SS_ (mL/kg)
i.v. PRL N59D	4	24	4.2	0.027^a^	2.3
i.v. Prolactin-XL	5	2,305	0.04	70.9	0.39

a Data fit using one-phase decay; all other data were fit using two-phase decay.

Because of low expression of Prolactin-XL in Hek293F cells, we switched to producing Prolactin-XL in the yeast, *Komagataella phaffii* (formerly *Pichia Pastoris),* for subsequent animal experiments. Prolactin-XL was stably integrated into the genome of *Pichia*, and one clone, Fc-PRL-13-c6, was found to have a titer of ∼200 mg/L from a 3 mL culture in a 24-deep well plate as measured via ELISA (Figures S3A and S3B). Fc-PRL-13-c6 has a titer of ∼1 g/L from a 10 mL culture in a 125 mL flask as measured via ELISA (Figure S3B). Prolactin-XL has been mutated to remove all N-linked glycosylation sites, and we found no evidence of aberrant O-linked glycosylation via SDS-PAGE and western blots (Figures S3C–S3F). Fc-PRL-13-c6 has overexpression of the Fc-only monomer compared to the Fc-PRL monomer (Figures S3C–S3F). The Fc-only monomer of Fc-PRL-13 has a previously identified “RF” mutation to the Fc to ablate protein A binding, which, in theory, would prevent the overexpressed Fc-only monomer from being purified along with the correctly formed heterodimer.^23^^,^^34^ However, this is dependent on titer, purification conditions, and the version of commercially available Protein A used in the purification process.^23^^,^^34^ Low-titer Fc-PRL-13 from Hek293F expression has no identifiable Fc-only monomer contamination after protein A purification (Figures S1D and S2D), whereas the high-titer Fc-PRL-13 from *Pichia* has contaminating Fc-only monomers (Figures S3C–S3F). As such, ELISAs using purified Fc-PRL-13 from Hek293F (∼97% purity) as standards were used to measure Fc-PRL-13 concentrations. This Fc-only monomer is biologically inactive (i.e., does not bind to mouse FcRn or mouse FcRs) and is below the size cutoff for kidney clearance (70 kDa).

### Characterizing the pharmacokinetics of Prolactin-XL in nulliparous mice, lactating mice, and nursing pups

Prolactin-XL’s pharmacokinetics was next characterized in lactating Tg276 mice, as well as nulliparous and lactating wild-type C57bl/6j mice. Tg276 mice are difficult to breed, making lactating studies logistically challenging. In our breeding colony, ∼60% of the dams cannibalized their entire litter, and the pups of the remaining litters have stunted growth. Despite these challenges, we were able to dose six animals with a single dose (intravenous 5 mg/kg) of Prolactin-XL or PBS (Figures S11B and S11D). Prolactin-XL-treated mice did not cannibalize their pups, and no pups were euthanized due to weight loss compared to PBS controls. The serum half-life of Prolactin-XL in lactating Tg276 mice is 167.2 h, 2.4-fold longer than in nulliparous Tg276 mice (167.2 h vs. 70.9 h) (Figures S11B and S11D).

**Figure 2:**
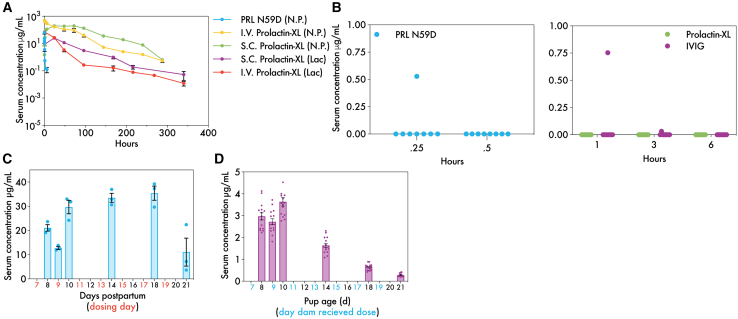
Pharmacokinetic profiles of Prolactin-XL in nulliparous mice, lactating mice, and nursing pups (A) C57bl/6j mice were injected with i.v. or s.c. administered 5 mg/kg of Prolactin-XL on the 7^th^ day postpartum, and litters were normalized to *n* = 5. Dose groups consist of nulliparous (N.P.) or lactating (Lac) mice (*n* = 5 for i.v. PRL N59D (N.P.), *n* = 8 for s.c. Prolactin-XL (N.P.), *n* = 5 for i.v. Prolactin-XL (N.P.), *n* = 5 for i.v. Prolactin-XL (Lac), and *n* = 7 for s.c. Prolactin-XL (Lac)). Blood was collected by tail nick post-injection, and the concentration of Prolactin-XL in serum was measured by ELISA. The data are depicted as mean ± SEM. PRISM was used to fit either a one-phase decay (PRL N59D or s.c. administered) or a two-phase decay (i.v. administered Fc-prolactin fusions). The relevant pharmacokinetic parameters are listed in Table 2. (B) Nulliparous C57bl/6j mice were administered 5 mg/kg of Prolactin-XL, IVIG, or PRL N59D fusions (*n* = 8) via oral gavage. Blood was collected by tail nick post injection, and the concentration of the proteins in serum was measured by ELISA. Prolactin-XL was undetectable (<100 ng/mL). The data are depicted as individual data points. Only 1/8 mice orally dosed with IVIG or PRLN59D had detectable serum levels, whereas the remaining 7/8 mice for both treatment groups had undetectable serum levels. (C) Lactating C57bl/6j mice (litters normalized to *n* = 5) were administered s.c. 5 mg/kg of Prolactin-XL every other day. Mice were sacrificed at six different time points (*n* = 3), and blood was collected by cardiac puncture. The concentration of Prolactin-XL in serum was measured by ELISA. The data are depicted as mean ± SEM. (D) The pups of the dams repeatedly dosed were also sacrificed (*n* = 15), and their blood was collected by decapitation. The concentration of the fusions in serum was measured by ELISA. The data are depicted as mean ± SEM.

Given the breeding challenges with Tg276 mice, we shifted to using more reliable breeders, the wild-type C57bl/6j (B6) mice, for further pharmacokinetic and galactopoietic studies. Although these mice express mouse FcRn, Prolactin-XL does not bind to it (Figure S6C). This means that Prolactin-XL’s extended half-life compared to PRL N59D in B6 mice is only attributed to decreased kidney clearance. Pharmacokinetic studies for a single dose (intravenous and subcutaneous at 5 mg/kg) of Prolactin-XL were conducted in nulliparous and lactating B6 mice (Figure 2A). The serum half-life of intravenous and subcutaneous Prolactin-XL in nulliparous mice was 26.8 h and 112 h, respectively (Figure 2A; Table 2). Without FcRn rescue from endosomal degradation, Prolactin-XL has a 157-fold increased half-life compared to PRL N59D (26.8 h vs. 0.17 h; Figure 2A; Table 2). Furthermore, Prolactin-XL’s half-life decreased by 2.6-fold compared to Tg276 mice with FcRn binding (70.9 h vs. 26.8 h; Figures S11A and S11D compared to S2A; Table 2). In contrast to Tg276 mice, the serum half-life of Prolactin-XL in B6 mice was 2.5-fold and 3.5-fold shorter in lactating mice than in nulliparous mice for intravenous and subcutaneous injections, respectively (intravenous: 10.9 h vs. 26.8 h and subcutaneous: 31.9 h vs. 112 h; Figure 2A; Table 2). These results confirm that FcRn binding contributes to Prolactin-XL’s extended half-life in nulliparous mice, but they also suggest that FcRn has additional roles in mediating Prolactin-XL’s pharmacokinetics in lactating mice.

**Table 2:** Pharmacokinetic parameters of Prolactin-XL in nulliparous and lactating B6 mice

Protein	N	AUC_inf_ (μg days/mL)	Clearance (mL/day)	β-phase T_1/2_ (h)	V_ss_ (mL/kg)
**Nulliparous**					

i.v. PRL N59D	5	9.0	11.1	0.17^a^	13.3
i.v. Prolactin-XL	5	15,227	0.0066	26.8	0.27
s.c. Prolactin-XL	8	22,708	0.0044	112^b^	0.32
Oral Prolactin-XL	8	0	0	0	0

**Lactating**					

i.v. Prolactin-XL	5	1,586	0.063	10.9^a^	0.88
s.c. Prolactin-XL	7	1,369	0.073	31.9^b^	1.13

a Data fit using one-phase decay; all other data were fit using two-phase decay.

b A one-phase decay was used to fit the data after peak serum concentration.

Although Prolactin-XL has a long serum persistence in mice, we next evaluated Prolactin-XL for its susceptibility to GI proteases *in vivo* to minimize GI absorption. A single dose of PRL N59D, Prolactin-XL, or intravenous IG (IVIG) (5 mg/kg oral gavage) was administered to adult mice and measured via ELISA. PRL N59D is dosed at a molar access of ∼3- and 7-fold over Prolactin-XL and IVIG, respectively. Notably, Prolactin-XL has 0% oral bioavailability in nulliparous Tg276 and B6 mice (detection limit is < 100 ng/mL) (Figures 2B and S11C). While PRL N59D was undetectable in Tg276 mice (<50 ng/mL), it was detectable in 1/8 B6 mice (Figures 2B and S11C). IVIG was detectable in 1/8 Tg276 mice and B6 mice (detection limit is 35 ng/mL) (Figures 2B and S11C). These results confirm that Prolactin-XL has long serum persistence but low GI absorption in adult mice.

Given Prolactin-XL’s enhanced susceptibility to GI proteases, we next evaluated Prolactin-XL’s susceptibility to serum proteases in adult mice. To assess this, we examined Prolactin-XL’s serum stability in lactating mice, which were treated every other day with 5 mg/kg subcutaneous Prolactin-XL and had their serum levels measured via ELISA (Figure 2C). The Fc-only monomer of Prolactin-XL was detectable in serum by western blot (Figure S12). The Fc-prolactin monomer of Prolactin-XL was undetectable in maternal serum via western blot likely due to serum albumin interfering with detection (Figure S12). No cleavage products were identifiable in the serum samples via western blot (Figure S12).These results indicate that Prolactin-XL’s susceptibility to GI proteases does not translate to increased serum proteolysis.

Because Prolactin-XL is long-lasting in circulation and has no oral bioavailability in adult mice, we next evaluated its systemic exposure in nursing pups. Prolactin-XL is detectable in the serum of B6 pups fed by dams repeatedly dosed with Prolactin-XL by ELISA (Figure 2D). Prolactin-XL serum levels in pups peak at ∼3.6 μg/mL, which is 12% of maternal Prolactin-XL serum levels on day 10 postpartum. By 21 days of age when the pup’s GI system most closely resembles that of human infants, the pups’ serum concentration of Prolactin-XL is ∼0.3 μg/mL, less than 3% of maternal serum concentrations (Figures 2C and 2D). These results suggest that Prolactin-XL’s serum concentrations may not reflect maternal serum levels and may depend on the maturation of the pup’s GI system.

Since Prolactin-XL enters the serum of nursing pups, we sought to determine if there were detectable cleavage products. In the serum of pups fed by dams given Prolactin-XL, the Fc-only monomer of Prolactin-XL was detectable by western blot on days 10, 14, and 18 of age (Figure S13). However, Prolactin-XL’s Fc-prolactin monomer was undetectable in the pups’ serum via western blot, and there were no identifiable cleavage products (compare Figures S12 and S13). Like in maternal serum and *in vitro* stability assays, there was no identifiable evidence of Prolactin-XL cleavage products.

### Prolactin-XL restores pharmacologically ablated galactopoiesis

After confirming Prolactin-XL is long-lasting and has low oral bioavailability, we evaluated Prolactin-XL’s *in vivo* galactopoietic effects in a mouse model of pharmacologically induced lactation insufficiency. Here, galactopoiesis was ablated by Bromocriptine (BR), a dopamine agonist that disrupts native pituitary prolactin secretion.^35^ In these experiments, lactating B6 dams were given galactopoietic-stimulating and/or galactopoietic-ablating treatments on day 7 postpartum. Litter numbers were normalized (*n* = 5) at day 7 postpartum. Pups were weighed daily, and pup weight was used as a surrogate measure of milk production. Pathologically underweight pups were sacrificed when they weighed 20% less than positive controls for ethical reasons; surviving pups were weaned at 21 days postpartum.

**Figure 3:**
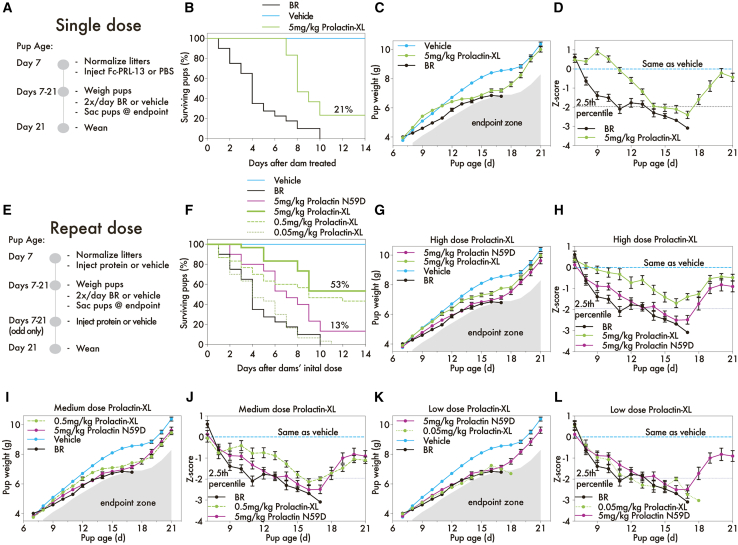
Prolactin-XL restores pharmacologically ablated galactopoiesis (A) A timeline of a single dose experiment where the B6 dams are dosed with BR or vehicle (0.2 mg tartaric acid dissolved in PBS with 20% ethanol) twice-daily (s.c.) and administered s.c. Prolactin-XL or vehicle (PBS) once. The dose groups are vehicle (*n* = 8), BR + 5 mg/kg Prolactin-XL (*n* = 6), or BR (*n* = 8). The pups reach endpoint when they weigh 20% less than the pups in the vehicle control group. (B) The percentage of pups remaining in the study each day was recorded. The data are depicted as mean ± SE. (C and D) (C) The weight of the pups was measured daily starting on day 7 postpartum (*n* = 40 for vehicle, *n* = 30 for BR + 5 mg/kg of Prolactin-XL, and *n* = 40 for BR) and (D) the *Z* score for each pup was calculated. For (C and D), the data are depicted as mean ± SEM. A two-way ANOVA with Bonferroni correction for multiple comparison was used to calculate the statistical significance of pup weight gain, and the *p* values are listed in Table S8. (E) A timeline of a repeat dose experiment where the B6 dams are dosed with BR or vehicle (0.2 mg tartaric acid dissolved in PBS with 20% ethanol) s.c. twice daily and s.c. administered Prolactin-XL, Prolactin N59D, or vehicle (PBS) every other day. The dose groups are vehicle (*n* = 8), Br + 5 mg/kg Prolactin N59D (*n* = 6), BR + 0.5 mg/kg Prolactin-XL (*n* = 6), BR + 0.05 mg/kg Prolactin-XL (*n* = 6), BR + 5 mg/kg Prolactin-XL (*n* = 6), or BR (*n* = 8). The pups reach endpoint when they weigh 20% less than the pups in the vehicle control group. (F) The percentage of pups remaining in the study was recorded each day. The data are depicted as mean ± SE. (G–L) (G, I, and K) The weight of the pups was measured daily starting on day 7 postpartum (*n* = 40 for vehicle, *n* = 30 for BR + 5 mg/kg of Prolactin N59D, *n* = 30 for BR + 0.05 mg/kg of Prolactin-XL, *n* = 30 for BR + 0.5 mg/kg of Prolactin-XL, *n* = 30 for BR + 5 mg/kg of Prolactin-XL, and *n* = 40 for BR), and the *Z* score for each pup was calculated (H, J, and L). For (G–I), the data are depicted as mean ± SEM. A two-way ANOVA with Bonferroni correction for multiple comparison was used to calculate the statistical significance of pup weight gain, and the *p* values are listed in Tables S9 and S10.

First, we evaluated Prolactin-XL’s duration of response. Here, we assessed Prolactin-XL’s ability to restore BR-ablated galactopoiesis with a single administration. Dams were injected twice daily with BR (0.2 mg) or vehicle (0.2 mg tartaric acid dissolved in PBS with 20% ethanol) starting on day 7 postpartum. All pups fed by vehicle-control-treated dams survive to 21 days and are weaned (Figure 3B). By contrast, pups fed by dams receiving twice-daily BR treatment were pathologically underweight (Figures 3C and 3D; Table S8). No pups survived until weaning; they all were sacrificed by day 17 (Figure 3B). However, a single dose of Prolactin-XL on day 7 postpartum increased milk production for 2 days in BR-treated dams, and 21% of pups survived until weaning (Figures 3B–3D; Table S8). Pups fed by +Prolactin-XL/+BR dams achieved maximum weight gain on day 9 postpartum and were 0.83 g heavier (*Z* score of 0.93 ± 0.18 SEM) than pups fed by vehicle-control-treated dams (Figures 3C and 3D; Table S8). These findings indicate that lactating mice respond to Prolactin-XL for a duration of 2 days as detected by changes in pup weight gain.

Next, we performed a dose-range finding study with repeat administration to evaluate the response vs. dose. Here, repeated dosing of Prolactin-XL in BR-treated dams increased pup weight and survival in a dose-dependent manner (Figures 3F–3L). The timing of Prolactin-XL treatment was every 2 days starting on day 7 postpartum. Prolactin-XL was given subcutaneously at a high dose (5 mg/kg), a medium dose (0.5 mg/kg), and a low dose (0.05 mg/kg). PRL N59D was dosed at ∼3-, 30-, and 300-fold molar access over the high, medium, and low doses of Prolactin-XL, respectively. Fifty-three percent of pups survived until weaning when their BR-treated dams received high doses of Prolactin-XL, compared to 13% of pups for equivalent mg/kg-dosing of PRL-N59D (Figure 3F). The weight of pups fed by dams in the BR+/PRL N59D + dose group was not statistically different than pups fed by BR-treated pups (Figures 3G–3L; Table S10). In contrast, the weight of pups fed by BR-treated dams given high doses of Prolactin-XL was statistically increased compared to pups fed by BR+/PRL N59D + dams on day 11, 13, 15, and 17 of age (Figures 3G and 3H; Table S10). By weaning, the surviving pups in the high-dose Prolactin-XL group achieved equivalent weight gain as pups fed by vehicle-control-treated dams (Figures 3G and 3H; Tables S9 and S10). These results suggest that high repeat doses of Prolactin-XL restore pharmacologically ablated lactation in mice.

### Natural weaning resolves prolactin-XL-stimulated galactopoietic morphological changes in mice with established milk production

We next assessed Prolactin-XL’s ability to stimulate galactopoiesis in lactating mice to evaluate if it could enhance established lactation without causing oversupply of milk or pathological pup weight gain. Here, the mice’s endogenous prolactin basal and suckling-induced secretion is intact.

**Figure 4:**
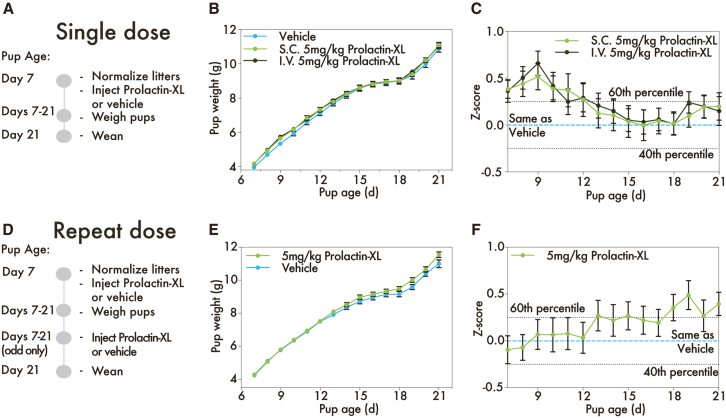
Prolactin-XL increases milk supply in mice with uncomplicated galactopoiesis (A) A timeline of single-dose experiment where B6 dams are given a single dose of subcutaneous or intravenous 5 mg/kg Prolactin-XL or vehicle (PBS) on day 7 postpartum. The dose groups are vehicle (*n* = 6), 5 mg/kg i.v. Prolactin-XL (*n* = 5), or 5 mg/kg s.c. Prolactin-XL (*n* = 7). (B and C) (B) The weight of the pups was measured daily starting on day 7 postpartum (*n* = 30 for vehicle, *n* = 25 for i.v. 5 mg/kg Prolactin-XL, and *n* = 35 for s.c. Prolactin-XL), and (C) the *Z* score for each pup was calculated. For (B and C), the data are depicted as mean ± SEM. A two-way ANOVA with Bonferroni correction for multiple comparison was used to calculate the statistical significance of pup weight gain, and the *p* values are listed in Table S11. On day 9 postpartum, the weight of pups fed by i.v. Prolactin-XL-dosed dams are significantly higher than the weight of pups fed by vehicle control dams. (D) A timeline of a repeat dose experiment where B6 dams are dosed with subcutaneously administered Prolactin-XL or vehicle every other day (*n* = 6 and *n* = 7, respectively). (E and F) (E) the weight of the pups was measured daily starting on day 7 postpartum (*n* = 30 for Prolactin-XL and *n* = 35 for vehicle), and (F) the *Z* score for each pup was calculated. For (E and F), the data are depicted as mean ± SEM. Multiple unpaired t tests with Bonferroni correction for multiple comparison were used to calculate the statistical significance of pup weight gain, and the *p* values are listed in Table S12.

First, we evaluated lactating mice’s duration of response to Prolactin-XL. Dams with established lactation were administered a single high dose (5 mg/kg, subcutaneous or intravenous) of Prolactin-XL or vehicle (PBS) on day 7 postpartum. Under these conditions, all pups survive to weaning. On day 2 post-injection (day 9 postpartum), the pups fed by intravenous Prolactin-XL-treated dams were statistically heavier by 0.4 g (*Z* score of 0.7 ± 0.6 SEM) than pups fed by vehicle-control-treated dams (Figures 4B and 4C; Table S11). At weaning, the weight of the pups fed by the Prolactin-XL-treated dams was comparable to pups fed by the vehicle control dams (Figures 4B and 4C; Table S11). Consistent with the findings from the pharmacologically ablated lactation studies, the results indicate that the response to Prolactin-XL is detectable for 2 days when measured via pup weight gain.

We next evaluated the duration of Prolactin-XL’s response with repeat administration over the last 2 weeks of lactation. Repeated high dosing of Prolactin-XL (every 2 days, 5 mg/kg, subcutaneous) appeared to nominally increase pup weight, although not statistically significantly. Pups weighed 0.6 g (*Z* score of 0.4 ± 0.1 SEM) more than pups fed by vehicle-control-treated dams at weaning (Figures 4E and 4F; Table S12). These findings suggest that Prolactin-XL stimulates galactopoiesis without causing pathological oversupply of milk or pathological weight gain of pups. However, the lactating mice’s response to Prolactin-XL may not be fully detectable via measuring changes in pup weight gain.

Unsurprisingly, the pup weight gain differential is small between pups fed by dams dosed with Prolactin-XL and pups fed by control mice because pup weight is a downstream effect of prolactin signaling and increased milk supply. In this setting where endogenous prolactin production is intact, pup appetite and suckling behaviors may moderate the effect of Prolactin-XL on pup weight gain. As such, we further investigated Prolactin-XL’s duration of response on upstream markers in the mammary gland via protein biomarker and morphological analyses.

Our protein biomarker analysis revealed no differences in key galactopoietic and involution proteins (Figure S14). Prolactin-XL is measurable in serum up to 40 μg/mL and penetrates mammary tissue as detected by ELISA (Figures 2C and S14A). In the mammary tissue of Prolactin-XL-treated dams, JAK2/STAT5 activation, levels of prolactin receptor, and levels of β-casein, a principal milk protein, are indistinguishable from vehicle controls as measured via western blot (Figures S14B–S14F and S14J–S14M). Phosphorylated STAT3 is a canonical biomarker of apoptotic alveolar cells during involution. pSTAT3 levels in mammary glands from mice dosed with Prolactin-XL are equivalent to vehicle controls as measured by western blot (Figures S14G–S14I and S14N–S14O). These findings suggest Prolactin-XL does not influence bulk measures of galactopoietic and involution biomarkers.

**Figure 5:**
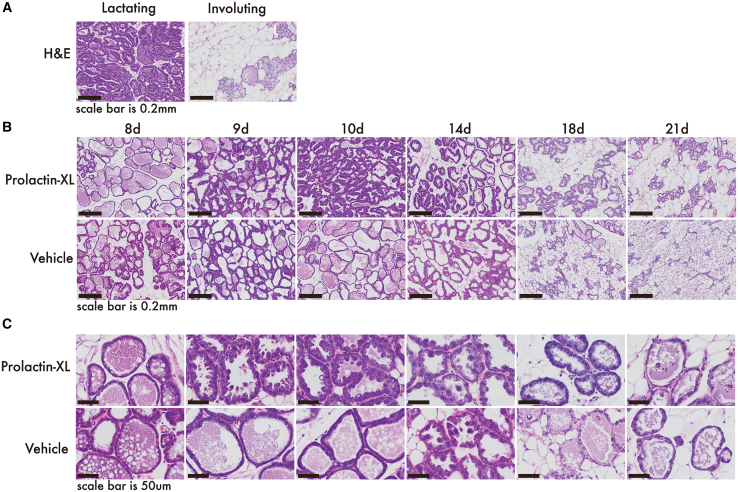
Prolactin-XL induces transient changes in mammary tissue in mice with uncomplicated galactopoiesis (A) Example snapshots of lactating (left) and involuting (right) mammary glands stained with H&E (scale bars: 0.2 mm). For (B and C), lactating mice (*n* = 3) were repeatedly dosed with subcutaneously administered vehicle (PBS) or Prolactin-XL (5 mg/kg) every other day beginning on the 7^th^ day postpartum. Litters were normalized (*n* = 5) on the 7^th^ day postpartum. Mammary glands from the lactating mice were collected on days 8, 9, 10, 14, 18, and 21 post-partum for subsequent analysis. Pups are weaned on the 21^st^ day postpartum. Whole mammary glands were formalin-fixed, paraffin-embedded, and stained with H&E (*n* = 3 per time point). Representative snapshots are depicted in (B) (scale bars: 0.2 mm) and (C) (scale bars: 50 μm).

Although our histological analysis found no Prolactin-XL-induced pathologies, it did find morphological features in the mammary gland consistent with transient hypersecretion. A board-certified rodent histopathologist found no pathologies in H&E-stained mammary tissues of Prolactin-XL-dosed dams. Instead, we found increased pup weight corresponds to transient, Prolactin-XL-dependent changes in dams’ mammary tissue. Prolactin-XL has a hypersecretory-galactopoietic effect on mammary morphology, most prominent on day 10 postpartum, which corresponds to peak milk production in mice. These effects include: (1) cells with cuboidal, bulbous, and hobnail appearances; (2) protrusion of the cell membrane into the lumen of alveoli, and (3) vacuolated cytoplasm (Figures 5B and 5C). These results show that lactating mice exhibit a morphological response to Prolactin-XL’s in the mammary gland while pups are fully nursing.

But the response does not persist during weaning and involution. The hypersecretory features induced by Prolactin-XL completely resolve between postpartum days 18 and 21 coincident with weaning and mammary gland involution (Figures 5B and 5C). Early morphological signs of the basement membrane remodeling indicative of involution begin to appear around 14 days postpartum in H&E-stained mammary glands from both Prolactin-XL- and vehicle-dosed mice with an increase in visible adipocytes (Figures 5B and 5C).^10^^,^^36^ Around this time, pups without stunted growth begin to try solid food between 14 and 16 days of age when their teeth erupt (i.e., gradual self-weaning). By day 18 postpartum, more classical morphological signs of the irreversible phase of involution are apparent in H&E-stained mammary glands from both Prolactin-XL- and PBS-dosed mice. These include (1) a decrease in hypersecretory features of alveolar cells (i.e., cuboidal, bulbous, and hobnailed cells) and vacuolated cytoplasm, (2) nuclear condensation, (3) shedding of apoptotic cells into the alveolar lumens, (4) alveolar collapse, and (5) re-filling of adipocytes into the basement membrane (Figures 5B and 5C).^10^^,^^36^ Pups without stunted growth can survive on solid food alone by day 21 of age, but we observe most litters still nursing at weaning. By day 21 postpartum, mammary glands from both dose groups similarly depict more profound architectural restructuring and hallmarks of involution as they are transitioning back to their pre-pregnancy state (Figures 5B and 5C).^10^^,^^36^ Therefore, Prolactin-XL does not morphologically alter the involution process.

## Discussion

Here, we engineer a long-acting human IgG1 Fc domain-human prolactin fusion, Prolactin-XL. Prolactin-XL has a serum half-life of 70.9 h in mice, 2,625-fold longer than endogenously active prolactin alone. We demonstrate in mice that Prolactin-XL stimulates milk production and restores growth of pups fed by dams with pharmacologically ablated galactopoiesis. When Prolactin-XL is repeatedly dosed at 3-fold molar less than PRL N59D over Prolactin-XL, 53% of pups survive until weaning when their BR-treated dams receive Prolactin-XL, compared to 13% of pups fed by BR-treated dams receiving PRL N59D. We further show that natural weaning resolves Prolactin-XL-stimulated galactopoietic morphological changes in the mammary glands of mice with established lactation.

Prolactin-XL’s promising results were built on the underlying rationale that a Fc fusion would create a long-lasting biologic. Multiple lines of evidence suggest that a Fc-prolactin fusion is a good candidate to enhance the pharmacokinetics of prolactin in comparison to the other methods. PEGylation can result in anti-PEG immune responses.^37^ Altering glycosylation inactivates prolactin.^27^ Fusing Fc or human serum albumin (HAS) to a protein will prevent renal clearance if the molecular weight of the fusion is greater than 70 kDa, as in the case with Prolactin. Fc and HSA are both endosomally recycled by binding neonatal Fc receptor (FcRn), resulting in longer half-lives.^13^ Fc and HSA are naturally secreted into breastmilk likely via FcRn-mediated transport.^38^ For every IgG molecule recycled by FcRn, ∼700 HSA molecules are recycled, meaning that the relative dose to an infant would be higher than for a Fc-prolactin fusion.^13^ Additionally, the half-life of an HSA-prolactin fusion would be lower than a Fc-prolactin fusion because more would be secreted in the breastmilk. We therefore opted for fusing Fc to prolactin as the approach to enhance prolactin’s pharmacokinetics. The one disadvantage to using Fc is that it can also bind and activate off-target FcRs on immune cells to elicit an immune response and clear Fc-contain proteins via FcR-mediated endosomal degradation. However, this can be avoided by mutating the Fc to ablate FcR binding.^24^^,^^30^^,^^31^

In principle, Prolactin-XL integrates the multiple functions and pharmacokinetics of both the Fc domain and prolactin to increase basal prolactin levels. Below, we briefly discuss relevant prolactin pharmacokinetics in humans before discussing Prolactin-XL’s pharmacokinetics in mice. For a comparison between the prolactin’s role in humans versus rodents, we refer the readers to a comprehensive review by Ben-Jonathan et al.^39^

Prolactin’s pharmacokinetic profiles are complex, with levels ranging from 10–30 ng/mL in non-pregnant/lactating persons and 100–400 ng/mL during pregnancy and lactation.^1^^,^^40^ It is primarily synthesized in the anterior pituitary but is also secreted by other tissues like the mammary gland.^41^^,^^42^ Prolactin, along with other hormones, helps to develop glandular tissue and differentiate alveolar cells for milk production.^10^^,^^43^^,^^44^^,^^45^ During lactation, prolactin secretion occurs via basal and suckling-induced peak modes in response to nipple stimulation.^1^^,^^46^^,^^47^^,^^48^^,^^49^^,^^50^ While nursing, nipple stimulation and milk removal triggers the suppression of dopamine and the release of prolactin-releasing factors in the hypothalamus, which in turn stimulates prolactin secretion from the anterior pituitary. In the mammary gland, prolactin signals to milk-producing cells to survive and produce many components of breastmilk. Prolactin is then cleared via passive kidney clearance, prolactin-receptor-mediated degradation, and secretion into breastmilk.^38^^,^^51^

To understand how Prolactin-XL may integrate prolactin and Fc pharmacokinetics in humans, we first conducted pharmacokinetics experiments in Tg276 mice, which are transgenic for human FcRn and homozygous knockout for mouse FcRn. Empirically, these mice outperform other *in vivo* models in predicting the human antibody pharmacokinetic properties.^24^^,^^52^ These mice have a similar molecular weight cutoff for passive kidney clearance (∼70 kDa) as humans.^13^ Furthermore, Prolactin-XL binds and actively signals via mouse PRLR in Tg276 mice. Prolactin-XL has a serum half-life of 70.9 h, 2,625-fold longer than endogenously active prolactin in Tg276 mice. Prolactin N59D (23 kDa) was rapidly cleared by the kidneys with a half-life of 0.02 h. In nulliparous B6 mice where Prolactin-XL does not bind mouse FcRn, the half-life of Prolactin-XL is shorter than in Tg276 mice (26.8 h vs. 70.9 h). Here, Prolactin-XL will have an increased half-life compared to PRL N59D only due to decreased passive kidney clearance. Mouse FcRn-binding could be compromised by the C-terminally fused prolactin monomer, the non-glycosylated Fc mutation, the asymmetric “RF” mutation, the ZW1 mutations to form heterodimeric Fc, or any combination thereof. These results suggest that reduced kidney clearance and endosomal degradation contribute to Prolactin-XL’s long-lasting serum persistence in nulliparous mice.

In lactating mice, FcRn may mediate Prolactin-XL’s half-life differently. Both human and mice mammary gland cells express PRLR and FcRn, so we expected Prolactin-XL to have a shorter half-life in lactating mice compared to nulliparous mice due to increased receptor-mediated degradation in the mammary gland and clearance via breastmilk.^53^^,^^54^ However, in lactating Tg276 mice, Prolactin-XL’s half-life is 2.4-fold longer than in nulliparous mice (167.2 h vs. 70.9 h). In B6 mice where Prolactin-XL does not bind mouse FcRn, the half-life was 2.5- and 3.5-fold shorter in lactating mice than nulliparous mice for intravenous and subcutaneous injections, respectively. These findings indicate that FcRn may affect Prolactin-XL’s pharmacokinetics with a different mechanism in lactation compared to nulliparity.

One possible explanation for our findings in Tg276 mice is that Fc may partially rescue Prolactin-XL from transmission into milk and from PRLR-mediated endosomal degradation. In mice, antibodies with higher affinity to FcRn tend to have lower transmission into breastmilk than those with lower affinity.^54^ For example, high affinity FcRn binding limits mouse IgG1 antibodies from secretion into mouse breastmilk compared to those with ablated FcRn binding.^54^ Here, we assume Prolactin-XL binds similarly *in vivo* to IgG1 antibodies, based on its comparable *in vitro* binding to IgG1 antibodies. FcRn in milk-making cells may recycle Prolactin-XL back into maternal tissue. Furthermore, on milk-making cells, PRLR is primarily located on the basolateral side, suggesting that PRLR-mediated transcytosis of Prolactin-XL to the apical side is likely limited.^55^^,^^56^^,^^57^ PRLR binding is pH-dependent, where prolactin is released in acidic endosomes. As such, we expect Prolactin-XL is largely degraded lysosomally after PRLR activation.^58^ But FcRn may rescue PRLR-bound Prolactin-XL from cellular degradation because it may bind to Prolactin-XL released from PRLR in acidic endosomes. This line of reasoning suggests that FcRn binding may partially rescue Prolactin-XL from receptor-mediated degradation and transfer into breastmilk. However, differences in pharmacokinetics may also arise from variation in lactation physiology or between mouse strains.

A key safety aspect of Prolactin-XL is its susceptibility to gastrointestinal proteases, which helps prevent infant uptake. Minimizing infant drug exposure is crucial, as lactating parents may stop breastfeeding due to concerns about infant exposure, and 60% of infants experience non-pathologic galactorrhea after birth.^59^^,^^60^^,^^61^^,^^62^ Here, we show the oral bioavailability of Prolactin-XL can be altered in mice. We measured Prolactin-XL levels in blood samples due to difficulties with obtaining sufficient milk samples for accurate quantitation.^63^^,^^64^^,^^65^ We found that Prolactin-XL has an oral bioavailability of 0% in both nulliparous Tg276 and C57bl/6j mice (detection limit is 100 ng/mL). Neonatal mice have a unique GI system that is more permeable to milk proteins like prolactin and IgG, with less digestion by GI proteases, enhanced macromolecule transfer via endocytic machinery, and delayed gut closure to macromolecular transfer, which occurs at 21 days of age.^66^ In contrast, the human gut “closes” *in utero*, leading to lower oral bioavailability of proteins.^66^ For example, IgG1s in humans have an oral bioavailability measured to be <25% in infants.^29^^,^^32^ In our experiments, when the pups are 21 days of age and their GI system most resembles that of human infants, the serum concentration in pups nursed for 2 weeks by Prolactin-XL-treated mice is less than 3% of maternal levels. Decreased Prolactin-XL levels in the pups may be related to factors such as reduced transfer of new molecules, elimination of older molecules, reduced milk intake as pups begin eating solid food at 14–16 days of age, or any combination thereof.

The importance of suckling-induced or basal prolactin secretion for lactogenesis and galactopoiesis remains an open question. However, our data support that high basal prolactin levels are likely sufficient for galactopoiesis in mice. Further, multiple lines of evidence suggest suckling-induced prolactin secretion is not essential for milk production in mammals when basal prolactin is high.^1^^,^^67^^,^^68^^,^^69^^,^^70^^,^^71^^,^^72^^,^^73^ For example, in mice and pigs with BR-ablated prolactin secretion, exogenous prolactin and/or dopamine receptor antagonists increase basal prolactin levels and rescue lactation.^67^^,^^68^^,^^69^^,^^70^^,^^71^^,^^72^^,^^73^ In humans, recombinant prolactin and dopamine receptor antagonists raise basal prolactin levels and enhance milk production, but they do not replicate the suckling-induced patterns of prolactin secretion.^1^^,^^12^^,^^73^^,^^74^^,^^75^ Here, our findings show that increasing basal prolactin levels by ∼1,000-fold over endogenous levels in BR-treated stimulates galactopoiesis.

In our studies, we used pup weight as a surrogate measurement for milk production because collecting daily milk volume is technically challenging and intractable in mice. Milking mice requires oxytocin injections and anesthesia, where administration is limited to once daily.^76^ In mice with pharmacologically inhibited prolactin secretion, the biologically active PRL N59D variant, dosed at ∼1,000-fold over endogenous prolactin levels, had no significant effect on pup weight gain compared to negative controls, whereas Prolactin-XL, dosed at 3-fold molar less, rescued pup weight gain, caused a higher rate of pup survival (53% vs. 13%), and increased pup weight compared to negative control and PRL N59D. However, in mice with established lactation, Prolactin-XL’s effect on pup weight gain was less pronounced. Pup weight gain is a surrogate measure for galactopoiesis and a downstream effect of enhanced milk production that is influenced by factors, such as pup satiety, pup stomach capacity, seasonal variation, and energy conversion to weight. Given this, Prolactin-XL’s impact on galactopoiesis may not be fully captured by changes in pup weight gain. Notably, we identified upstream morphological effects in the mammary gland of Prolactin-XL-treated mice, supporting its role in enhancing galactopoiesis without altering the involution process.

In sum, by combining antibody engineering with the approaches outlined here to design a longer acting prolactin, we have developed a potential tool for the study and pharmacological stimulation of galactopoiesis.

### Limitations of the study

Although galactopoiesis is largely conserved across mammals, it remains an open question how our results in mice would translate to human galactopoietic biology and would require extensive further research.

For example, future preclinical research should evaluate the off-target effects of Prolactin-XL, as PRLR is variably expressed across and between human and mouse tissues.^41^^,^^42^ Prolactin-XL might have off-target effects at extra-mammary sites. In this study, we did not characterize the biodistribution of Prolactin-XL or its off-target effects in mice. Future work is needed to characterize Prolactin-XL’s potential off-target effects in extra-mammary tissues. A key tissue for future evaluation of Prolactin-XL’s off-target effects is the brain. Prolactin crosses the blood-brain barrier (BBB) to influence the hypothalamic-pituitary-gonadal axis (HPG), impacting menstruation and lactation in humans and estrus, lactation, and breeding behavior in mice.^73^^,^^77^^,^^78^^,^^79^^,^^80^^,^^81^^,^^82^ The mechanism for how systemic prolactin crosses the BBB is unclear, as PRLR does not transport it in mice.^78^ It remains possible that Prolactin-XL may cross the BBB in a similar manner to endogenous prolactin. Alternatively, Prolactin-XL may cross the BBB via FcRn transcytosis. However, in mice FcRn typically mediates the efflux of antibodies out of the brain, thereby being a potential mechanism for preventing Prolactin-XL from crossing the BBB.^83^^,^^84^^,^^85^^,^^86^ Here, we did not determine how Prolactin-XL crosses the BBB, its levels in the brain, downstream biomarkers of activation like pSTAT5, or potential suppression of endogenous prolactin. Future work to characterize Prolactin-XL’s biodistribution and effect on endogenous prolactin is needed.

Multiple lines of clinical evidence suggest that off-target Prolactin-XL activation of PRLR in extra-mammary tissues may not be linked to serious adverse effects.^41^^,^^42^^,^^87^^,^^88^ For instance, hyperprolactinemia and treatment with r-PRL in non-lactating and lactating women are not associated with major adverse effects.^12^^,^^27^^,^^73^^,^^77^^,^^89^ In a phase 1 trial, r-PRL was administered to non-pregnant and non-lactating women to achieve 100–300 ng/mL serum levels. This treatment did not affect pituitary TSH secretion, though hypothalamic GnRh secretion was suppressed, while menstruation cyclicity remained unaffected.^77^ Similarly, in another phase 1 trial, r-PRL treatment achieving similar serum levels did not affect menstruation cyclicity or biomarkers of bone turnover in non-pregnant and non-lactating women.^73^ Furthermore, a phase 2 clinical trial treating lactating parents with r-PRL reported no serious side effects in the parents or breastfed infants.^12^^,^^89^ Taken together, these clinical findings suggest that off-target activation of PRLR in various tissues is not associated with serious toxicity. While our studies of Prolactin-XL in mice may not fully predict human or other species response to long-lasting prolactin, Prolactin-XL and homologs remain promising candidates to pursue further preclinical toxicological evaluations.

## Resource availability

### Lead contact

Further information and requests for resources and reagents should be directed to and will be fulfilled by the lead contact, Kasia Kready (kasiakready@g.harvard.edu).

### Materials availability

Materials generated in this study are available from the lead contact upon request.

### Data and code availability

• All data reported in this paper will be shared by the lead contact upon request.
• This paper does not report original code.
• Any additional information required to reanalyze the data reported in this paper is available from the lead contact upon request.

## Acknowledgments

This research was sponsored by the Blavatnik Biomedical Accelerator at 10.13039/100007229Harvard University and the Wyss Institute for Biologically Inspired Engineering. K.K. is supported by a Herchel Smith Graduate Fellowship, a Pharmacological Sciences Training Grant (5T32GM132089-03), and a Fuji Fellowship. K.D. is also supported by a Fuji Fellowship. We thank Dana-Farber/Harvard Cancer Center in Boston, MA, for the use of the Rodent Histopathology Core, which provided preparation of histology slides, slide interpretation, and histopathological interpretation services. Dana-Farber/Harvard Cancer Center is supported in part by an 10.13039/100000054NCI Cancer Center Support Grant #10.13039/100000002NIH 5 P30 CA06516. The authors would also like to thank the Neurobiology Imaging Facility at Harvard Medical School, Boston, MA for imaging histology sections. We sincerely thank Amanda Graveline, Andyna Vernet, Sarai Bardales, and Melinda Sanchez from the Wyss Institute at Harvard for their technical support with the animal experiments.

## Author contributions

K.K., K.C., and J.W. conceived of the long-acting, Fc-prolactin fusion. K.K. and J.W. designed initial constructs, and K.K. and K.D. designed experiments. K.D., K.C., J.W., C.P., and Q.J. gave technical support and conceptual advice. K.K. performed all experiments, analyzed data, and wrote the main paper with Q.J., C.P., and P.S. K.K., C.P., and P.S. wrote the Supplementary Information. All authors discussed the results and implications and commented on the manuscript. C.P. and P.S. supervised the project.

## Declaration of interests

K.K., K.C., J.W., and P.S. are co-inventors on a patent relating to this work. K.K., P.S., and C.E.P. are co-founders of Coralie Bio, Inc. that may benefit financially from the work described in this article.

## STAR★Methods

### Key resources table

**Table:** 

REAGENT or RESOURCE	SOURCE	IDENTIFIER
**Antibodies**		

Anti-6xHis-HRP	Abcam	RRID: AB_298652
HRP-conjugated anti-human IgG1 Fc	Invivogen	RRID:AB_429693.
Anti-human PRL antibodies	Abcam	RRID: AB_3677343
APC anti-Flag-tag antibody	Biolegend	RRID: AB_2561497
Goat anti-human IgG Fc antibodies	Abcam	RRID: AB_10681074
Anti-prolactin receptor	Abcam	RRID: AB_2941082
Anti-β-casein	Thermo Fisher	RRID: AB_1016740
Anti-STAT5	Cell Signaling Technologies	RRID: AB_2737403
Anti-pSTAT5	Cell Signaling Technologies	RRID: AB_2315225
Anti-STAT3	Cell Signaling Technologies	RRID: AB_2629499
Anti-pSTAT3	Cell Signaling Technologies	RRID: AB_3675996
Anti-β-actin	Cell Signaling Technologies	RRID: AB_2242334
Anti-mouse-HRP	Thermo Fisher	RRID: AB_228307
Anti-rabbit-HRP	Thermo Fisher	RRID: AB_228341

**Chemicals, peptides, and recombinant proteins**		

Human Prolactin Wildtype	Uniprot	P01236
Mouse Prolactin Wildtype	Uniprot	P06879
Human Prolactin Receptor	Uniprot	P16471
Mouse Prolactin Receptor	Uniprot	Q08501
Human Fc Wildtype	Uniprot	P0DOX5
Human FcgRI	Uniprot	P12314
Human FcgRIIa	Uniprot	P12318
Human FcgRIIb	Uniprot	P31994
Human FcgRIIIa	Uniprot	P08637
Mouse FcgRI	Uniprot	P26151
Mouse FcgRIIb	Uniprot	P08101
Mouse FcgRIII	Uniprot	P8508
Mouse FcgRIV	Uniprot	A0A0B4J1G0
Mouse IL-3	PeproTech	213-13
IgG1 isotype control	Thermo Fisher Scientific	31154
Trypsin	Promega	V5111
Chymotrypsin	Promega	V1061
Pepsin	Promega	V1959
IVIG	Creative Biomart	THP-0108
Bromocriptine mesylate	Sigma	1076501
Tartaric acid	Sigma	PHR1472
Dipotassium phosphate	Sigma	60353
Monopotassium phosphate	Sigma	P5655
Yeast extract	Gibco	212750
Peptone	Sigma	P5905
Yeast nitrogenous base without amino acids	Sigma	Y0626
L-glutathione	Sigma	G4251
Glycerol	Sigma	G5516
Methanol	Fisher Scientific	AC325740025
cOmplete Protease Inhibitor Cocktail	Sigma	11836145001
Pepstatin A	Sigma	P5318
WST-1	Thomas Scientific	C755B06
MTS	Abcam	ab197010
Tween 20	Sigma	P2287
Phosphatase inhibitor cocktail	Thermo Fisher	A32957

**Critical commercial assays**		

BCA assay	Thermo Fisher Scientific	23227

**Experimental models: Cell lines**		

Ba/F3	Dr. Jungmin Lee at HMS	RRID:CVCL_0161
Hek293-T	Dr. Rui Truong at HMS	RRID:CVCL_0045
Hek293-F	Dr. Jungmin Lee at HMS	RRID:CVCL_6642

**Experimental models: Organisms/strains**		

NRRL Y-11430	ATCC	76273
Tg276 mice	Jackson	004919
C57bl/6j	Jackson	000664

**Recombinant DNA**		

pSecTag2A plasmids	Dr. Jungmin Lee at HMS	
pPICZ A plasmids	Amazir Bredl at HMS	
pTwist Lenti Puro SFFV WPRE	Twist	
psPAX2	Addgene	12260
pMD2.G	Addgene	12259

**Software and algorithms**		

Unicorn v.6.3 software		N/A
GraphPad Prism		N/A
Image Lab Software		N/A

**Other**		

FreeStyle 293 Expression Medium	Thermo Fisher Scientific	12338026
DMEM	ATCC	30-2002
FBS	Thermo Fisher Scientific	10082147
Penicillin-Streptomycin	Thermo Fisher Scientific	15140122
Sodium Pyruvate	Thermo Fisher Scientific	11360070
MEM Non-essential Amino Acids	Thermo Fisher Scientific	11140050
HEPES	Thermo Fisher Scientific	15630080
RPMI 1640	ATCC	30-2001
293Fectin	Thermo Fisher Scientific	12347019
SimplyBlue	Thermo Fisher Scientific	LC6065
His60 Ni Superflow resin	TaKaRa	635677
Protein A agarose resin	Thermo Fisher Scientific	20334
Tris-glycine gels, 4–20%	Thermo Fisher Scientific	XP04205BOX
Endotoxin-free PBS	Teknova	P0300
Macrosep Advanced centrifugal devices	VWR	891310980
Superdex 200 Increase 10/300GL column	Cytiva	28990944
G418	Invivogen	ant-gn
Zeocin	Invivogen	ant-zn
MaxiSorp 96-well ELISA plates	Sigma	M9410
TMB Substrate Solution	Thermo Fisher Scientific	34028
2× Laemmli	Bio Rad	1610737
Puromycin	Sigma	P9620
Lipofectamine3000	Thermo Fisher Scientific	L3000008
P3000 reagent	Thermo Fisher Scientific	L3000008
Opti-Mem	Thermo Fisher Scientific	31985070
0.45um Durapore PVDF membrane steriflip	Coleparmer	EW-29969-26
4× Lenti-X concentrator reagent	TaKaRa	631231
Polybrene	Sigma	TR-1003-G
Human serum	Millipore Sigma	H6914-20ML
4× Nupage LDS sample buffer	Thermo Fisher Scientific	NP0007
Bis-Tris gels, 12%	Thermo Fisher Scientific	XP04205BOX
MES buffer	Thermo Fisher Scientific	NP0002
Nitrocellulose membrane	Thermo Fisher	IB23002
iblot2 Dry Blotting System	Invitrogen	IB21001
SuperSignal West Femto Maximum Sensitivity Substrate	Thermo Fisher Scientific	34095
RNALater	Thermo Fisher	AM702
RIPA Lysis buffer	Cell Signaling Technology	9806
SuperSignal West Dura Extended Duration Substrate	Thermo Fisher	34075

### Experimental model and study participant details

#### Mouse strains

Tg276 and C57bl/6j mice were used in this study (aged 7 days to 12 weeks). The animal work was approved by the Harvard Medical School IACUC under protocol IS00003310. The protocols for maintenance and care are reported below. Only female mice and their pups were used for this study. Male mice do not have nipples and cannot lactate.

#### Mammalian cell lines

Hek293F, Hek293T, and Ba/F3 cell lines were used in this study. The protocols for maintenance and care are reported below. The cell lines tested negative for mycoplasma, but they were not authenticated.

#### Yeast strains

Pichia Pastoris strain, NRRL Y-11430, was used for this study. The protocols for maintenance and care are reported below.

### Method details

#### DNA constructs

The DNA sequence for human PRL wild-type was derived by reverse translating and codon optimizing the protein sequence from UniProt (P01236). The PRL deglycosylated mutant N59D was constructed by introducing a codon change in the wild-type sequence. The DNA sequence for mouse PRL wild-type was derived by reverse translating and codon optimizing the protein sequence from UniProt (P06879). The DNA sequence for human and mouse PRLR wild-type was derived by reverse translating and codon optimizing the protein sequences from UniProt (P16471 and Q08501, respectively). The DNA sequence for human IgG1-Fc wild-type was derived by reverse translating and codon optimizing the protein sequence from UniProt (P0DOX5). It was modified (C220S) to remove the unpaired cysteine in the hinge region and a protease cleavage site at the c-terminus (K447A) by making a codon change in the wild-type sequence. This modified IgG1-Fc sequence was used as the base for the Fc-PRL fusions. Fc-PRL fusions were generated by fusing the IgG1-Fc sequence to PRL N59D. The following features of the Fc-PRL fusions were varied and introduced into the Fc-PRL fusions by codon changes.

(1) **The order of PRL fused to Fc** (i.e., N-PRL-Fc-C or N-Fc-PRL-C)
(2) **Use of linker between prolactin and Fc** (GGsGG)
(3) **Use of Fc heterodimer (**Fc Knob (T266W) fused to Fc Hole (T366S, L368A, Y407V)^20^^,^^21^ or Fc Zw1 A (T350V, L351Y, F405A, Y407V) fused to Fc Zw1 B (T350V, T366L, K293L, T394W)^22^
(4) **Fc-hole without Protein A binding to get rid of unwanted homodimers** (‘RF’ mutations: H435R, Y435F)^23^
(5) **Use of mutations to Fc to enhance half-life** (i.e., “EDHS” mutations: V264E, L309D, Q311H, N434S or “YTE” mutations: M252Y, S254T, T256E)^24^
(6) **Use of mutations to Fc to decrease FcR binding** (i.e., “LALA” mutations: L234A, L235A or “LALAPG” mutations: L234A, L235A, P329G)^25^

The DNA sequence for human FcgR1, FcgRIIa, FcgRIIb, and FcgRIIIa were derived by reverse translating and codon optimizing the protein sequences from UniProt (P12314, P12318, P31994, and P08637 respectively). The DNA sequence for mouse FcgRI, FcgRIIb, FcgRIII, and FcgRIV were derived by reverse translating and codon optimizing the protein sequence from UniProt (P26151, P08101, P8508, and A0A0B4J1G0, respectively). See Table S13 for individual amino acid sequences used in this study.

#### Mammalian cell culture

FreeStyle 293-F and Ba/F3 cell lines were obtained from Dr. Jungmin Lee (Harvard Medical School), and Hek293-T cell lines were obtained from Dr. Rui Truong (Harvard Medical School).

FreeStyle 293-F cell lines were cultured in FreeStyle 293 Expression Medium (Thermo Fisher Scientific; 12338026). Hek293-T cell lines were cultured in DMEM (ATCC; 30–2002) supplemented with 10% FBS (Thermo Fisher Scientific; 10082147), 1% Penicillin-Streptomycin (Thermo Fisher Scientific; 15140122), 1× Sodium Pyruvate (Thermo Fisher Scientific; 11360070), 1× MEM Non-essential Amino Acids (Thermo Fisher Scientific; 11140050), and 1× HEPES (Thermo Fisher Scientific; 15630080). Ba/F3 cell lines were cultured in RPMI 1640 (ATCC; 30–2001) supplemented with 10% FBS (Thermo Fisher Scientific; 10082147), 1% Pen-Strep (Thermo Fisher Scientific; 15140122), and 10ng/mL mouse IL-3 (PeproTech; 213-13), unless specified otherwise. FreeStyle 293-F cells were cultured at 37C in 8% CO2 with shaking at 125 RPM (volumes 30-90mLs) and 90 RPM (volumes120-1000mL). HEK 293T cells and Ba/f3 cells were cultured at 37C in 5% CO2.

#### Recombinant protein expression and purification from mammalian cells

For small batches (<10ugs), proteins were transiently expressed in FreeStyle 293-F cells using pSecTag2A plasmids and 293Fectin transfection reagent according to the supplier’s protocol (Thermo Fisher Scientific; 12347019). 4–6 days after transfection, protein levels in the supernatant were assayed by SimplyBlue (Thermo Fisher Scientific; LC6065) stained SDS-PAGE and western blots using either anti-6xHis-HRP antibodies (Abcam; ab1187) or HRP-conjugated anti-human IgG1 Fc (Invivogen; 31413). Proteins from transient transfections were either purified via His-tag or Protein A purification. His-tagged proteins were purified with His60 Ni Superflow resin (TaKaRa; 635677) according to the manufacturer’s instructions, and Fc fusions were purified with Protein A agarose resin (Thermo Fisher Scientific; 20334) according to the manufacturer’s instructions. Cell supernatant and each purification fraction were run on Tris-glycine gels, 4–20% (Thermo Fisher Scientific; XP04205BOX) under reducing and denaturing conditions, and then stained with SimplyBlue (Thermo Fisher Scientific; LC6065). Fractions containing eluted proteins were combined, concentrated, and de-salted into endotoxin-free PBS (Teknova; P0300) using Macrosep Advanced centrifugal devices (VWR; 891310980). 250nM of each purified Fc-PRL fusions were run on Tris-glycine gels, 4–20% 4–20% (Thermo Fisher; XP04205BOX) under reducing + denaturing and non-reducing conditions. The purity of the top 4 Fc-PRL fusions (Fc-PRL 3,7, 13, and 17) were confirmed by size exclusion chromatography and was over 95%. Protein concentration was measured by BCA assay (Thermo Fisher Scientific; 23227) according to the manufacturer’s instructors. Proteins were stored at 4C throughout the described process, ultimately stored as single-use aliquots at −80C and thawed once before use. Only endotoxin-free reagents were used. Size exclusion chromatography (SEC) was performed using a Superdex 200 Increase 10/300GL column (Cytiva; 28990944) on an Äkta Pure 25 HPLC system and analyzed using Unicorn v.6.3 software. Mammalian expressed proteins were used for all *in vitro* assays and for pharmacokinetic studies in Tg276 mice.

#### Yeast strains and cultivations

NRRL Y-11430 was obtained from (ATCC; 76273). Strains were generated to co-express the heterodimeric Prolactin-XL fusion using pPICZ A obtained from Amazir Bredl (Harvard Medical School). Competent cells were prepared and transformed as described elsewhere.^90^ Strains were first generated to express the Fc-only monomer under the control of the *DAS2* promoter and with Kanamycin resistance. Cells were allowed to recover overnight at room temperature without shaking and then plated on YPD agar plates supplemented with 200ug/mL G418 (Invivogen; ant-gn). Plates were incubated at 30C until colonies formed. All the colonies were then scraped off the plate, made competent, and transformed to express the Fc-PRL monomer under the control of the *AOX1* promoter and Zeocin resistance. Cells were allowed to recover overnight at room temperature without shaking and then plated on YPD agar plates supplemented with 200ug/mL G418 and 200ug/mL Zeocin (Invivogen; ant-zn). Plates were incubated at 30C until colonies formed.

For protein production from the strains, BMGY and BMMY media was prepared. 1L of BMY media was prepared with 900mL of miliQ water, 100mL of 10× Potassium Phosphate Buffer (22.99g dipotassium phosphate (Sigma; 60353) and 118.14 g monopotassium phosphate (Sigma; P5655) in 1L of miliQ water, pH 6.5), 10g yeast extract (Gibco; 212750), 20g peptone (Sigma; P5905), 13.4g yeast nitrogenous base without amino acids (Sigma; Y0626), and 3.07g of L-glutathione (Sigma; G4251). BMY media was then sterilized in the autoclave, and after cooling it was filtered using a 0.2uM filter. To prepare 1L of BMGY media, 40mL of 100% glycerol (Sigma; G5516) was added to BMY media. To prepare 1L of BMMY media, 15mL of methanol (Fisher Scientific; AC325740025) was added to BMY media. To screen for protein expression, colonies co-expressing both plasmids and the WT strain were grown in 24-well deep well plates (25C, 300RPM) with 3mL of BMGY media for 24h of biomass accumulation. Cells were then pelleted and resuspended in 3mL of BMMY media and allowed to grow for an additional 24 h. Cells were pelleted and samples of the supernatant were collected to analyze expression of Prolactin-XL via ELISA.

To quantify the amount of Prolactin-XL expressed by the strains, 50ul of 0.1ug/mL anti-human PRL antibodies (Abcam; ab244037) were diluted in PBS and used to coat MaxiSorp 96-well ELISA plates (Sigma; M9410) for 1h at room temperature. Plates were washed 3 times in PBST (PBS with 0.05% Tween 20 (Sigma; P2287), and then the plates were blocked with 200uL of blocking buffer (PBS with 3% BSA) overnight at 4C. Plates were again washed 3 times in PBST. 50uL of supernatant diluted in PBS (1/100, 1/1000, 1/10,000) was added to the plates and incubated at room temperature for 1h. Plates were then washed 5 times in PBST. 50uL of HRP-conjugated anti-human IgG1 Fc (Invivogen; 31413) diluted 1:5000 in blocking buffer was added to the plates and incubated at room temperature for 1h. Plates were then washed 5 times with PBST. 100uL of TMB Substrate Solution (Thermo Fisher Scientific; 34028) equilibrated to room temperature was added to the plates and incubated for 5.5 min or until desired color develops. Reactions were stopped by adding 100uL of 2M Sulfuric Acid to each well. The absorbance was measured at 450nm. A standard curve was generated by serially diluting purified Prolactin-XL into PBS. Prism (GraphPad Prism) was used to fit a sigmoidal curve to the data and used to quantify Prolactin-XL in the supernatant samples. The clone with the highest expression of Prolactin-XL was chosen for large-scale protein production.

#### Recombinant protein expression and purification from yeast

For large-scale batches, Prolactin-XL was produced from strain Prolactin-XL-c6. Prolactin-XL-c6 was grown in 3, 10, or 200mL of BMGY media in 24-deep well plates, 125mL baffle flask, or 2L baffled flask, respectively. 200mL cultures were supplemented 1tab/50mL of cOmplete Protease Inhibitor Cocktail (Sigma; 11836145001) and 10uM Pepstatin A (Sigma; P5318) in baffled 2L flasks. Cells were incubated for 24h of biomass accumulation at 25C and 300RPM. Cells were then pelleted and resuspended with 200mL of BMMY media supplemented with 1tab/50mL of O Complete Protease Inhibitors and 10uM Pepstatin A. Cells were again incubated for 24h of biomass accumulation at 25C and 300RPM. Prolactin-XL levels in the supernatant were assayed by Coomassie Blue stained SDS-PAGE, Western blots using HRP-conjugated anti-human IgG1 Fc (Invivogen; 31413) diluted 1:10000 in blocking buffer, or ELISA using anti-PRL capture antibodies and anti-IgG Fc detection antibodies as previously described. Prolactin-XL was purified with Protein A agarose resin (Thermo Fisher Scientific; 20334) according to the manufacturer’s instructions with the exception that all buffers were supplemented with 1tab/50mL of cOmplete Protease Inhibitor Cocktail and 10uM Pepstatin A. Cell supernatant and each purification fraction were run on Tris-glycine gels, 4–20% (Thermo Fisher; XP04205BOX) under reducing and denaturing conditions, and then stained with SimplyBlue (Thermo Fisher Scientific; LC6065). Fractions containing eluted proteins were combined, concentrated, and de-salted into endotoxin-free PBS (Teknova; P0300) using Macrosep Advanced centrifugal devices (VWR; 891310980). Protein concentration was measured by using a quantitative ELISA as previously described and using a purified standard from mammalian expression. Proteins were stored at 4C throughout the described process, ultimately stored as single-use aliquots at −80C and thawed once before use. Only endotoxin-free reagents were used. Prolactin-XL produced from yeast was only used *in vivo* in C56bl/6j mice.

#### ELISA assay of human or mouse FcRn binding

50uL of Fc-PRL fusions or IgG1 isotype control (Thermo Fisher Scientific, 31154) were diluted in PBS to 2ug/mL were used to coat MaxiSorp 96-well ELISA plates (Sigma; M9410) and incubated overnight at 4c. Plates were then washed 3 times with PBST (PBS with 0.05% Tween2 (Sigma; P2287)), and then the plates were blocked with 200uL of blocking buffer (PBS with 3% BSA) for 1h at room temperature. Plates were again washed 3 times with PBST. Plates were then incubated 1h at room temperature with 50uL serially diluted His-tagged human FcRn (starting at 400nM and diluted 1:2) or mouse FcRn (starting at 500nM and diluted 1:2). Plates were then washed 3 times with PBST (pH 7.4 or 5.8). 50uL of anti-6xHis-HRP antibody (Abcam; ab1187) diluted 1:10000 in PBS (pH 7.4 or pH 5.8) was added to the plates and incubated 1h at room temperature. Plates were then washed 3 times with PBST (pH 7.4 or 5.8). 50uL of TMB Substrate Solution (Thermo Fisher Scientific; 34028) equilibrated to room temperature was added to the plates and incubated for 1min or until desired color develops. Reactions were stopped by adding 50uL of 2M Sulfuric Acid to each well. The absorbance was measured at 450nm. Prism (GraphPad Prism) was used to fit a sigmoidal curve to the data and used to determine the Log (EC_50_) of the pH 5.8 curves only. Reported data represent mean ± SEM of three replicates.

#### ELISA assay of FcR binding

50uL of Fc-PRL fusions or IgG1 isotype (Thermo Fisher Scientific, 31154) were control diluted in PBS to 2ug/mL were used to coat MaxiSorp 96-well ELISA plates (Sigma; M9410) and incubated overnight at 4c. Plates were then washed 3 times with PBST (PBS with 0.05% Tween2 (Sigma; P2287)), and then the plates were blocked with 200uL of blocking buffer (PBS with 3% BSA) for 1h at room temperature. Plates were again washed 3 times with PBST. Purified His-tagged human and mouse FcRs were prepared as previously described. Plates were then incubated 1h at room temperature with 50uL serially diluted FcRs starting at 400nM and diluted 1:2. Plates were then washed 3 times with PBST. 50uL of anti-6xHis-HRP antibody (Abcam; ab1187) diluted 1:10000 in PBS was added to the plates and incubated 1h at room temperature. Plates were then washed 3 times with PBST. 50uL of TMB Substrate Solution (Thermo Fisher Scientific; 34028) equilibrated to room temperature was added to the plates and incubated for 1min or until desired color develops. Reactions were stopped by adding 50uL of 2M Sulfuric Acid to each well. The absorbance was measured at 450nm. Reported data represent mean ± SEM of three replicates.

#### ELISA assay of FcgRI binding with competing IgG1 isotype

50uL of Fc-PRL fusions or IgG1 isotype (Thermo Fisher Scientific, 31154) were control diluted in PBS to 2ug/mL were used to coat MaxiSorp 96-well ELISA plates (Sigma; M9410) and incubated overnight at 4c. Plates were then washed 3 times with PBST (PBS with 0.05% Tween2 (Sigma; P2287)), and then the plates were blocked with 200uL of blocking buffer (PBS with 3% BSA) for 1h at room temperature. Plates were again washed 3 times with PBST. Purified His-tagged human FcgRI was prepared as previously described. Plates were then incubated 1h at room temperature with 50uL serially diluted FcgRI (starting at 1uM and diluted 1:2 in PBS containing 2ug/mL IgG1 Isotype control (Thermo Fisher Scientific, 31154). Plates were then washed 3 times with PBST. 50uL of anti-6xHis-HRP antibody (Abcam; ab1187) diluted 1:10000 in PBS was added to the plates and incubated 1h at room temperature. Plates were then washed 3 times with PBST. 50uL of TMB Substrate Solution (Thermo Fisher Scientific; 34028) equilibrated to room temperature was added to the plates and incubated for 1min or until desired color develops. Reactions were stopped by adding 50uL of 2M Sulfuric Acid to each well. The absorbance was measured at 450nm. Reported data represent mean ± SEM of three replicates.

#### *In vitro* protease degradation study

Trypsin (Promega; V5111), chymotrypsin (Promega; V1061), and pepsin (Promega; V1959) were resuspended according to the manufacturer’s instructions. Trypsin, chymotrypsin, or pepsin were added on ice to Fc-PRL fusions or IgG1 isotype control at 1:1000, 1:100, and 1:5000, respectively. Samples were vortexed for 10s, and then a 3-5μL aliquot was taken for timepoint 0. Reactions were then incubated on a thermocycler at 25C (for chymotrypsin) or 37C (for trypsin and pepsin). 3-5μL aliquots were taken at 30min, 60min, 3h, and 6h and stored at −20C. 2× Laemmli (Bio Rad; 1610737) with 5% BME was added to all aliquots at 1:1 ratio and boiled at 100C for 10min. The aliquots were run on Tris-glycine gels, 4–20% (Thermo Fisher; XP04205BOX) under reducing and denaturing conditions, and then stained with SimplyBlue (Thermo Fisher Scientific; LC6065). The percent of remaining protein was analyzed by densitometry on Image Lab software. Reported data represent mean ± SEM of three replicates.

#### PRLR signaling cell-based assay

Stable cell lines expressing human or mouse PRLR were generated by lentiviral transduction followed by 0.7 μg/mL puromycin selection (Sigma; P9620). 12ug of each pTwist Lenti Puro SFFV WPRE lentiviral construct encoding either human or mouse PRLR were co-transfected with 9ug of psPAX2 (Addgene; 12260) and 3ug of pMD2.G (Addgene; 12259) 2^nd^ generation lentiviral packaging plasmids into HEK 293T cells at 60–80% confluency with 60uL Lipofectamine3000 (Thermo Fisher Scientific; L3000008) and 50uL P3000 reagent (Thermo Fisher Scientific; L3000008) diluted into 2.5mL of Opti-Mem (Thermo Fisher Scientific; 31985070). After 4h, the media was aspirated and replaced with 15mL of culture media (DMEM, 10% FBS, 1% PS, 1× sodium pyruvate, 1× MEM NEA, and 1× HEPES). Supernatant containing lentivirus was collected after a subsequent 24h and 48h and combined. Lentivirus-containing supernatant was centrifuged at 400xg for 5 min to pellet cell debris and then filtered with a 0.45um Durapore PVDF membrane steriflip (Coleparmer; EW-29969-26). 4× Lenti-X concentrator reagent (TaKaRa; 631231) was added to the lentivirus-containing supernatant at a final concentration of 1× and incubated at 4C for 1h. After incubation, the samples were centrifuged at 1500xg for 45min. Then the supernatant was removed, and the white virus pellet was resuspended in 1mL of culture media (DMEM, 10% FBS, 1% PS, 1× sodium pyruvate, 1× MEM NEA, and 1× HEPES). Single use aliquots were stored at −80C until use. Two days before transduction, 250,000 Ba/F3 cells were plated in 6 well-plates. Cells were transduced by replacing media with 20-250uL of concentrated virus, 0.4mL 10× polybrene (Sigma; TR-1003-G), and complete media without antibiotics (RPMI 1640, 10% FBS and 10ng/mL mouse IL3) to a final volume of 3.6mL. After 48h incubation with the virus, cells were expanded into 2mL of complete media supplemented with 0.7ug/mL puromycin and 10ng/mL purified human prolactin or mouse prolactin. After selection recovery, stable pools of cells were analyzed by flow cytometry using APC anti-Flag-tag antibody (Biolegend; 637307). Stable pools went through another round of selection in complete media supplemented with 0.7ug/mL puromycin and 1ng/mL human prolactin or purified mouse prolactin and were analyzed by flow cytometry. Highest expressing stable pools were used for the cell-based PRLR proliferation assay.

The cell-based PRLR proliferation assay was modified from elsewhere.^26^ 100uL of Ba/F3 cells stably expressing human or mouse PRLR were seeded in 96-well plates at 50,000 cells/well in RPMI media supplemented with 10% FBS, 1% PS, and 0.7ug/mL puromycin. The cells were incubated for 6h at 37C, 5% CO2. 100uL of 2× Fc-PRL fusions, human prolactin wild-type, human prolactin N59D mutant, or mouse prolactin wild-type serially diluted in PBS was added to the plate and incubated for 48h at 37C, 5% CO2. 20uL of WST-1 (Thomas Scientific; C755B06) or MTS (Abcam; ab197010) was added to each well and incubated at 37C, 5% CO2 for 1-4h until the desired color developed. The absorbance was measured at 450nm and 650nm for WST-1 and 490 for MTS. Prism (GraphPad Prism) was used to fit logistic curves to the data and generate Log(EC_50_) and Emax values. Reported data represent mean ± SEM of three replicates.

#### *In vitro* serum proteolysis study

20ug Fc-PRL fusions was diluted in 150uL of PBS. Samples were added to 150uL of human serum (Millipore Sigma, H6914-20ML) on ice. Samples were vortexed for 10s, and then a 3-5μL aliquot was taken for timepoint 0. Reactions were then incubated on a thermocycler at 37C. 3-5μL aliquots were taken at 30min, 60min, 3h, and 6h and stored at −20C. 4× Nupage LDS sample buffer (Thermo Fisher Scientific, NP0007) with 2.5% BME was added to all aliquots (diluted 1:5 in PBS) and boiled at 70C for 10min. The aliquots were then separated on Bis-Tris gels, 12% (Thermo Fisher Scientific; XP04205BOX) under reducing and denaturing conditions in MES buffer (Thermo Fisher Scientific; NP0002). Samples were transferred to a nitrocellulose membrane (Thermo Fisher; IB23002) using an iblot2 Dry Blotting System (Invitrogen, IB21001). Blots were then blocked in TBST (0.2% Tween) and 5% BSA for 1h. Blots were then incubated for 1h with HRP-conjugated anti-human IgG1 Fc (Invivogen; 31413) diluted 1:10000 in blocking buffer. Blots were washed 3× in TBST (0.2% Tween). Chemiluminescent bands were detected using SuperSignal West Femto Maximum Sensitivity Substrate (Thermo Fisher Scientific, 34095).

#### Pharmacokinetic studies in non-lactating mice

All animal work was approved by the Harvard Medical School IACUC under protocol IS00003310. The animal experiment was modified from elsewhere.^24^ On day 0, Tg276 mice (Jackson; 004919) or C57bl/6j mice (Jackson; 000664) were administered a 5mg/kg dose of Fc-PRL fusions, PRL N59D, or 5mg/kg IVIG (Creative Biomart, THP-0108) either by intravenous injection into the tail vein, subcutaneous injection, or oral gavage. Blood samples were collected from the tail vein at various time points.

The serum concentrations of the Fc-PRL fusions were determined using a quantitative ELISA as previously described. Anti-human PRL antibodies were coated onto MaxiSorp 96-well ELISA plates to capture Fc-PRL fusions from 50uL of serially diluted serum. HRP-conjugated anti-human IgG1 Fc antibodies were used for detection. A standard curve was generated by serially diluting purified Prolactin-XL into PBS. Prism (GraphPad Prism) was used to fit a sigmoidal curve to the data and used to quantify Prolactin-XL in the serum samples. Reported Prolactin-XL concentrations is represented as mean ± SEM.

The serum concentration of His-tagged PRL N59D was determined using a quantitative ELISA as follows: 50ul of 0.1ug/mL anti-human PRL antibodies (Abcam; ab244037) were diluted in PBS and used to coat MaxiSorp 96-well ELISA plates for 1h at room temperature. Plates were washed 3 times in PBST (PBS with 0.05% Tween 20, and then the plates were blocked with 200uL of blocking buffer (PBS with 3% BSA) overnight at 4C. Plates were again washed 3 times in PBST. 50uL of serum serially diluted in PBS was added to the plates and incubated at room temperature for 1h. Plates were then washed 5 times in PBST. 50uL of HRP-conjugated anti-6xHis (Abcam; 1187) diluted 1:5000 in blocking buffer was added to the plates and incubated at room temperature for 1h. Plates were then washed 5 times with PBST. 50uL of TMB Substrate Solution (Thermo Fisher Scientific; 34028) equilibrated to room temperature was added to the plates and incubated for 7 min or until desired color develops. Reactions were stopped by adding 50uL of 2M Sulfuric Acid to each well. The absorbance was measured at 450nm. A standard curve was generated by serially diluting purified PRL N59D into PBS. Prism (GraphPad Prism) was used to fit a sigmoidal curve to the data and used to quantify PRL N59D in the serum samples. Reported PRL N59D concentrations is represented as mean ± SEM.

The serum concentration of IVIG (Creative Biomart, THP-0108) was determined using a quantitative ELISA as follows: 50ul of 0.1ug/mL goat anti-human IgG Fc antibodies (Abcam; ab977221) were diluted in PBS and used to coat MaxiSorp 96-well ELISA plates for 1h at room temperature. Plates were washed 3 times in PBST (PBS with 0.05% Tween 20, and then the plates were blocked with 200uL of blocking buffer (PBS with 3% BSA) overnight at 4C. Plates were again washed 3 times in PBST. 50uL of serum serially diluted in PBS was added to the plates and incubated at room temperature for 1h. Plates were then washed 5 times in PBST. 50uL of HRP-conjugated goat anti-human IgG Fc (Invitrogen; 31413) diluted 1:5000 in blocking buffer was added to the plates and incubated at room temperature for 1h. Plates were then washed 5 times with PBST. 50uL of TMB Substrate Solution (Thermo Fisher Scientific; 34028) equilibrated to room temperature was added to the plates and incubated for 7 min or until desired color develops. Reactions were stopped by adding 50uL of 2M Sulfuric Acid to each well. The absorbance was measured at 450nm. A standard curve was generated by serially diluting IVIG into PBS. Prism (GraphPad Prism) was used to fit a sigmoidal curve to the data and used to quantify IVIG in the serum samples.

For Fc-PRL fusions, the half-life of the distribution and elimination phases were calculated by fitting two-phase decay curves to the data in PRISM (GraphPad Prism). A one-phase decay was fit to the PRL N59D data in PRISM and used to calculate the elimination half-life. AUC_inf_ and AUMC_inf_ was also calculated using PRISM.

Serum clearance was calculated as:

$$\text{CL}=\text{Dose}\times {\text{AUC}}_{\inf}^{-1}\text{.}$$

Volumes of distribution at steady stat were estimated as:

$$V_{ss}=\text{Dose}\times {\text{AUMC}}_{\inf}\times {\left({\text{AUC}}_{\inf}\right)}^{-2}\text{.}$$

#### Pharmacokinetic studies in lactating mice

All animal work was approved by the Harvard Medical School IACUC under protocol IS00003310. Tg276 mice (Jackson; 004919) or C57bl/6j mice (Jackson; 000664) were mated at 8 weeks of age. Dams that cannibalized pups were excluded. On day 7 post-partum, litters were normalized to 5 pups per dam. Pups were weighed daily until they were weaned at day 21 postpartum or they reached endpoint (20% weight loss compared to positive controls). The weight of the pups is reported as mean ± sem. PRISM (GraphPad Prism) was used to perform multiple unpaired t-tests or two-way ANOVA to compare pup weights.

Additionally, on day 7 post-partum dams were administered either a 5mg/kg dose of Prolactin-XL or a PBS control either by intravenous injection into the tail vein or by subcutaneous injection. Blood samples were collected from the tail vein at various time points. Serum concentrations of Prolactin-XL and pharmacokinetic parameters were determined as previously described.

#### Bromocriptine-inhibited galactopoiesis studies in mice

All animal work was approved by the Harvard Medical School IACUC under protocol IS00003310. The experiment was modified from elsewhere.^67^ C57bl/6j mice (Jackson; 000664) were mated at 8 weeks of age. Dams that cannibalized pups were excluded. On day 7 post-partum, litters were normalized to 5 pups per dam. Pups were weighed daily until they were weaned at day 21 postpartum or they reached endpoint (20% weight loss compared to positive controls). The weight of the pups is reported as mean ± sem. PRISM (GraphPad Prism) was used to perform two-way ANOVA with Bonferroni correction to compare the weight of the pups and to perform Kaplan-Meier analysis of the pup’s survival. The survival proportions are represented as a percentage +/− SE.

On day 7 post-partum dams were administered either a 0.05mg/kg, 0.5mg/kg, or 5mg/kg dose of Prolactin-XL, 5mg/kg dose of PRL N59D, or a PBS control by subcutaneous injection once on day 7 or every other dat. Additionally, beginning on day 7 post-partum dams were administered twice-daily a 200ug dose of Bromocriptine (2mg/mL bromocriptine mesylate (Sigma; 1076501) and 2mg/mL tartaric acid (Sigma; PHR1472) dissolved in PBS and 20% ethanol) or100uL of vehicle (2mg/mL tartaric acid (Sigma; PHR1472) dissolved in PBS with 20% ethanol). Dams were dosed until all their pup’s reached endpoint or were weaned at day 21 post-partum.

#### Stimulating galactopoiesis in mice with established milk production

All animal work was approved by the Harvard Medical School IACUC under protocol IS00003310. C57bl/6j mice (Jackson; 000664) were mated at 8 weeks of age. On day 7 postpartum, litters were normalized to 5 pups per dam. Pups were weighed daily until they were weaned at day 21 postpartum or they reached endpoint (20% weight loss compared to positive controls). Beginning on day 7 postpartum, and every other day thereafter, dams were administered 5mg/kg dose of Prolactin-XL or a PBS control by subcutaneous. Dams were dosed until their pups were weaned at day 21 postpartum. The weight of the pups is reported as mean ± sem. PRISM (GraphPad Prism) was used to perform multiple unpaired t-tests with Bonferroni correction to compare the weight of the pups.

#### Pharmacokinetics studies in pups

All animal work was approved by the Harvard Medical School IACUC under protocol IS00003310. C57bl/6j mice (Jackson; 000664) were mated at 8 weeks of age. On day 7 post-partum, litters were normalized to 5 pups per dam. Beginning on day 7 post-partum, and every other day thereafter, dams were administered a 5mg/kg dose of Prolactin-XL or a PBS control by subcutaneous injection (*n* = 3 per time point). Dams were dosed until their pups were sacrificed. Pups were weighed daily until they were sacrificed by CO2 asphyxiation and decapitation at various timepoints for blood collection (*n* = 15 per time point). Serum concentrations of Prolactin-XL was determined by a quantitative ELISA as previously described. Reported Prolactin-XL concentrations is represented as mean ± SEM.

#### Mammary gland harvesting in lactating mice

All animal work was approved by the Harvard Medical School IACUC under protocol IS00003310. C57bl/6j mice (Jackson; 000664) were mated at 8 weeks of age. On day 7 post-partum, litters were normalized to 5 pups per dam. Pups were weighed daily until their dams were sacrificed for mammary gland harvesting, at which point the pups were also sacrificed. Beginning on day 7 post-partum, and every other day thereafter, dams were administered a5mg/kg dose of Prolactin-XL or a PBS control by subcutaneous injection. Dams were dosed until they were sacrificed for mammary gland harvesting at various time points between day 7 post-partum and day 21 post-partum (*n* = 3 per time point). Blood was collected from the dams via cardiac puncture, and then cardiac perfusion was performed. After cardiac perfusion, the mammary glands were harvested. The inguinal mammary glands from one flank were harvested for whole carmine staining and the inguinal mammary glands from the other side were used to generate FFPE sections. The abdominal mammary gland from one side was harvested, stored in RNALater (Thermo Fisher, AM702) for 24h at 4C, and stored at −80C until further study. The proteins from the other abdominal mammary gland from was extracted as follows: The abdominal mammary glands were weighed and homogenized in RIPA Lysis buffer (Cell Signaling Technology; 9806) supplemented with cOmplete protease inhibitor cocktail (Sigma; 11836145001) and Phosphatase inhibitor cocktail (Thermo Fisher; A32957) according to the manufacturer’s instructions. The amount of protein in the supernatant was quantified via BCA (Thermo Fisher; 23227) according to the manufacturer’s instructions. Aliquots were stored at −20C until further use.

#### ELISA assay of Fc-PRL in mouse mammary glands

The concentrations of Prolactin-XL in the abdominal mammary glands were determined using a quantitative ELISA as previously described. Anti-human PRL antibodies (Abcam; ab244037) were coated onto MaxiSorp 96-well ELISA plates to capture Prolactin-XL from serially diluted mammary gland protein extracts. HRP-conjugated anti-human IgG1 Fc antibodies (Invivogen; 31413) were used for detection. A standard curve was generated by serially diluting purified Prolactin-XL into PBS. Prism (GraphPad) was used to fit a sigmoidal curve to the data and used to quantify Prolactin-XL in the protein extract samples. Reported Prolactin-XL concentrations is represented as mean ± SEM of biological triplicates.

#### Western blot analysis of lactation biomarkers in mouse mammary glands

20ug of abdominal mammary gland protein extracts were combined with equal parts (v/v) of 2× laemmli BME buffer (Bio Rad 1610737), boiled for 10min at 100C, and separated on 4–20% Tris-glycine gels (Thermo Fisher; XP04205BOX) in Tris-glycine SDS running buffer (Thermo Fisher; LC2675). The protein was then transferred onto nitrocellulose membrane (Thermo Fisher; IB23002) and blocker for 1h in TBST with 1% BSA. The primary antibodies were diluted in blocking buffer at 1:1000 anti-prolactin receptor (Abcam; ab214303), 1:1000 anti-β-casein (Thermo Fisher, MA1-46056), 1:1000 anti-STAT5 (Cell Signaling Technologies; 94205), 1:1000 anti-pSTAT5 (Cell Signaling Technologies; 9351S), 1:1000 anti-STAT3 (Cell Signaling Technologies; 12640), 1:1000 anti-pSTAT3 (Cell Signaling Technologies; 9145), and 1:10,000 anti-β-actin (Cell Signaling Technologies; 3700S). Secondary antibodies were diluted in blocking buffer at 1:10,000 anti-mouse-HRP (Thermo Fisher; 31340) and 1:10,000 anti-rabbit-HRP (Thermo Fisher; 31460). Chemiluminescence was detected using SuperSignal West Dura Extended Duration Substrate (Thermo Fisher; 34075). The densitometry of the western blots was analyzed by the Image Lab software. The data is reported as mean ± SEM of biological triplicates.

#### Western blot analysis of Fc-Prolactin from maternal or pup serum

10uL of serum from Prolactin-XL dosed dams or from pups fed by Prolactin-XL dosed dams were diluted 1:5 in PBS and 4× Nupage LDS sample buffer (Thermo Fisher Scientific, NP0007) with 2.5% BME. They were then boiled for 10min at 70C and separated on 12% Bis-Tris gels (Thermo Fisher Scientific; XP04205BOX) in MES running buffer (Therm0 Fisher Scientific; NP0002). The protein was then transferred onto nitrocellulose membrane (Thermo Fisher; IB23002) and blocker for 1h in TBST (0.2% Tween) with 5% BSA. Blots were then incubated for 1h with HRP-conjugated anti-human IgG1 Fc (Invivogen; 31413) diluted 1:10000 in blocking buffer. Blots were washed 3× in TBST (0.2% Tween). Chemiluminescent bands were detected using SuperSignal West Femto Maximum Sensitivity Substrate (Thermo Fisher Scientific, 34095).

#### H&E staining

Inguinal mammary glands were fixed in 10% neutral-buffered formalin for 24h. Hematoxylin and eosin (H&E) slides were prepared by the Rodent Histopathology core at Harvard Medical School. Dr. Roderick Bronson, a rodent histopathology from the Rodent Histopathology core at Harvard Medical School, reviewed all H&E slides. Imaging of the slides was provided by the Neurobiology Imaging Facility (NIF) at Harvard Medical School, Boston, MA.

### Quantification and statistical analysis

GraphPad Prism was used for all statistical analysis. Statistical details of experiments can be found in figure legends and STAR Methods section, including statistical tests used, n, and precision measures. Significance was defined as a *p*-value less than 0.5, with adjustment as necessary.

## Supplemental information

Document S1. Figures S1–S14 and Tables S1–S3 and S5–S12

Table S4. Fc-prolactin fusion variants

Table S13. Amino acid sequences used in the studies

## References

[1] Marasco L., West D. (2009).

[2] Moriwaki M., Welt C.K. (2021). PRL Mutation Causing Alactogenesis: Insights Into Prolactin Structure and Function Relationships. J. Clin. Endocrinol. Metab..

[3] Kauppila A., Chatelain P., Kirkinen P., Kivinen S., Ruokonen A. (1987). Isolated prolactin deficiency in a woman with puerperal alactogenesis. J. Clin. Endocrinol. Metab..

[4] Iwama S., Welt C.K., Romero C.J., Radovick S., Caturegli P. (2013). Isolated Prolactin Deficiency Associated With Serum Autoantibodies Against Prolactin-Secreting Cells. J. Clin. Endocrinol. Metab..

[5] Turkington R.W. (1972). Phenothiazine stimulation test for prolactin reserve: the syndrome of isolated prolactin deficiency. J. Clin. Endocrinol. Metab..

[6] Flagg J., Busch D.W. (2019). Utilizing a risk factor approach to identify potential breastfeeding problems. Glob. Pediatr. Health.

[7] Wambach K., Spencer B. (2019).

[8] Yoshida Y., Kawasaki Y., Morikawa N., Tanabe M., Satoh K., Tsukamoto T., Nakano M. (1991). [A kinetic study on serum prolactin concentration in the thyrotropin-releasing hormone test]. Kaku Igaku.

[9] Freeman M.E., Kanyicska B., Lerant A., Nagy G. (2000). Prolactin: structure, function, and regulation of secretion. Physiol. Rev..

[10] Watson C.J. (2022). Alveolar cells in the mammary gland: lineage commitment and cell death. Biochem. J..

[11] Martin P., Bateson P. (1982). The lactation–blocking drug bromocriptine and its application to studies of weaning and behavioral development. Dev. Psychobiol..

[12] Powe C.E., Allen M., Puopolo K.M., Merewood A., Worden S., Johnson L.C., Fleischman A., Welt C.K. (2010). Recombinant human prolactin for the treatment of lactation insufficiency. Clin. Endocrinol..

[13] Strohl W.R. (2015). Fusion Proteins for Half-Life Extension of Biologics as a Strategy to Make Biobetters. BioDrugs.

[14] Levin D., Golding B., Strome S.E., Sauna Z.E. (2015). Fc fusion as a platform technology: potential for modulating immunogenicity. Trends Biotechnol..

[15] Jafari R., Zolbanin N.M., Rafatpanah H., Majidi J., Kazemi T. (2017). Fc-fusion Proteins in Therapy: An Updated View. Curr. Med. Chem..

[16] Duivelshof B.L., Murisier A., Camperi J., Fekete S., Beck A., Guillarme D., D’Atri V. (2021). Therapeutic Fc-fusion proteins: Current analytical strategies. J. Sep. Sci..

[17] Elkins P.A., Christinger H.W., Sandowski Y., Sakal E., Gertler A., de Vos A.M., Kossiakoff A.A. (2000). Ternary complex between placental lactogen and the extracellular domain of the prolactin receptor. Nat. Struct. Biol..

[18] Svensson L.A., Bondensgaard K., Nørskov-Lauritsen L., Christensen L., Becker P., Andersen M.D., Maltesen M.J., Rand K.D., Breinholt J. (2008). Crystal Structure of a Prolactin Receptor Antagonist Bound to the Extracellular Domain of the Prolactin Receptor. J. Biol. Chem..

[19] Teilum K., Hoch J.C., Goffin V., Kinet S., Martial J.A., Kragelund B.B. (2005). Solution Structure of Human Prolactin. J. Mol. Biol..

[20] Ridgway J.B., Presta L.G., Carter P. (1996). “Knobs-into-holes” engineering of antibody CH3 domains for heavy chain heterodimerization. Protein Eng..

[21] Wei H., Cai H., Jin Y., Wang P., Zhang Q., Lin Y., Wang W., Cheng J., Zeng N., Xu T., Zhou A. (2017). Structural basis of a novel heterodimeric Fc for bispecific antibody production. Oncotarget.

[22] Von Kreudenstein T.S., Escobar-Carbrera E., Lario P.I., D’Angelo I., Brault K., Kelly J., Durocher Y., Baardsnes J., Woods R.J., Xie M.H. (2013). Improving biophysical properties of a bispecific antibody scaffold to aid developability. mAbs.

[23] Zwolak A., Leettola C.N., Tam S.H., Goulet D.R., Derebe M.G., Pardinas J.R., Zheng S., Decker R., Emmell E., Chiu M.L. (2017). Rapid Purification of Human Bispecific Antibodies via Selective Modulation of Protein A Binding. Sci. Rep..

[24] Lee C.-H., Kang T.H., Godon O., Watanabe M., Delidakis G., Gillis C.M., Sterlin D., Hardy D., Cogné M., Macdonald L.E. (2019). An engineered human Fc domain that behaves like a pH-toggle switch for ultra-long circulation persistence. Nat. Commun..

[25] Bailey M.J., Duehr J., Dulin H., Broecker F., Brown J.A., Arumemi F.O., Bermúdez González M.C., Leyva-Grado V.H., Evans M.J., Simon V. (2018). Human antibodies targeting Zika virus NS1 provide protection against disease in a mouse model. Nat. Commun..

[26] Jomain J.-B., Tallet E., Broutin I., Hoos S., van Agthoven J., Ducruix A., Kelly P.A., Kragelund B.B., England P., Goffin V. (2007). Structural and thermodynamic bases for the design of pure prolactin receptor antagonists: X-ray structure of Del1-9-G129R-hPRL. J. Biol. Chem..

[27] Can M., Guven B., Atmaca H., Acıkgoz S., Mungan G. (2011). Clinical characterization of patients with macroprolactinemia and monomeric hyperprolactinemia. Kaohsiung J. Med. Sci..

[28] Bruhns P., Iannascoli B., England P., Mancardi D.A., Fernandez N., Jorieux S., Daëron M. (2009). Specificity and affinity of human Fcγ receptors and their polymorphic variants for human IgG subclasses. Blood.

[29] Tao M.H., Morrison S.L. (1989). Studies of aglycosylated chimeric mouse-human IgG. Role of carbohydrate in the structure and effector functions mediated by the human IgG constant region. J. Immunol..

[30] Arduin E., Arora S., Bamert P.R., Kuiper T., Popp S., Geisse S., Grau R., Calzascia T., Zenke G., Kovarik J. (2015). Highly reduced binding to high and low affinity mouse Fc gamma receptors by L234A/L235A and N297A Fc mutations engineered into mouse IgG2a. Mol. Immunol..

[31] Lo M., Kim H.S., Tong R.K., Bainbridge T.W., Vernes J.-M., Zhang Y., Lin Y.L., Chung S., Dennis M.S., Zuchero Y.J.Y. (2017). Effector-attenuating Substitutions That Maintain Antibody Stability and Reduce Toxicity in Mice. J. Biol. Chem..

[32] Jasion V.S., Burnett B.P. (2015). Survival and digestibility of orally-administered immunoglobulin preparations containing IgG through the gastrointestinal tract in humans. Nutr. J..

[33] Valente D., Mauriac C., Schmidt T., Focken I., Beninga J., Mackness B., Qiu H., Vicat P., Kandira A., Radošević K. (2020). Pharmacokinetics of novel Fc-engineered monoclonal and multispecific antibodies in cynomolgus monkeys and humanized FcRn transgenic mouse models. mAbs.

[34] Tustian A.D., Endicott C., Adams B., Mattila J., Bak H. (2016). Development of purification processes for fully human bispecific antibodies based upon modification of protein A binding avidity. mAbs.

[35] Kochenour N.K. (1980). Lactation suppression. Clin. Obstet. Gynecol..

[36] Watson C.J. (2006). Key stages in mammary gland development - Involution: apoptosis and tissue remodelling that convert the mammary gland from milk factory to a quiescent organ. Breast Cancer Res..

[37] Hong L., Wang Z., Wei X., Shi J., Li C. (2020). Antibodies against polyethylene glycol in human blood: A literature review. J. Pharmacol. Toxicol. Methods.

[38] Zhu J., Garrigues L., Van den Toorn H., Stahl B., Heck A.J.R. (2019). Discovery and Quantification of Nonhuman Proteins in Human Milk. J. Proteome Res..

[39] Ben-Jonathan N., LaPensee C.R., LaPensee E.W. (2008). What Can We Learn from Rodents about Prolactin in Humans?. Endocr. Rev..

[40] Egli M., Leeners B., Kruger T.H.C. (2010). Prolactin secretion patterns: basic mechanisms and clinical implications for reproduction. Reproduction.

[41] Weaver S.R., Hernandez L.L. (2016). Autocrine-paracrine regulation of the mammary gland. J. Dairy Sci..

[42] Ben-Jonathan N., Liby K., McFarland M., Zinger M. (2002). Prolactin as an autocrine/paracrine growth factor in human cancer. Trends Endocrinol. Metab..

[43] Smith G.H. (2016). Binuclear Cells in the Lactating Mammary Gland: New Insights on an Old Concept?. J. Mammary Gland Biol. Neoplasia.

[44] Rios A.C., Fu N.Y., Jamieson P.R., Pal B., Whitehead L., Nicholas K.R., Lindeman G.J., Visvader J.E. (2016). Essential role for a novel population of binucleated mammary epithelial cells in lactation. Nat. Commun..

[45] Lopez Vicchi F., Becu-Villalobos D. (2017). Prolactin: The Bright and the Dark Side. Endocrinology.

[46] Tritos N.A., Klibanski A. (2019).

[47] Roelfsema F., Pijl H., Keenan D.M., Veldhuis J.D. (2012). Prolactin Secretion in Healthy Adults Is Determined by Gender, Age and Body Mass Index. PLoS One.

[48] Tay C.C., Glasier A.F., McNeilly A.S. (1996). Twenty-four hour patterns of prolactin secretion during lactation and the relationship to suckling and the resumption of fertility hi breast-feeding women. Hum. Reprod..

[49] Al-Chalabi M., Bass A.N., Alsalman I. (2021). StatPearls.

[50] Cox D.B., Owens R.A., Hartmann P.E. (1996). Blood and milk prolactin and the rate of milk synthesis in women. Exp. Physiol..

[51] Beck K.L., Weber D., Phinney B.S., Smilowitz J.T., Hinde K., Lönnerdal B., Korf I., Lemay D.G. (2015). Comparative Proteomics of Human and Macaque Milk Reveals Species-Specific Nutrition during Postnatal Development. J. Proteome Res..

[52] Petkova S.B., Akilesh S., Sproule T.J., Christianson G.J., Al Khabbaz H., Brown A.C., Presta L.G., Meng Y.G., Roopenian D.C. (2006). Enhanced half-life of genetically engineered human IgG1 antibodies in a humanized FcRn mouse model: potential application in humorally mediated autoimmune disease. Int. Immunol..

[53] Pyzik M., Kozicky L.K., Gandhi A.K., Blumberg R.S. (2023). The therapeutic age of the neonatal Fc receptor. Nat. Rev. Immunol..

[54] Cianga P., Medesan C., Richardson J.A., Ghetie V., Ward E.S. (1999). Identification and function of neonatal Fc receptor in mammary gland of lactating mice. Eur. J. Immunol..

[55] Morales F.C., Hayashi Y., van Pelt C.S., Georgescu M.-M. (2012). NHERF1/EBP50 controls lactation by establishing basal membrane polarity complexes with prolactin receptor. Cell Death Dis..

[56] Xu R., Nelson C.M., Muschler J.L., Veiseh M., Vonderhaar B.K., Bissell M.J. (2009). Sustained activation of STAT5 is essential for chromatin remodeling and maintenance of mammary-specific function. J. Cell Biol..

[57] Ueda E.K., Huang K., Nguyen V., Ferreira M., Andre S., Walker A.M. (2011). Distribution of prolactin receptors suggests an intraductal role for prolactin in the mouse and human mammary gland, a finding supported by analysis of signaling in polarized monolayer cultures. Cell Tissue Res..

[58] Keeler C., Jablonski E.M., Albert Y.B., Taylor B.D., Myszka D.G., Clevenger C.V., Hodsdon M.E. (2007). The kinetics of binding human prolactin, but not growth hormone, to the prolactin receptor vary over a physiologic pH range. Biochemistry.

[59] Sachs H.C., Committee On Drugs (2013). The transfer of drugs and therapeutics into human breast milk: an update on selected topics. Pediatrics.

[60] Lawrence R.A. (2022). Breastfeeding.

[61] Madlon-Kay D.J. (1986). “Witch’s Milk”: Galactorrhea in the Newborn. Am. J. Dis. Child..

[62] Buehring G.C. (1982). Short communication. Witch’s milk: potential for neonatal diagnosis. Pediatr. Res..

[63] Hassiotou F., Hepworth A.R., Metzger P., Tat Lai C., Trengove N., Hartmann P.E., Filgueira L. (2013). Maternal and infant infections stimulate a rapid leukocyte response in breastmilk. Clin. Transl. Immunology.

[64] Lollivier V., Guinard-Flament J., Ollivier-Bousquet M., Marnet P.-G. (2002). Oxytocin and milk removal: two important sources of variation in milk production and milk quality during and between milkings. Reprod. Nutr. Dev..

[65] Muranishi Y., Parry L., Averous J., Terrisse A., Maurin A.-C., Chaveroux C., Mesclon F., Carraro V., Bruhat A., Fafournoux P., Jousse C. (2016). Method for collecting mouse milk without exogenous oxytocin stimulation. Biotechniques.

[66] Weström B., Arévalo Sureda E., Pierzynowska K., Pierzynowski S.G., Pérez-Cano F.-J. (2020). The Immature Gut Barrier and Its Importance in Establishing Immunity in Newborn Mammals. Front. Immunol..

[67] Knight C.H., Calvert D.T., Flint D.J. (1986). Inhibitory effects of bromocriptine on mammary development and function in lactating mice. J. Endocrinol..

[68] Ventrella D., Ashkenazi N., Elmi A., Allegaert K., Aniballi C., DeLise A., Devine P.J., Smits A., Steiner L., Forni M. (2021). Animal Models for In Vivo Lactation Studies: Anatomy, Physiology and Milk Compositions in the Most Used Non-Clinical Species: A Contribution from the ConcePTION Project. Animals..

[69] Caron A., Palin M.F., Hovey R.C., Cohen J., Laforest J.P., Farmer C. (2020). Effects of sustained hyperprolactinemia in late gestation on mammary development of gilts. Domest. Anim. Endocrinol..

[70] Mathews A.T., Banks C.M., Trott J.F., Sainz R.D., Farmer C., Pendergast I.I., Hovey R.C. (2021). Metoclopramide induces preparturient, low-level hyperprolactinemia to increase milk production in primiparous sows. Domest. Anim. Endocrinol..

[71] Palin M.-F., Caron A., Farmer C. (2023). Effects of sustained hyperprolactinemia in late gestation on the mammary parenchymal tissue transcriptome of gilts. BMC Genom..

[72] Farmer C., Mathews A.T., Hovey R.C. (2019). Using domperidone to induce and sustain hyperprolactinemia in late-pregnant gilts. Domest. Anim. Endocrinol..

[73] Page-Wilson G., Smith P.C., Welt C.K. (2007). Short-term prolactin administration causes expressible galactorrhea but does not affect bone turnover: pilot data for a new lactation agent. Int. Breastfeed. J..

[74] Wada Y., Suyama F., Sasaki A., Saito J., Shimizu Y., Amari S., Ito Y., Sago H. (2019). Effects of domperidone in increasing milk production in mothers with insufficient lactation for infants in the neonatal intensive care unit. Breastfeed. Med.

[75] Kauppila A., Arvela P., Koivisto M., Kivinen S., Ylikorkala O., Pelkonen O. (1983). Metoclopramide and breast feeding: transfer into milk and the newborn. Eur. J. Clin. Pharmacol..

[76] Gómez-Gallego C., Ilo T., Jaakkola U., Salminen S., Periago M.J., Ros G., Frias R. (2014). A Method to Collect High Volumes of Milk from Mice (Mus Musculus). An. Vet. Murcia.

[77] Page-Wilson G., Smith P.C., Welt C.K. (2006). Prolactin suppresses GnRH but not TSH secretion. Horm. Res..

[78] Brown R.S.E., Wyatt A.K., Herbison R.E., Knowles P.J., Ladyman S.R., Binart N., Banks W.A., Grattan D.R. (2016). Prolactin transport into mouse brain is independent of prolactin receptor. FASEB J..

[79] Alekseev N.P. (2022).

[80] Smiley K.O., Brown R.S.E., Grattan D.R. (2022). Prolactin action is necessary for parental behavior in male mice. J. Neurosci..

[81] Georgescu T., Khant Aung Z., Grattan D.R., Brown R.S.E. (2022). Prolactin-mediated restraint of maternal aggression in lactation. Proc. Natl. Acad. Sci. USA.

[82] Nandi S. (1958). Endocrine control of mammarygland development and function in the C3H/He Crgl mouse. J. Natl. Cancer Inst..

[83] Tien J., Leonoudakis D., Petrova R., Trinh V., Taura T., Sengupta D., Jo L., Sho A., Yun Y., Doan E. (2023). Modifying antibody-FcRn interactions to increase the transport of antibodies through the blood-brain barrier. mAbs.

[84] Zhang Y., Pardridge W.M. (2001). Mediated efflux of IgG molecules from brain to blood across the blood-brain barrier. J. Neuroimmunol..

[85] Garg A., Balthasar J.P. (2009). Investigation of the influence of FcRn on the distribution of IgG to the brain. AAPS J..

[86] Abuqayyas L., Balthasar J.P. (2013). Investigation of the role of FcγR and FcRn in mAb distribution to the brain. Mol. Pharm..

[87] The Human Protein Atlas. https://www.proteinatlas.org/.

[88] Karlsson M., Zhang C., Méar L., Zhong W., Digre A., Katona B., Sjöstedt E., Butler L., Odeberg J., Dusart P. (2021). A single-cell type transcriptomics map of human tissues. Sci. Adv..

[89] Powe C.E., Puopolo K.M., Newburg D.S., Lönnerdal B., Chen C., Allen M., Merewood A., Worden S., Welt C.K. (2011). Effects of recombinant human prolactin on breast milk composition. Pediatrics.

[90] Lin-Cereghino J., Wong W.W., Xiong S., Giang W., Luong L.T., Vu J., Johnson S.D., Lin-Cereghino G.P. (2005). Condensed protocol for competent cell preparation and transformation of the methylotrophic yeast Pichia pastoris. Biotechniques.

