# Supplementary material for: A long-lasting prolactin stimulates galactopoiesis in mice
Source: iScience. 2025 Jul 15;28(8):113112. doi: 10.1016/j.isci.2025.113112 (PMC12337787; doi:10.1016/j.isci.2025.113112)
Supplement: Document S1. Figures S1–S14 and Tables S1–S3 and S5–S12 [file mmc1.pdf]

iScience, Volume *28*

Supplemental information

A long-lasting prolactin stimulates galactopoiesis in mice

Kasia Kready, Kailyn E. Doiron, Katherine Redfield Chan, Jeffrey C. Way, Quincey Justman, Camille E. Powe, and Pamela A. Silver

Supporting Information

Fig. S1. Reducing and Non-reducing SDS-PAGE gels of purified Fc-Prolactin Variants.

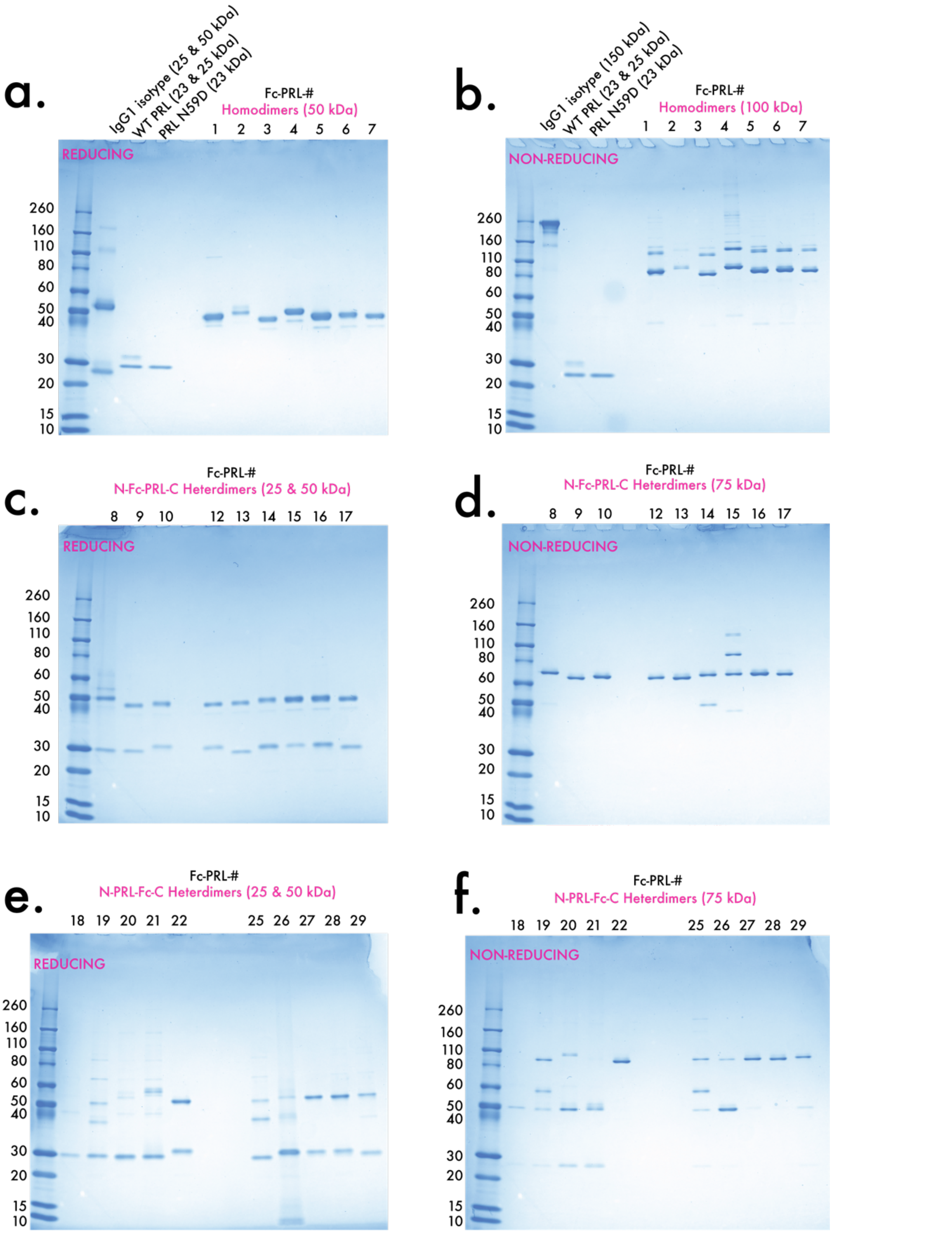

Supplemental Figure 1: Reducing and Non-reducing SDS-PAGE gels of purified Fc-Prolactin variants. The proteins were expressed in HEK 293F cells by transient transfection and purified by Protein A (Fc-PRL- 1, 3, 5-7, 9-17, 19, 22-29) or His-Tag (Fc-PRL- 2, 4, 8, 18, 20-21). 250nM of each fusion protein was analyzed by reducing (**a**, **c**, and **e**) and non-reducing (**b**, **d**, and

f) 4-20% Tris glycine SDS-Page gel. Molecular weights were verified by reducing SDS-PAGE gels and/or western blots (data not shown).

Fig. S2. Fc-Prolactin variants with differential expression titers.

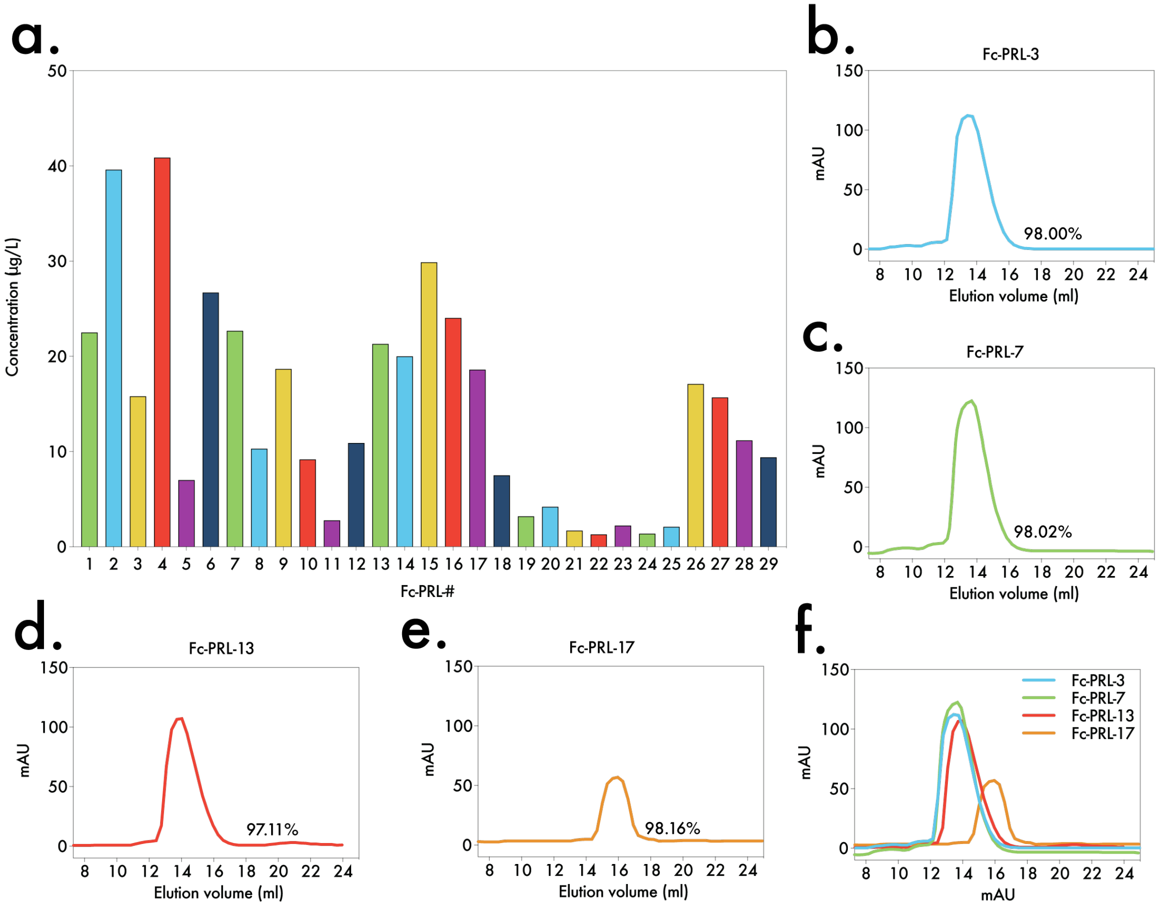

Supplemental Figure 2: Fc-Prolactin variants with differential expression titers were **a** expressed transiently in Hek293F cells and purified either by Protein A (Fc-PRL- 1, 3, 5-7, 9-17, 19, 22-29) or His-Tag (Fc-PRL- 2, 4, 8, 18, 20-21). Molecular weights were verified by reducing SDS-PAGE gels and/or western blots (data not shown). Protein concentration was determined by BCA. **b-e** FPLC analysis of top 4 Fc-prolactin fusions using size-exclusion chromatography columns was performed to determine purity and aggregation. The SEC prolife (**b** Fc-PRL-3, **c** Fc-PRL-7, **d** Fc-PRL-13, **e** Fc-PRL-17, and **f** overlay) and the abundance (percentage) is presented for the different fusions.

Fig. S3. Production of high titer Prolactin-XL.

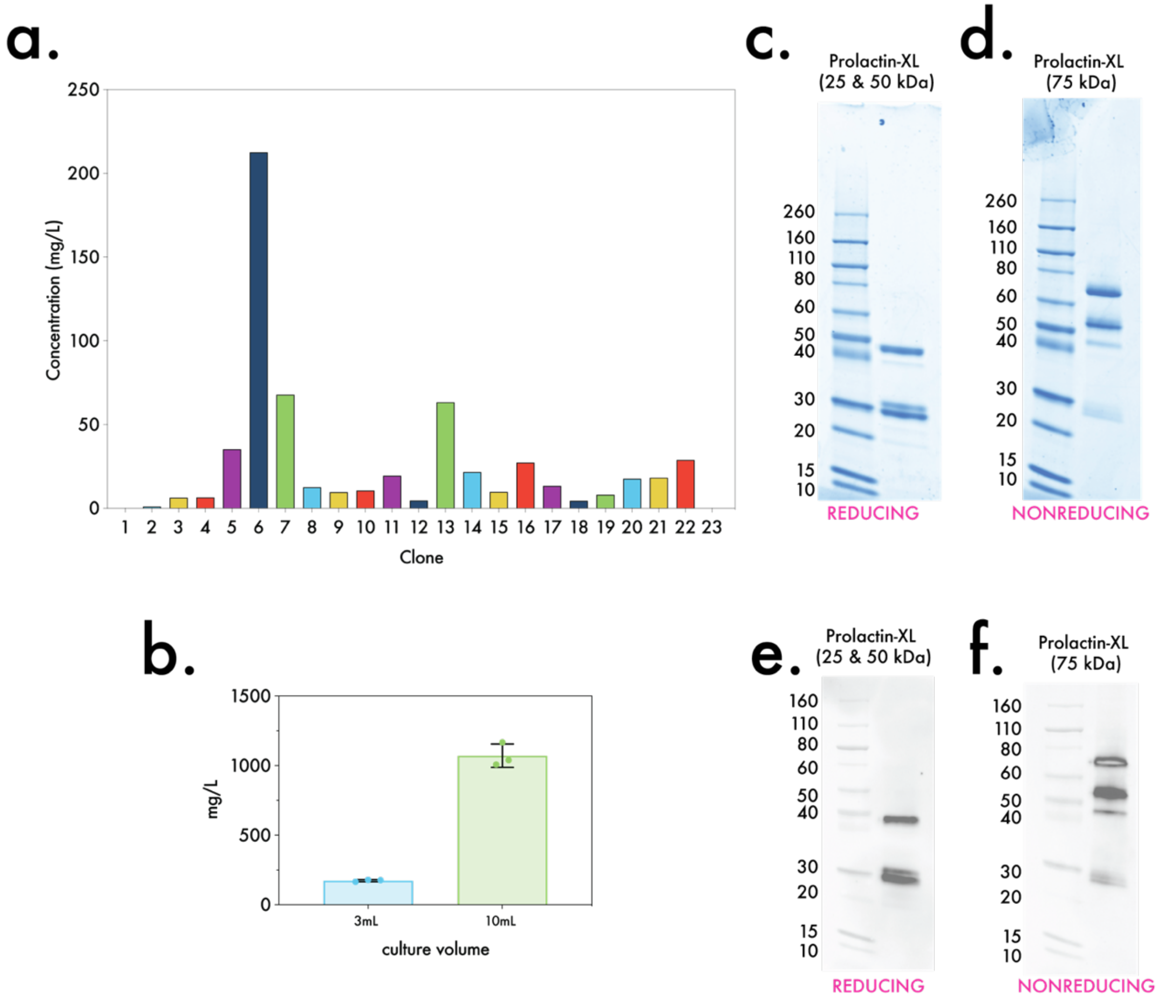

Supplemental Figure 3: Production of high titer Prolactin-XL. a *Pichia pastoris* clones of Fc-PRL-13, called Prolactin-XL, were grown in 3mL scout cultures of and expression titers were determined via ELISA (i.e., capture prolactin and detect Fc). Clone 6 was identified as the highest expressing clone and was used to produce protein for *in vivo* studies. **b** Clone 6 was grown in 3ml and 10mL cultures and expression titers were determined via ELISA. Data is depicted as mean ± SEM. **c-f** Prolactin-XL was purified via Protein A purification, and its molecular weight (expected 25 and 50 kDa) was verified via running 10ug on reducing 12% Bis Tris SDS-PAGE (**c**) and Western Blot (**e**). 10ug were also run on non-reducing 12% Bis Tris SDS-PAGE (**d**) and anti-human IgG1 Fc Western blots (**f**) to analyze overproduction of the Fc only monomer (expected molecular weight 50 or 25 kDa).

Fig. S4. Dose response curves demonstrating bioactivity of Fc-prolactin variants via human PRLR signaling.

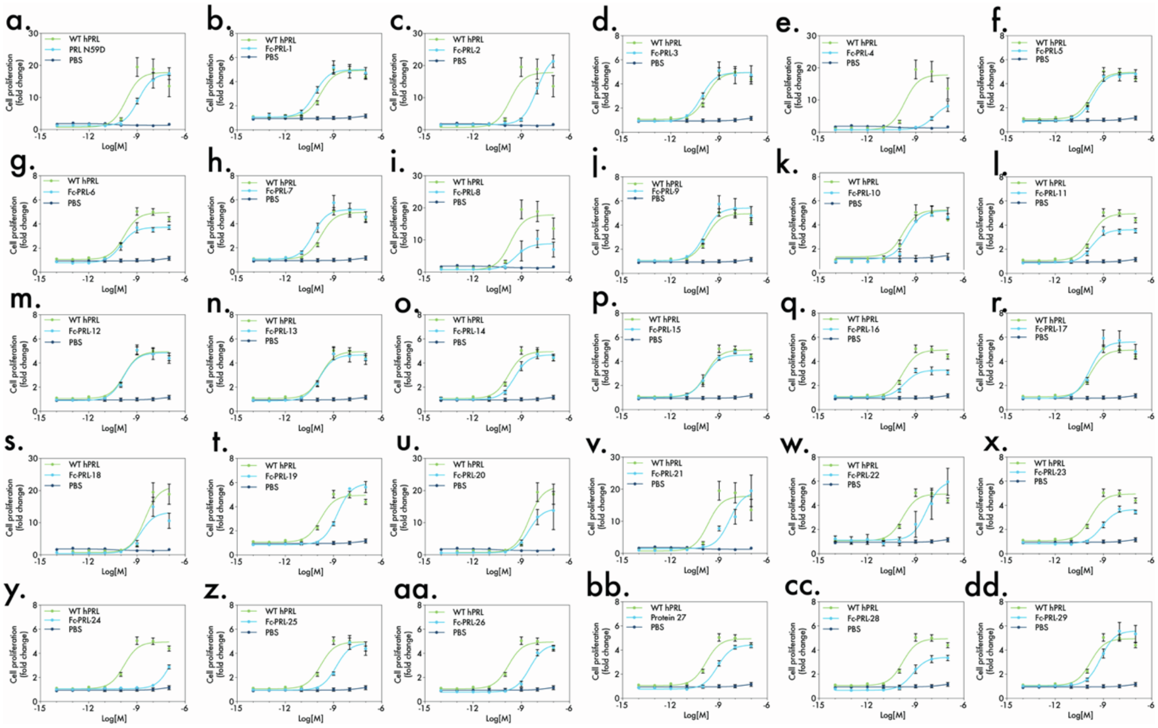

Supplemental Figure 4: Dose response curves demonstrating bioactivity of Fc-prolactin variants via human PRLR signaling. Fc-prolactin variants were assayed in an *in vitro* cell-based signaling assay previously described [14]. WT human His-tagged Prolactin was used as a positive control. PRISM was used to fit a non-linear curve and calculate Log(EC50) and Emax (Extended data table 3). Data is depicted as mean ± SEM.

Fig. S5. Dose response curves demonstrating bioactivity of Fc-Prolactin variants via mouse PRLR signaling.

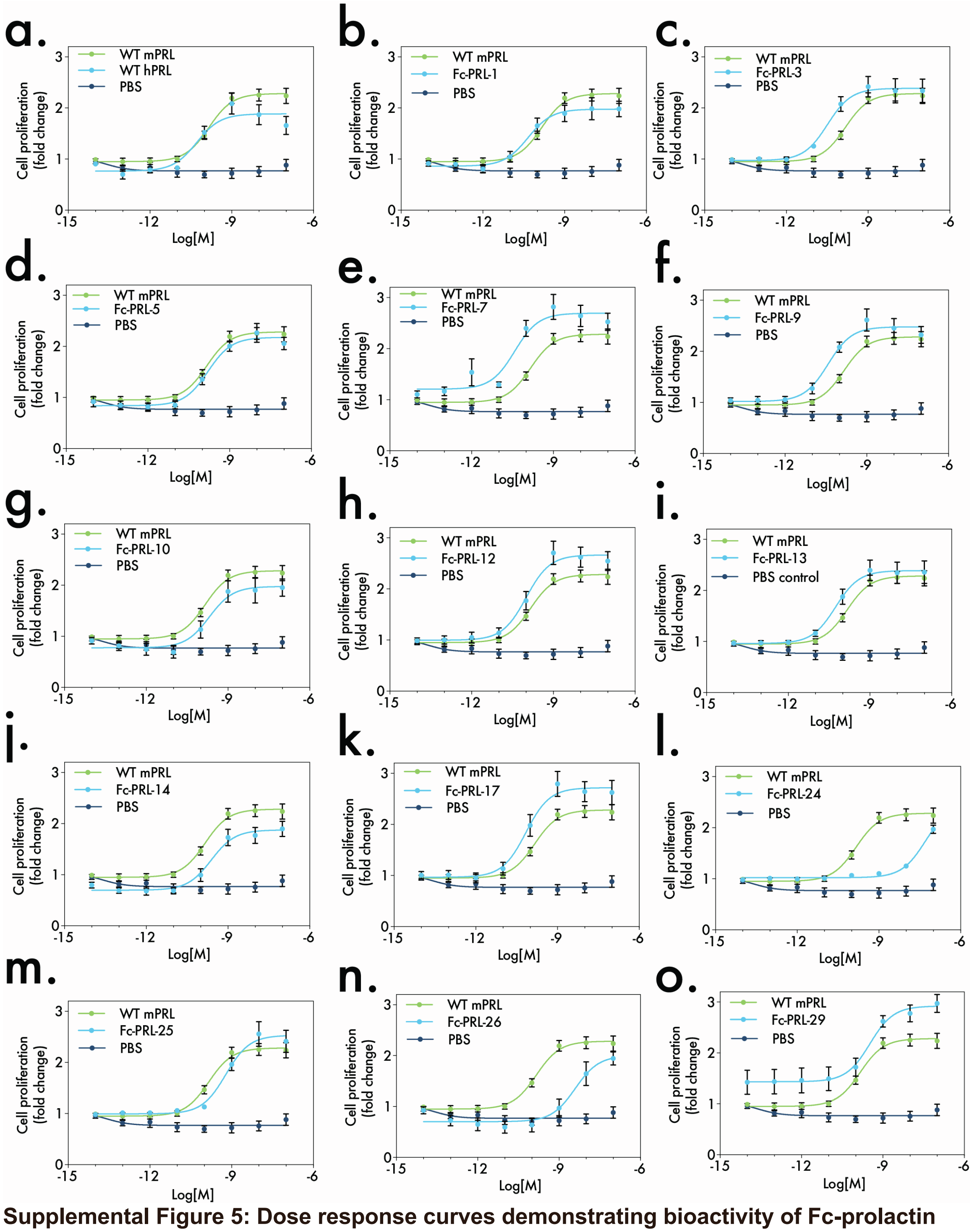

Supplemental Figure 5: Dose response curves demonstrating bioactivity of Fc-prolactin variants via mouse PRLR signaling. Fc-prolactin variants were assayed in an *in vitro* cell-based signaling assay previously described [14]. WT mouse His-tagged Prolactin was used as a positive control. PRISM was used to fit a non-linear curve and calculate Log(EC50) and Emax (Extended data table 5). Data is depicted as mean ± SEM.

Fig. S6. Binding of human Fc-prolactin variants to human or mouse FcRn by ELISA.

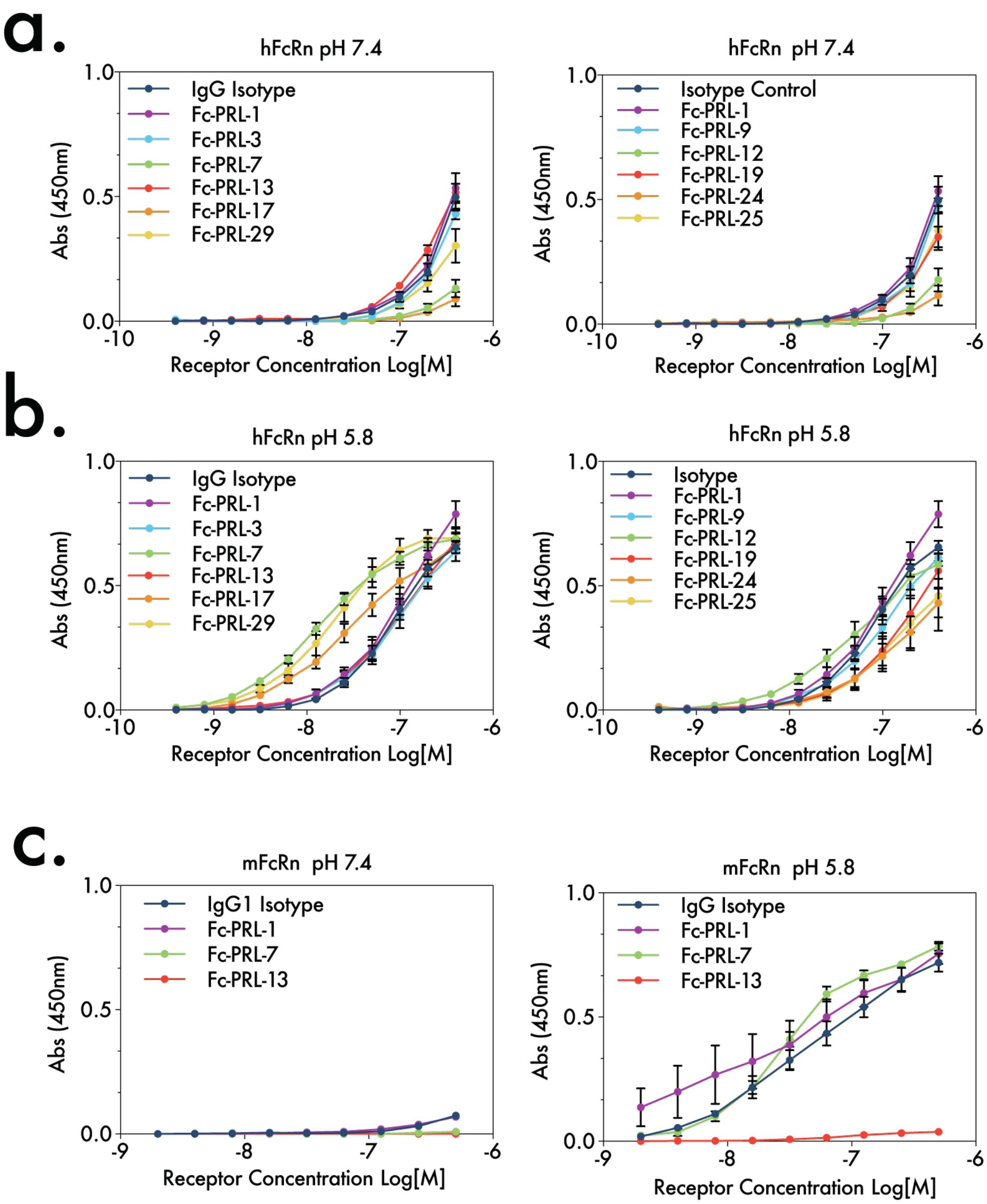

Supplemental Figure 6: Binding of human Fc-prolactin variants to human or mouse FcRn by ELISA. The binding of the fusions to human FcRn (**a-b**) or mouse FcRn (**c**) was measured via ELISA. For positive controls, we used IgG isotype control and Protein 1, which is a glycosylated Fc fused to PRL (N59D). Data is depicted at mean ± SEM triplicates except Fc-PRL-7 (n=2 for mouse FcRn), Fc-PRL-24 (n=2 for human FcRn), and Fc-PRL-29 (n=2 for human FcRn).

Fig. S7. Binding of human Fc-prolactin variants to human or mouse Fc receptors by ELISA.

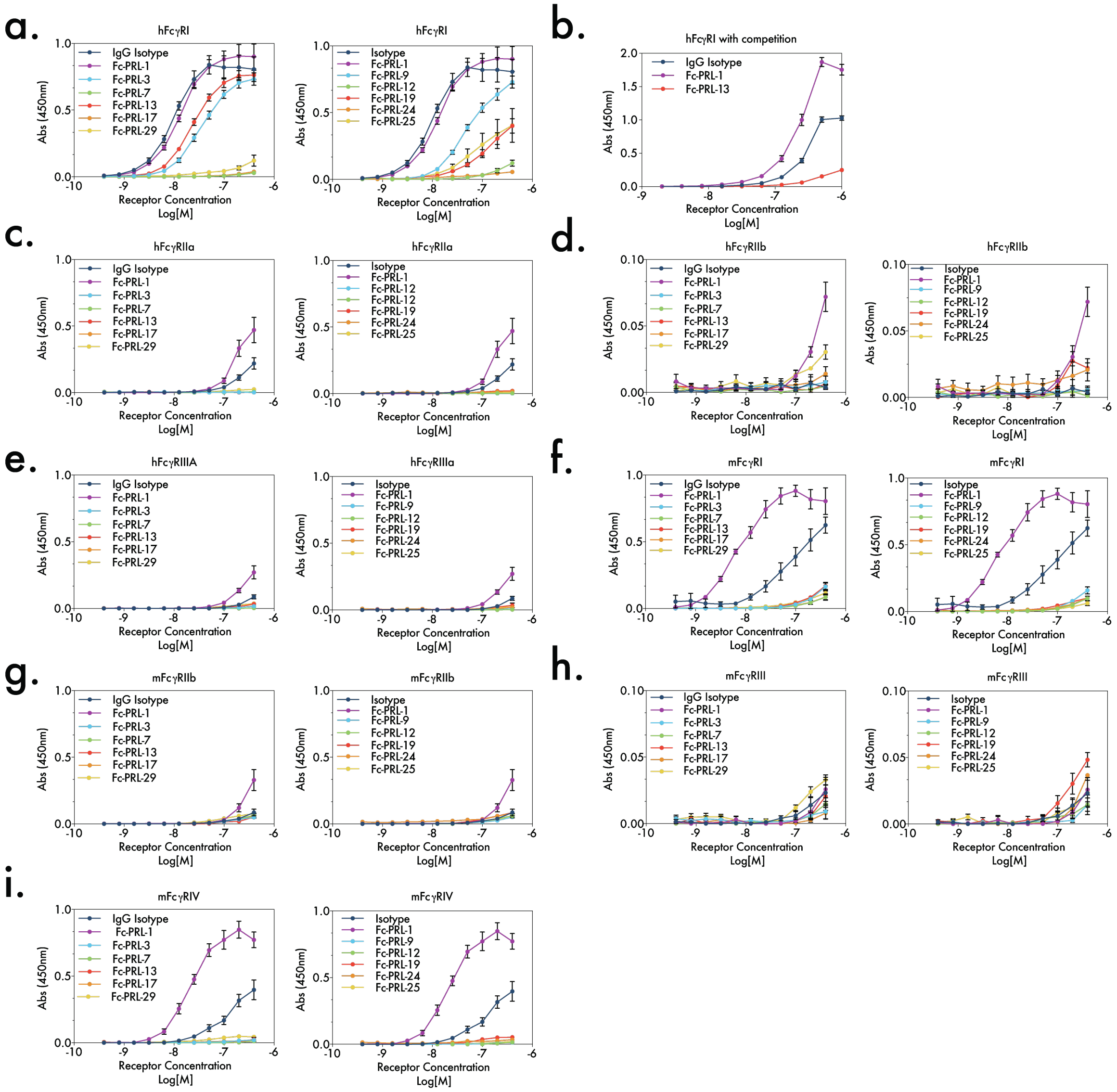

Supplemental Figure 7: Binding of human Fc-prolactin variants to human or mouse Fc receptors by ELISA. The binding of the fusions to hFcɣRI (**a**), hFcɣRI with competing IgG1 isotype control (**b**), hFcɣRIIa (**c**), hFcɣRIIb (**d**), hFcɣRIIIa (**e**), mFcɣRI (**f**), mFcɣRIIb (**g**), mFcɣRIII (**i**), and mFcɣRIV (**i**) was measured via ELISA. For positive controls, we used IgG isotype control and Protein 1, which is a glycosylated Fc fused to PRL (N59D). Data is depicted at mean ± SEM triplicates except Fc-PRL-24 (n=2 for human receptors and n=1 for mouse receptors) and Fc-PRL-29 (n=2 for human and mouse receptors).

Fig. S8. Gastrointestinal protease degradation of Fc-prolactin variants by SDS-PAGE gels.

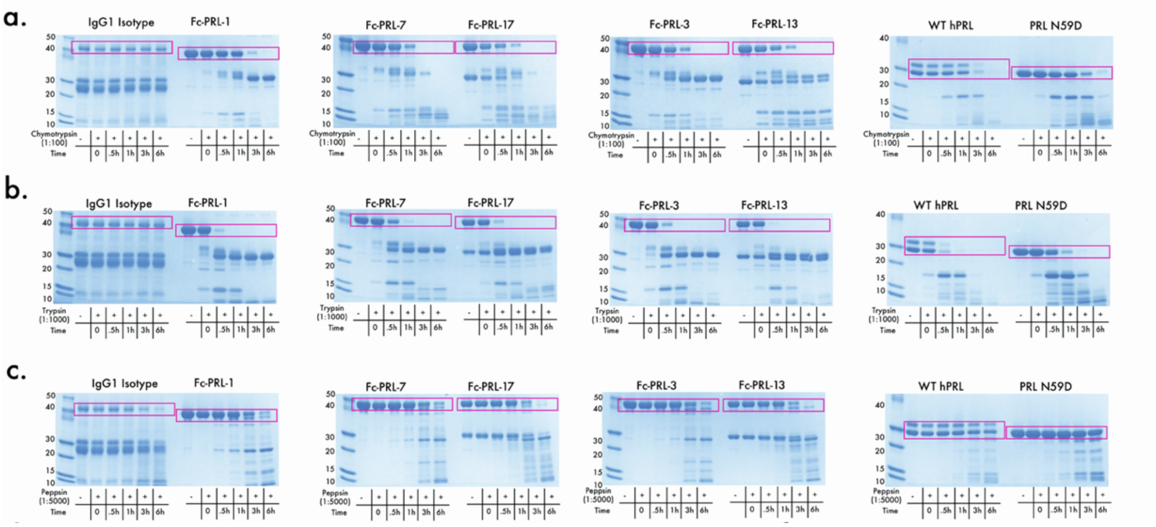

Supplemental Figure 8: Gastrointestinal protease degradation of Fc-prolactin variants by SDS-PAGE gels. Fc-prolactin variants were incubated with GI proteases trypsin (**b**), chymotrypsin (**a**), and pepsin (**c**) at 1:1000, 1:100, and 1:5000 and 37C, 25C, and 37C respectively. Aliquots were taken from the reaction at different time points, and the percent of the fusion remaining in each aliquot was measured by SDS-PAGE (**a-c**)). For positive controls, we used IgG isotype control and Protein 1, which is a glycosylated Fc fused to PRL (N59D). The arrows indicate bands that were used to calculate the percent remaining for each fusion on representative gels.

Fig. S9. Percent of Fc-prolactin variants remaining after gastrointestinal protease degradation.

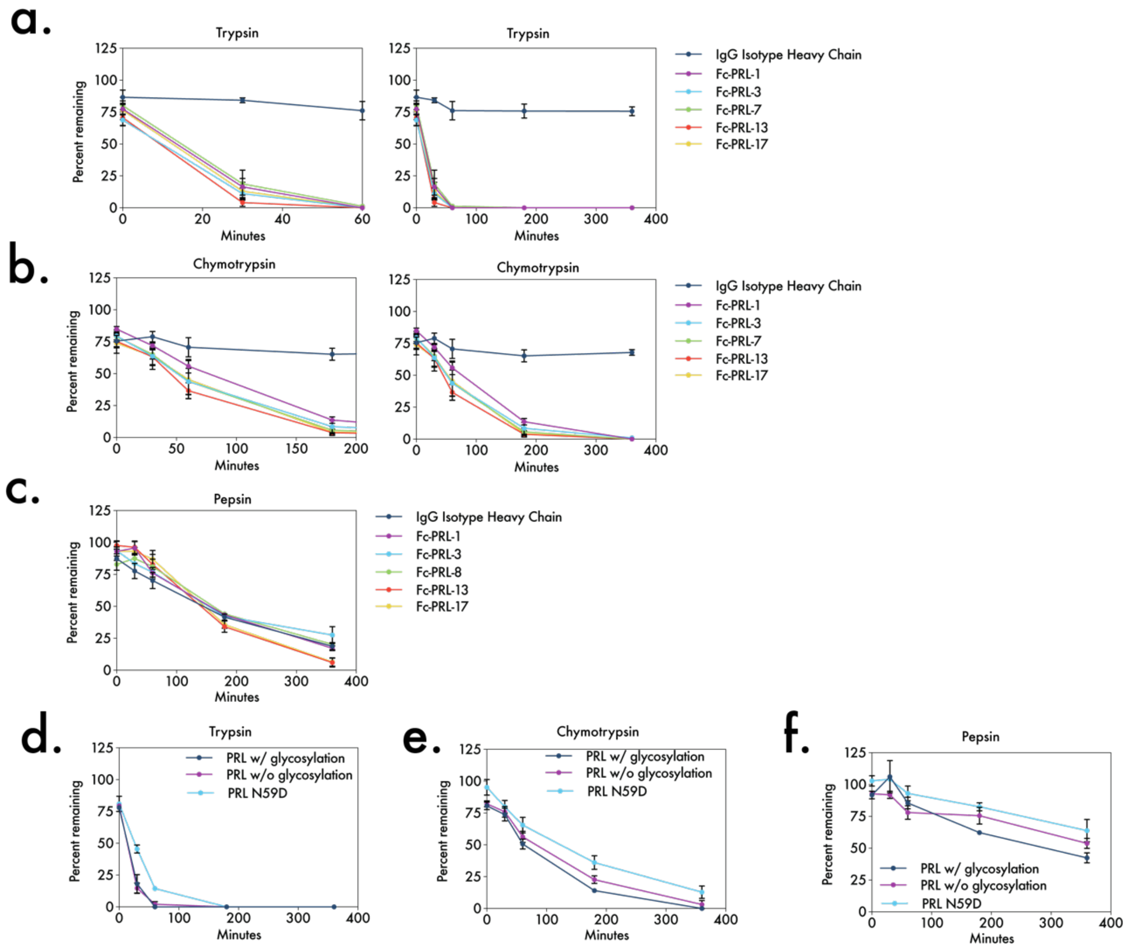

Supplemental Figure 9: Percent of Fc-prolactin variants remaining after gastrointestinal protease degradation. Fc-prolactin variants were incubated with GI proteases trypsin, chymotrypsin, and pepsin at 1:1000, 1:100, and 1:5000 and 37C, 25C, and 37C respectively. Aliquots were taken from the reaction at different time points, and the percent of the fusion remaining in each aliquot was measured by SDS-PAGE (Supplemental Figure 8) and densitometry (**a-f**). For positive controls, we used IgG isotype control and Protein 1, which is a glycosylated Fc fused to PRL (N59D). Experiments were conducted in triplicate. Data is depicted as mean ± SEM of triplicates.

Fig. S10. *In vitro* serum protease degradation of Prolactin-XL.

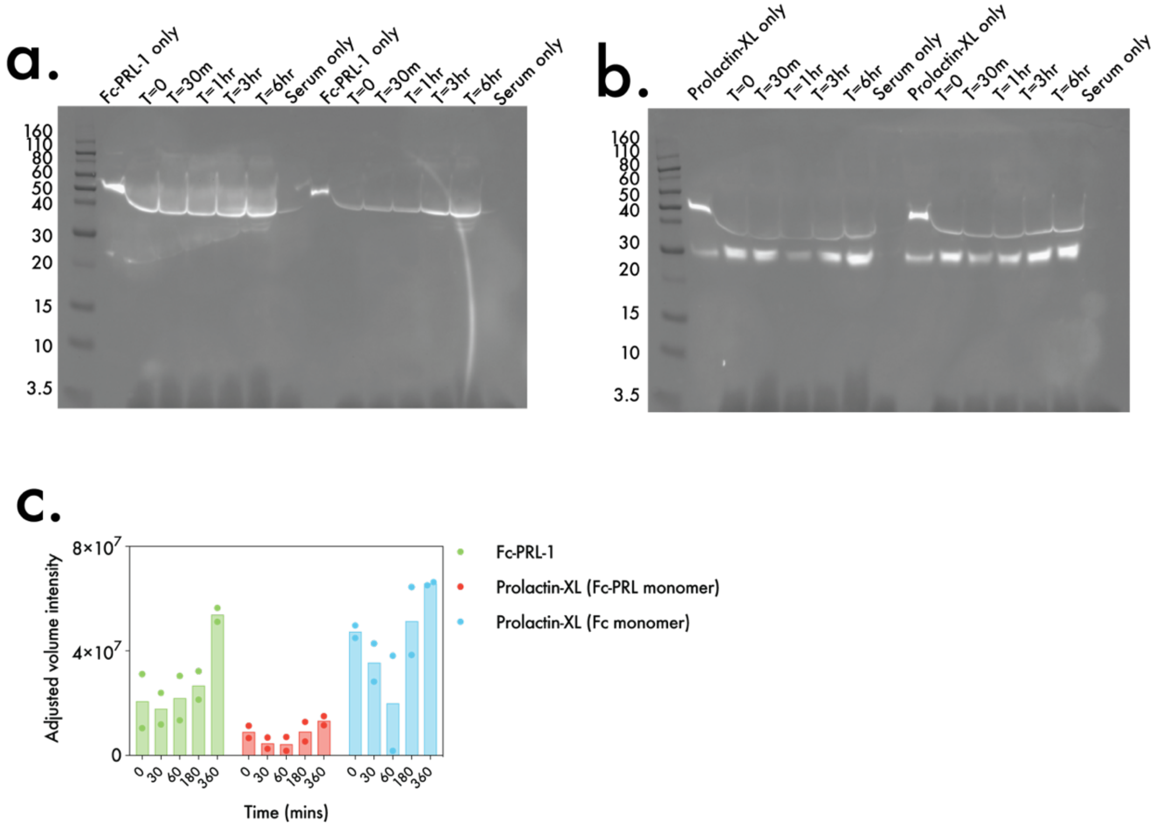

Supplemental Figure 10: *In vitro* serum protease degradation of Prolactin-XL. Prolactin-XL and Fc-PRL-1 were incubated with serum diluted 1:2 at 37C. Aliquots were taken from the reaction at different time points, and the percent of the fusion remaining in each aliquot was measured by anti-human IgG1 Fc Western Blot (**a-b**) and densitometry (**c**). For positive controls, we used Fc-PRL-1, which is a glycosylated Fc fused to PRL (N59D). Experiments were conducted in duplicate. Data is depicted as mean (**c**).

Fig. S11. Pharmacokinetic profiles of top 4 Fc-prolactin variants in Tg276 mice.

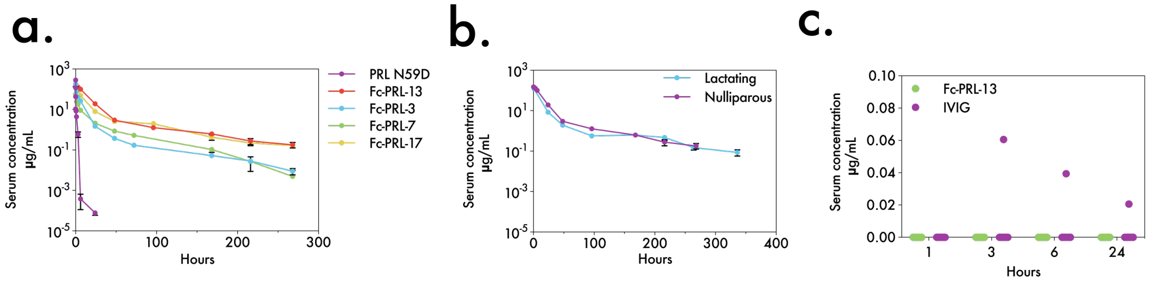

d.

PK parameters of top 4 performing Fc-prolactin variants in Tg276 mice

Protein	N	AUC_{inf} (ug days/mL)	Clearance (mL/day)	Beta-phase $T_{1/2}$ (h)	V_{ss} (mL/Kg)
Nulliparous					
I.V. PRL N59D	5	24	4.2	.0027*	2.3
I.V. Fc-PRL-3	4	649.1	0.15	49.3	1.2
I.V. Fc-PRL-7	4	311.2	0.32	35.1	7.0
I.V. Fc-PRL-13	4	2305	0.04	70.9	0.39
Oral Fc-PRL-13	8	0	0	0	0
I.V. Fc-PRL-17	4	1416	0.07	40.2	0.88
Lactating					
I.V. Fc-PRL-13	3	1742	0.057	167.2	0.90

* Data fit using one-phase decay, all other data was fit using two-phase decay

Supplemental Figure 11: Pharmacokinetic profiles of top 4 Fc-Prolactin variants in Tg276 mice. **a** Nulliparous Tg276 mice were injected with 5mg/kg I.V. of Fc-prolactin fusions (n=4) and PRL N59D (n=5). Blood was collected by tail nick post injection, and the concentration of the fusions in serum was measured by ELISA. The data are depicted as mean ± SEM. PRISM was used to fit either a one-phase decay (PRL N59D) or a two-phase decay (Fc-prolactin fusions). The Pharmacokinetic parameters are listed in the Table in **d. b** On the 7th day postpartum, the litters of lactating Tg276 mice were normalized to n=5, and the dams (n=3) were injected with 5mg/kg I.V. of Prolactin-XL (Fc-PRL-13). Blood was collected by tail nick post injection, and the concentration of the fusion in serum was measured by ELISA. The data are depicted as mean ± SEM. PRISM was used to fit a one-phase decay a two-phase decay. The Pharmacokinetic parameters are listed in the Table in **d. c** Nulliparous Tg276 mice were administered 5mg/kg of Fc-PRL-13, IVIG, or PRL N59D fusions (n=8) via oral gavage. Blood was collected by tail nick post injection, and the concentration of the proteins in serum was measured by ELISA. PRL N59D was undetectable (<50ng/mL), and Fc-PRL-13 was undetectable (<100ng/mL). The data for Fc-PRL-13 and IVIG are depicted as individual data points. Only 1/8 mice dosed with IVIG had detectable levels, whereas IVIG was undetectable in 7/8 mice.

Fig. S12. Western blots of Fc-PRL-13 in mouse maternal serum.

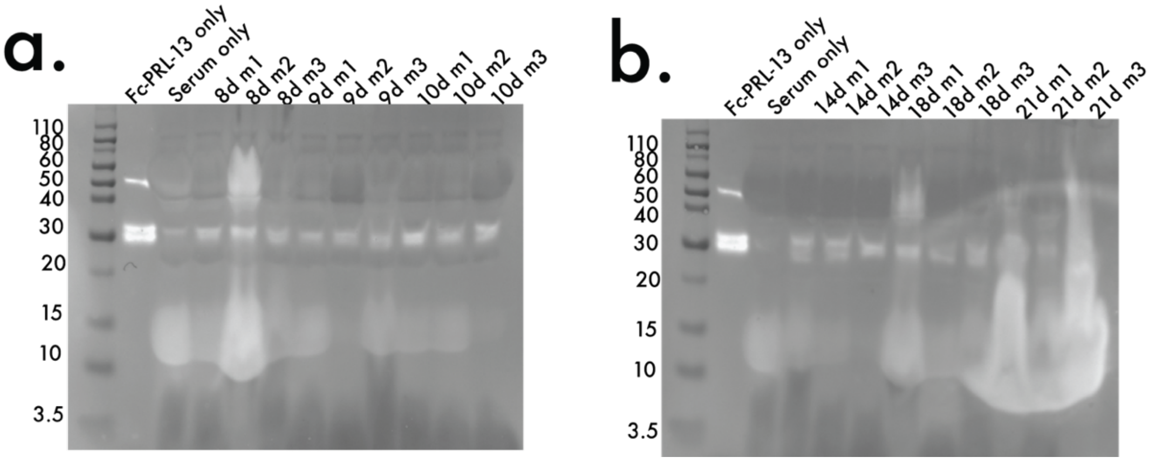

Supplemental Figure 12: Western blots of Fc-PRL-13 in mouse maternal serum. Lactating C57bl/6j mice (litters normalized to n=5) were administered S.C. 5mg/kg of Fc-PRL-13 every other day. Mice were sacrificed at 6 different time points (n=3), and blood was collected by cardiac puncture. The molecular weight of Fc-PRL-13 (expected 25 & 50 kDa) in serum (diluted 1:5) was measured by an anti-human IgG1 Fc Western Blot.

Fig. S13. Western blots of Fc-PRL-13 in serum from pups fed by Fc-PRL-13-dosed dams.

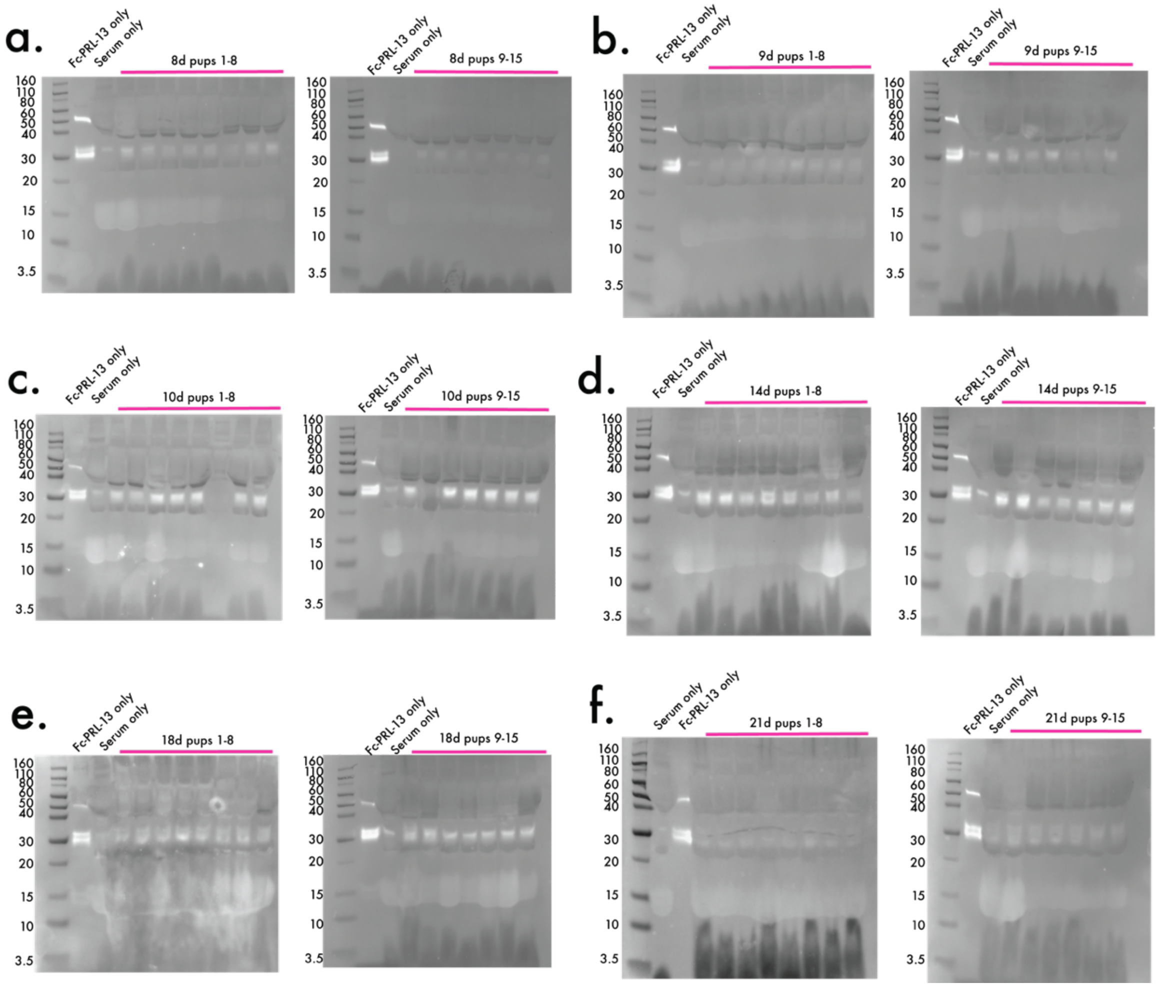

Supplemental Figure 13: Western blots of Fc-PRL-13 in serum from pups fed by Fc-PRL-13-dosed dams. Lactating C57bl/6j mice (litters normalized to n=5) were administered S.C. 5mg/kg of Fc-PRL-13 every other day. The pups of the dams repeatedly dosed were sacrificed (n=15), and their blood was collected by decapitation. The molecular weight of Fc-PRL-13 (expected 25 & 50 kDa) in serum (diluted 1:5) was measured by an ant-human IgG1 Fc Western Blot.

Fig. S14. Biomarker analysis of mammary glands.

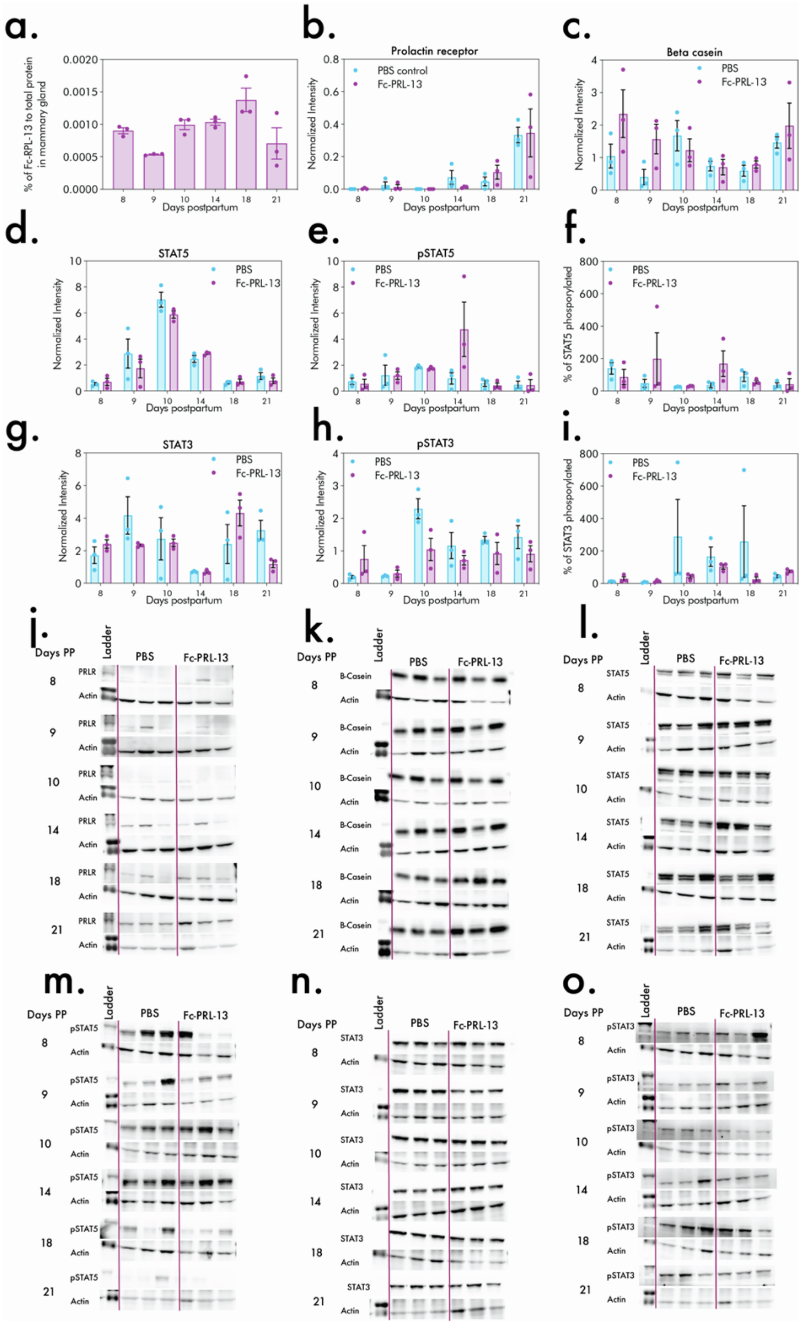

Supplemental Figure 14: Biomarker analysis of mammary glands. Lactating C57bl/6j mice were administered S.C. 5mg/kg of Fc-PRL-13 every other day. Mice were sacrificed at 6 different time points (n=3). Their abdominal mammary glands were collected, homogenized in RIPA buffer with protease and phosphatase inhibitors, and stored at -20C. **a** The concentration of Fc-PRL-13 in serum was measured by ELISA. The data are depicted as mean ± SEM. Expression of prolactin receptor (**b** and **j**), β-casein (**c** and **k**), STAT5 (**d** and **l**), pSTAT5 (**e** and **m**), STAT3 (**g** and **n**), and pSTAT3 (**h** and **o**) were analyzed by western blot and measured by densitometry. Intensity was normalized to an β-actin loading control. The data are depicted as mean ± SEM. The percent of phosphorylated STAT5 to total STAT5 and the percent of phosphorylated STAT3 to total STAT3 is depicted in **f** and **i**, respectively. Multiple unpaired t-tests with Bonferroni correction for multiple comparison were used to calculate the statistical. All comparisons were not statistically significant (alpha = 0.05).

Table S1. Functional descriptions of relevant human endogenous molecules impacting the pharmacokinetics of Fc-Prolactin fusions.

Table S1: Functional descriptions for relevant human endogenous molecules impacting the pharmacokinetics of Fc-Prolactin fusions

**Prolactin Variants**	
**Monomeric**	The biologically active form of prolactin is a 23 kDa monomer that signals via prolactin receptor (PRLR). The circulating pool of prolactin in serum is estimated to consist of 60-90% of monomeric prolactin [15,16]. The percentage of monomeric prolactin has been shown to increase in pregnant and lactating individuals [16]. The x-ray crystal structure of PRL in complex with prolactin receptor (PDB ID 3NPZ) shows that PRL has two asymmetric binding sites that contact the same site on two different PRLRs with different affinities. One face of PRL, the 'strong side', binds to PRLR with nanomolar affinity, whereas the opposing 'weak side' of PRL binds with micromolar affinity [17]. The strong side of PRL likely initiates binding to PRLR. Then when another PRLR binds to the weak side of PRL, the complete signaling complex is formed. The complete PRL:$(PRLR)_2$ signaling complex then locks into a conformation that is amenable to intracellular JAK2 dimerization and downstream STAT5 signaling. The PRL:$(PRLR)_2$ complex signals via the JAK2/STAT5 pathway that leads to upregulation of proliferative and anti-apoptotic factors essential for lactocyte survival [18]. PRL is then degraded via receptor-mediated degradation or pinocytosis. PRL is also found in milk and is likely transported via receptor-mediated transcytosis across lactocytes [19,20].
**Glycosylated Monomer**	The glycosylated monomer of prolactin is inactive because the glycosylation sterically hinders PRLR dimerization. The circulating pool of prolactin in serum is estimated to consist of 5-70% of glycosylated monomeric prolactin in nulliparous individuals [16,21]. The percentage of glycosylated monomeric prolactin was found to be lower in pregnant and lactating individuals [16].
**Dimeric**	The dimeric form of prolactin is inactive, and the circulating pool of serum prolactin is estimated to consist of 15-30% of prolactin dimers [15].
**Phosphorylated**	Prolactin can be phosphorylated on serine 179 by protein kinase A, casein, and p21-activated kinase 2 in secretory granules in the lactotrophs of the pituitary [22]. It has been shown in rats the percentage of phosphorylated prolactin changes during the estrous cycle and pregnancy [22]. It remains an open question how phosphorylated prolactin levels change in humans during menstruation, pregnancy, and lactation. It has yet to be determined whether phosphorylated prolactin signals via PRLR or inhibits PRLR signaling.
**Cleaved Prolactins**	Prolactin can be cleaved by Cathepsin D or metalloproteases between serine 155 and leucine 156 creating a 16 kDa product called, vasohibin[23]. Vasohibin is antiangiogenic because it inhibits PRLR on endothelial cells[23]. Other cleaved variants of prolactin (22 kDa and 14 kDa cleavage) have been found[15].
**Macroprolactin**	Macroprolactins are large molecular mass complexes (>150kDa) of prolactin and IgG or IgA autoantibodies. Macroprolactin is inactive and increases the half-life of prolactin. The circulating pool of prolactin is estimated to be 0-8% macroprolactin in nulliparous individuals [15]. Macroprolactin levels can increase in patients with hyperprolactinemia, and macroprolactin can be 15-60% of a patient's circulating prolactin pool [24]. Prolactin, IgG, and IgA are found in milk. In theory, macroprolactin can be transported into milk via either FcRn or PRLR. Macroprolactin differs from classical anti-drug antibodies because it does not increase prolactin clearance or induce hypersensitivity. Instead, macroprolactin functions in a paradigm like small molecule binding to human serum albumin, where the bound and unbound fractions of the small molecule are important parameters.
**Prolactin Receptor Isoforms**	

**Long**	The main isoform of PRLR is 598 amino acid protein, referred to as long PRLR [23]. It is the only isoform expressed in the mammary gland and drives milk production. PRLR binds to prolactin, placental lactogen, and growth hormone creating competition for the receptor. PRLR is also responsible for receptor-mediated degradation, and it is likely responsible for transport of prolactin into milk.
**∇S1**	This isoform has a truncated extracellular domain but intact intracellular domain. The effect of prolactin's binding to this isoform is unknown. This isoform may act as a ligand or receptor traps [23].
**I**	This isoform has a truncated intracellular domain but intact extracellular domain. Prolactin can bind to this isoform, but the effects on downstream signaling are still being elucidated [23].
**S1a and S1b**	These isoforms lack the intracellular domain and appears incapable of signaling. Prolactin can still bind the extracellular domains, and they may act as ligand or receptor traps [23].
**bp**	This isoform consists of only the extracellular domain, and it is soluble. Prolactin can still bind to this extracellular domain, and it may act as a ligand or receptor traps [23].
**Molecules binding to IgG1 Fc domain**	
**FcRn**	Neonatal Fc Receptor (FcRn) rescues IgG antibodies from lysosomal degradation by binding to IgG in a pH-dependent manner. FcRn binds to IgG at a low pH in acidic endosomes but does not bind to IgG at neutral pH on the cell surface [25]. In theory, FcRn can also rescue macroprolactin complexes from lysosomal degradation.
**FcɣRI**	FcɣRI has a high affinity for monomeric IgG and can activate immune effector functions [25].
**FcɣRIIa**	FcɣRIIa has a low affinity for monomeric IgG, requires IgG complexes to activate immune effector functions, and has two alleles [25]. Binding to FcɣRIIa contributes to receptor-mediated degradation.
**FcɣRIIb**	FcɣRIIb has a low affinity for monomeric IgG, requires IgG complexes to inhibit immune effector functions, and has two alleles and two isoforms [25]. Binding to FcɣRIIb contributes to receptor-mediated degradation.
**FcɣRIIc**	FcɣRIIc has a low affinity for monomeric IgG, requires IgG complexes to inhibit immune effector functions, and has two alleles [25]. Binding to FcɣRIIc contributes to receptor-mediated degradation.
**FcɣRIIIa**	FcɣRIIa has a low affinity for monomeric IgG, requires IgG complexes to activate immune effector functions, and has two alleles [25]. Binding to FcɣRIIIa contributes to receptor-mediated degradation.
**FcɣRIIIb**	FcɣRIIIb has a low affinity for monomeric IgG, requires IgG complexes to activate immune effector functions, and has one allele [25]. Binding to FcɣRIIIb contributes to receptor-mediated degradation.
**TRIM21**	TRIM21 has a low affinity for monomeric IgG and activates immune effector functions [25]. Binding to TRIM21 contributes to receptor-mediated degradation.
**FcRL5**	FcRL5 is a newly identified receptor with low affinity for monomeric IgG [25]. Its immune effector functions are still being elucidated. Binding to FcRL5 contributes to receptor-mediated degradation.
**C1q complexes**	IgG bound to antigens can bind to C1q complexes to activate the classical complement pathway.

Table S2. List of mutations used in Fc-Prolactin fusions.

Table S2: List of mutations used in Fc-Prolactin fusions

Mutations	Rationale
**Prolactin N59D**	A fraction of human prolactin made in mammalian cells is N-linked glycosylated, and glycosylation inactivates prolactin receptor-mediated signaling. However, not all prolactin homologs are glycosylated. Bovine, mouse, rat, water buffalo, and yak prolactin lack a N-linked glycosylation motif, and they instead have a threonine or aspartic acid at that position. We chose to use mutate human prolactin N59 to aspartic acid to remove the glycosylation site.
**Prolactin C191S, C199S**	Prolactin C191 and C199 form a disulfide bond at the c-termini. Our rationale for removing it was to create a flexible linker in N-PRL-Fc-C fusions between prolactin and Fc. However, 3 related individuals were identified with an early truncation of prolactin that deleted the C-terminal disulfide bond and resulted in prolactin deficiency and lactation insufficiency [26].
**Fc C220S**	IgG1 Fc C220 is in the hinge region of Fc and forms a disulfide bond with another Fc monomer. We removed this disulfide bond with a C220S mutations so that the hinge region of Fc can act more like a flexible linker in N-Fc-PRL-C fusions.
**Fc N297D**	A IgG1 Fc domain has one N-linked glycosylation site that primarily increases binding to Fc receptors. The N297D mutation removes this glycosylation.
**Fc K447A**	The IgG1 Fc K447A mutation is thought to decrease serum proteolysis of Fc fusions.
**Fc L234A, L235A ("LALA")**	Fc "LALA" mutations in monoclonal IgG antibodies decrease binding to Fc receptors and C1q, but they still have some residual antibody-dependent cell-mediated cytotoxicity and complement dependent cytotoxicity *in vitro* [8,9,27].
**Fc L234A, L235A, P329G ("LALAPG")**	Fc "LALAPG" mutations in monoclonal IgG antibodies knockout Fc receptor and C1q binding and activation of antibody-dependent cell-mediated cytotoxicity and complement dependent cytotoxicity [9,28].
**Fc M252Y, S254T, T256E ("YTE")**	Fc "YTE" mutations in monoclonal IgG antibodies enhances the serum half-life by increasing binding to FcRn at a low pH and decreasing binding to FcRn at a neutral pH. Fc "YTE" mutations in monoclonal IgG antibodies also decrease Fc Receptor binding [6].
**Fc V264E, L309D, Q311H, N434S ("EDHS")**	Fc "EDHS" mutations in monoclonal IgG antibodies enhances the serum half-life by increasing binding to FcRn at a low pH and decreasing binding to FcRn at a neutral pH. "EDHS" mutations in monoclonal antibodies have longer half-life than "YTE" mutants. Fc "EDHS" mutations in monoclonal IgG antibodies also decrease Fc Receptor binding [6].
**Fc H435R, Y436F ("RF")**	Fc "RF" mutations in monoclonal IgG antibodies were shown to decrease binding to recombinant Protein A without interfering with human FcRn binding. "RF" mutations should have even less binding at high concentration to an engineered Protein A commercially available as Mab Select SuRe [12].
**Fc Knob (T366W) / Fc Hole (T366S, L368A, Y407V) ("Knobs-in-Hole")**	Fc "Knobs-in-Hole" mutations allow you to engineer Fc heterodimers [1,2].

**Fc A (T350V, L351Y, F405A, Y407V) / Fc B (T350V, T366L, K293L, T394W) ("Fc A/ Fc B")**	Fc "Fc A/ Fc B" mutations allow you to engineer Fc heterodimers [3].

Table S3. Design schemes used in Fc-Prolactin fusions.

Table S3: Design schemes used in Fc-Prolactin fusions

Design Scheme	Rationale
**N-PRL-Fc-C fusions**	N-terminal amino acids 1-9 of prolactin bind to prolactin receptor, and deletion of these amino acids decreases binding and signaling [17]. A N-PRL-Fc-C fusion may maintain prolactin receptor signaling compared to a N-Fc-PRL-C fusions because prolactin's first 9 amino acids are free and may have less steric hinderance.
**N-Fc-PRL-C fusions**	In general, N-Fc-X-C fusions express better than N-X-Fc-C fusions. A N-Fc-PRL-C fusion may express better than a N-PRL-Fc-C fusion. The C-terminal amino acids of Prolactin are close to Prolactin Receptor in crystal structures of the ligand-receptor complex and may be important for receptor binding and prolactin secretion (PDB: 3NPZ).
**Homodimer fusions**	Fc fusions are naturally homodimeric, but dimeric prolactin is biologically inactive. A homodimeric Fc-prolactin fusion may have decreased prolactin receptor signaling compared to a heterodimeric fusion.
**Heterodimer fusions**	Heterodimeric Fc fusions allow you to modulate the valency of the fusion, meaning up to 4 proteins can be fused to a heterodimeric Fc. Because biologically active prolactin is monomeric, a heterodimer fusion allows you to engineer a Fc heterodimer fused to a single prolactin.
**GGsGG Linker between prolactin and Fc**	A flexible linker between prolactin and Fc may prevent either molecule from sterically interfering with it binding its on-target receptors.
**Use of half-life extension variants**	The two main clearance mechanisms for a Fc-prolactin fusion are prolactin-receptor mediated degradation and secretion into breastmilk. In theory, both clearance mechanisms could increase as the efficacy of the biologic increases. Prolactin receptor and FcRn are often co-expressed on the same cell types (endothelial cells, mammary gland cells, and certain lymphocytes). Predicting the dynamics of a Fc-Prolactin's PRLR-mediated degradation or FcRn-mediated recycling on these cells is not straightforward. Prolactin receptor binds prolactin at the cell surface (neutral pH) and releases prolactin in acidic endosomes (acidic pH). Conversely, FcRn binds Fc in acidic endosomes and releases it at the cell surface (neutral pH). We know that increasing binding to FcRn at *both* acidic and neutral pHs increases lysosomal degradation of IgG antibodies [6]. In theory, a Fc-PRL fusion could have increased lysosomal degradation in cells co-expressing prolactin receptor and FcRn because it binds receptors at the cell surface and in the endosomes. This becomes even more complex as you consider competition for binding of both prolactin receptor and FcRn. We decided to test half-life extension variants such as "YTE" or "EDHS", but we were not sure if they would increase or decrease the serum half-life. These mutations were tested in monoclonal IgG1 antibodies, and it is not known how they would translate to a Fc-prolactin fusion.
**Use of mutations to decrease off-target Fc receptor binding**	IgG1 Fc domains bind to Fc receptors to activate immune effector functions. These are off-target interactions in a Fc-PRL fusion that can be abolished by mutating the Fc domain. These mutations are originally tested on monoclonal IgG1 antibodies, and how they translate to a Fc-PRL fusion is not known.
**Deglycosylation**	Both Fc and Prolactin are naturally N-link glycosylated. Deglycosylating both molecules makes it easier to manufacture

	because the resulting product is more homogenous. Deglycosylating prolactin should increase its activity because deglycosylated prolactin is inactive. Deglycosylating Fc decreases off-target binding to Fc receptors and increases susceptibility to GI proteases [10]. However, deglycosylating both molecules may decrease stability and increase aggregation.
**Use of mutations to Fc to abolish Protein A binding**	In Fc-Prolactin heterodimers with a single prolactin, the Fc only monomer can dimerize or be unpaired if overexpressed. These species are not ideal in commercially manufactured drugs. The Fc only monomer can be mutated to abolish Protein A binding, which prevents it from being purified with the correctly formed heterodimer [12,13]. This is dependent on titer, purification conditions, and the version of commercially available Protein A used in the purification process [12,13].

Table S5: Patterns emerging from Fc-prolactin fusions

Design Scheme	Pattern
**N-PRL-Fc-C fusions**	• Lower expression titers than N-Fc-PRL-C heterodimers and homodimers (Fig. S3A; compare Fc-PRL-# 18-29 v. Fc-PRL-# 1-17)<br>• Dirtier purification than N-Fc-PRL-C heterodimers (compare Fig. S2E v. Fig. S2C)<br>• More prone to aggregations than N-Fc-PRL-C heterodimers (compare Fig. S2F v. Fig. S2D)<br>• Similar aggregation to homodimers (compare Fig. S2F v. Fig. S2B)<br>• Lower potency (Log (EC_{50})) than N-Fc-PRL-C heterodimers and homodimers (Table S6; compare Fc-PRL-# 18-29 v Fc-PRL-# 1-17)
**N-Fc-PRL-C fusions**	• Higher expression titers than N-PRL-Fc-C heterodimers (Fig. S3A; compare Fc-PRL-# 8-17 v. Fc-PRL-# 18-29)<br>• Purer purification than N-PRL-Fc-C heterodimers (compare Fig. S2C v. Fig. S2E)<br>• Less prone to aggregations than N-Fc-PRL-C homodimers and N-PRL-Fc-C heterodimers (compare Fig. S2D v. Fig. S2B and F)<br>• More potent (Log (EC_{50})) than N-PRL-Fc-C heterodimers (Table S6; compare Fc-PRL-# 8-17 v Fc-PRL-# 18-29)<br>• Slightly less efficacy (% WT hPRL Emax) than N-Fc-PRL-C homodimers and N-PRL-Fc-C heterodimers (Table S6: compare Fc-PRL-# 8-17 v. Fc-PRL-# 1-8 & 18-29)
**Homodimer fusions**	• Higher expression titers than heterodimers (compare Fig. S3A; Fc-PRL-# 1-7 v. Fc-PRL-# 8-29)<br>• More prone to aggregation than N-Fc-PRL-C heterodimers (compare Fig. S2B v. Fig S2D).<br>• Similar purity to N-Fc-PRL-C heterodimers (compare Fig. S2A v. Fig S2C).<br>• Purer purification than N-PRL-Fc-C heterodimers (compare Fig. S2A v. Fig S2E).<br>• More potent (Log (EC_{50})) and efficacious (% WT hPRL Emax) than heterodimers (Table S6; compare Fc-PRL-# 1-7 v Fc-PRL-# 8-29)
**Heterodimer fusions**	• Slightly lower expression titers than homodimers (compare Fig. S3A; Fc-PRL-# 8-29 v. Fc-PRL-# 1-7)<br>• Less potent (Log (EC_{50})) and efficacious (% WT hPRL Emax) than homodimers (Table S6; compare Fc-PRL-# 8-29 v Fc-PRL-# 1-7)<br>• Fc Knobs-in-hole has lower titers than Fc A/ Fc B heterodimers (Fig. S3A; compare Fc-PRL-# 8-12, 18-24 v. Fc-PRL-# 13-17, 25-29)
**GGsGG Linker**	• Decreases (Log (EC_{50})) and efficacious (% WT hPRL Emax) on homodimers (Table S6; compare Fc-PRL-4 v. Fc-PRL-# 1-3, 5-7)<br>• Increases efficacy (% WT hPRL Emax) on heterodimers (Table S7; compare Fc-PRL-8 v Fc-PRL-# 9-29)
**half-life extension Fc variants**	• YTE mutations enhance pH-dependent FcRn binding *in vitro* (Fig S7 A & B; compare Fc-PRL-7, 17, and 29 v. Fc-PRL-1, 3, and 13).<br>• YTE mutations have lower serum half-life than Fc without (Fig S12; compare Fc-PRL-3 v. Fc-PRL-7 and Fc-PRL-13 v. Fc-PRL-17).<br>• All Fc-PRL variants bind to human FcRn at pH 7.4 at high receptor concentrations like isotype controls.
**Mutations to decrease off-target Fc receptor binding**	

**Deglycosylation**	• Deglycosylating PRL slightly decreases potency (Log (EC_{50})) and efficacy (% WT hPRL Emax) compared to WT (Table S6; compare PRL N59D v. PRL WT)<br>• Deglycosylating PRL increases potency (Log (EC_{50})) in homodimers (Table S6; compare Fc-PRL-3 v. Fc-PRL-2)<br>• Deglycosylating PRL slightly decreases over GI protease degradation (Fig. S10, d-f), but deglycosylated PRL has increased internal fragmentation compared to PRL WT (Fig. S9).<br>• Deglycosylating Fc decreases Fc receptor binding (Fig. S8; compare Fc-PRL-1 v other Fc-PRL variants).<br>• Glycosylated Fc has higher Fc receptor binding than IgG isotype (Fig. S8)<br>• Deglycosylating Fc increases the rate of digestion by GI proteases (Fig. S10A-B, compare Fc-PRL-1 v Fc-PRL-# 3, 7, 13, and 17).<br>• Deglycosylating Fc increases internal fragmentation (not just hinge cleavage) compared to glycosylated Fc (Fig. S9; compare Fc-PRL-1 v. Fc-PRL-# 3, 7, 13, 17)<br>• Deglycosylating Fc does not increase susceptibility to serum proteases (Fig. S11; compare Fc-PRL-1 v. Fc-PRL-13)<br>• Deglycosylated PRL fused to Fc has no identified serum proteolysis (Fig. S11).
**Mutations to Fc to abolish Protein A binding**	• RF mutations to abolish protein A binding decreases Fc only monomer after protein A purification most notably in N-PRL-Fc-C fusions expressed in mammalian cells (Fig S2F; compare Fc-PRL-25 v. Fc-PRL 26-29).<br>• RF mutations do not affect human FcRn binding (Fig. S7A-B)<br>• RF mutations might abolish mouse FcRn binding (Fig. S7C; compare Fc-PRL-1and 7 v. Fc-PRL-13).<br>• RF mutations don't work as well at high titers with Protein A (Fig. S4). They are expected to work better with MabSelect SuRe[43].

Table S6. Bioactivity of Fc-Prolactin variants via human PRLR.

Table S6: Bioactivity of Fc-Prolactin variants via human PRLR signaling

Protein	N	Log (EC50)	% WT hPRL Emax
**PRL WT +**	5	-9.8	100%
**PRL WT ***	16	-9.7	100%
**PRL N59D +**	3	-8.9	95%
**Fc-PRL-1***	3	-10.2	101%
**Fc-PRL-2 +**	3	-8.0	127%
**Fc-PRL-3 ***	3	-10.1	104%
**Fc-PRL-4 +**	3	-7.9	49%
**Fc-PRL-5 ***	3	-9.7	103%
**Fc-PRL-6 ***	3	-10.0	75%
**Fc-PRL-7 ***	3	-10.3	107%
**Fc-PRL-8 +**	3	-9.4	47%
**Fc-PRL-9 ***	3	-9.9	113%
**Fc-PRL-10***	3	-9.5	103%
**Fc-PRL-11 ***	3	-9.7	71%
**Fc-PRL-12 ***	3	-9.8	95%
**Fc-PRL-13 ***	3	-9.9	98%
**Fc-PRL-14 ***	3	-9.4	96%
**Fc-PRL-15 ***	3	-9.9	91%
**Fc-PRL-16***	2	-9.7	58%
**Fc-PRL-17 ***	3	-9.8	120%
**Fc-PRL-18 +**	3	-8.8	75%
**Fc-PRL-19 ***	1	-8.8	131%
**Fc-PRL-20 +**	3	-8.5	81%
**Fc-PRL-21 +**	3	-8.4	103%
**Fc-PRL-22 ***	2	-8.3	126%
**Fc-PRL-23 ***	3	-9.1	73%
**Fc-PRL-24 ***	3	-6.9	109%
**Fc-PRL-25 ***	3	-8.8	100%
**Fc-PRL-26 ***	3	-8.5	102%
**Fc-PRL-27 ***	3	-9.1	94%
**Fc-PRL-28 ***	3	-9.2	70%
**Fc-PRL-29 ***	3	-9.2	118%

* Readout by MTS reagent
+ Readout by WST-1 reagent

Table S7. Bioactivity of Fc-Prolactin variants via mouse PRLR signaling.

Table S7: Bioactivity of Fc-Prolactin variants via mouse PRLR signaling

Protein	N	Log (EC50)	% WT mPRL Emax
**mPRL WT**	6	-9.8	100%
**hPRL WT**	3	-10.3	84%
**Fc-PRL-1**	3	-10.3	84%
**Fc-PRL-3**	3	-10.5	106%
**Fc-PRL-5**	3	-9.8	100%
**Fc-PRL-7**	3	-10.4	112%
**Fc-PRL-9**	3	-10.4	109%
**Fc-PRL-10**	3	-9.7	90%
**Fc-PRL-12**	3	-10.0	125%
**Fc-PRL-13**	3	-10.3	107%
**Fc-PRL-14**	3	-9.6	88%
**Fc-PRL-17**	3	-10.2	132%
**Fc-PRL-24**	3	-7.3	107%
**Fc-PRL-25**	3	-9.2	115%
**Fc-PRL-26**	3	-8.4	97%
**Fc-PRL-29**	3	-9.5	112%

Table S8. Bonferroni-adjusted p-values for multiple comparison via Two-way ANOVA.

Table S8: Bonferroni-adjusted p-values for multiple comparison via Two-way ANOVA

Day	Pup weight compared to Vehicle + PBS control	
	**BR + PBS**	**BR + 5mg/kg Fc-PRL-13 (single dose)**
**7**	0.0305 (*)	0.0726 (ns)
**8**	0.0507 (ns)	0.2240 (ns)
**9**	<0.0001 (****)	0.0009 (***)
**10**	<0.0001 (****)	0.0779 (ns)
**11**	<0.0001 (****)	>.9999 (ns)
**12**	<0.0001 (****)	0.0285 (*)
**13**	<0.0001 (****)	<0.0001 (****)
**14**	<0.0001 (****)	<0.0001 (****)
**15**	<0.0001 (****)	<0.0001 (****)
**16**	<0.0001 (****)	<0.0001 (****)
**17**	<0.0001 (****)	<0.0001 (****)
**18**	--	0.0006 (***)
**19**	--	0.0356 (*)
**20**	--	0.9615 (ns)
**21**	--	0.2499 (ns)

Table S9. Bonferroni-adjusted p-values for multiple comparison via Two-way ANOVA.

Table S9: Bonferroni-adjusted p-values for multiple comparison via Two-way ANOVA		
	Pup weight compared to PBS control	
**Day**	**I.V. 5mg/kg Fc-PRL-13 (single dose)**	**S.C. 5mg/kg Fc-PRL-13 (single dose)**
**7**	0.2060 (ns)	0.1398 (ns)
**8**	0.0507 (ns)	0.1146 (ns)
**9**	0.0096 (**)	0.0575 (ns)
**10**	0.1632 (ns)	0.1932 (ns)
**11**	0.5620 (ns)	0.2990 (ns)
**12**	0.4491 (ns)	0.5932 (ns)
**13**	0.7302 (ns)	>0.9999 (ns)
**14**	>0.9999 (ns)	>0.9999 (ns)
**15**	>0.9999 (ns)	>0.9999 (ns)
**16**	>0.9999 (ns)	>0.9999 (ns)
**17**	>0.9999 (ns)	>0.9999 (ns)
**18**	>0.9999 (ns)	>0.9999 (ns)
**19**	0.5366 (ns)	>0.9999 (ns)
**20**	0.7386 (ns)	0.7695 (ns)
**21**	>0.9999 (ns)	0.8015 (ns)

Table S10. Bonferroni-adjusted p-values for multiple comparison via Two-way ANOVA.

Table S10: Bonferroni-adjusted p-values for multiple comparison via Two-way ANOVA

Day	Pup weight compared to Vehicle + PBS control				
	**BR + PBS**	**BR + 0.05mg/kg Fc-PRL-13 (repeat dose)**	**BR + 0.5mg/kg Fc-PRL-13 (repeat dose)**	**BR + 5mg/kg Fc-PRL-13 (repeat dose)**	**BR + 5mg/kg PRL N59D (repeat dose)**
**7**	0.0508 (ns)	0.1751 (ns)	>0.9999 (ns)	0.3461 (ns)	>0.9999 (ns)
**8**	0.0845 (ns)	0.9613 (ns)	0.1071 (ns)	>0.9999 (ns)	0.1998 (ns)
**9**	<0.0001 (****)	0.0016 (**)	0.8271 (ns)	>0.9999 (ns)	0.0010 (**)
**10**	<0.0001 (****)	<0.0001 (****)	0.0632 (ns)	>0.9999 (ns)	<0.0001 (****)
**11**	<0.0001 (****)	<0.0001 (****)	0.0052 (**)	0.1509 (ns)	<0.0001 (****)
**12**	<0.0001 (****)	<0.0001 (****)	<0.0001 (****)	0.0013 (**)	<0.0001 (****)
**13**	<0.0001 (****)	<0.0001 (****)	<0.0001 (****)	0.0005 (***)	<0.0001 (****)
**14**	<0.0001 (****)	<0.0001 (****)	<0.0001 (****)	<0.0001 (****)	<0.0001 (****)
**15**	<0.0001 (****)	0.0048 (**)	<0.0001 (****)	<0.0001 (****)	<0.0001 (****)
**16**	<0.0001 (****)	<0.0001 (****)	<0.0001 (****)	<0.0001 (****)	<0.0001 (****)
**17**	<0.0001 (****)	0.0048 (**)	<0.0001 (****)	<0.0001 (****)	<0.0001 (****)
**18**	--	<0.0001 (****)	<0.0001 (****)	<0.0001 (****)	0.0145 (*)
**19**	--	--	<0.0001 (****)	0.0638 (ns)	0.0423 (*)
**20**	--	--	0.0002 (***)	0.1586 (ns)	0.1831 ns)
**21**	--	--	<0.0001 (****)	0.1067 (ns)	0.0823 (ns)

Table S11. Bonferroni-adjusted p-values for multiple comparison via Two-way ANOVA

Table S11: Bonferroni-adjusted p-values for multiple comparison via Two-way ANOVA

Day	Pup weight compared to BR + 5mg/kg PRL N59D (repeat dose)				
	**BR + PBS**	**BR + 0.05mg/kg Fc-PRL-13 (repeat dose)**	**BR + 0.5mg/kg Fc-PRL-13 (repeat dose)**	**BR + 5mg/kg Fc-PRL-13 (repeat dose)**	**Vehicle + PBS**
**7**	0.334 (ns)	0.7492 (ns)	>0.9999 (ns)	>0.9999 (ns)	>0.9999 (ns)
**8**	>0.9999 (ns)	>0.9999 (ns)	>0.9999 (ns)	0.1002 (ns)	0.2398 (ns)
**9**	0.5966 (ns)	>0.9999 (ns)	>0.9999 (ns)	0.0522 (ns)	0.0013 (*)
**10**	0.1164 (ns)	>0.9999 (ns)	0.5702 (ns)	0.1169 (ns)	0.0013 (**)
**11**	0.0897 (ns)	0.7195 (ns)	0.5091 (ns)	0.0192 (*)	<0.0001 (****)
**12**	>0.9999 (ns)	0.7940 (ns)	0.0361 (*)	0.0520 (ns)	<0.0001 (****)
**13**	>0.9999 (ns)	0.5652 (ns)	0.0015 (**)	<0.0001 (****)	<0.0001 (****)
**14**	0.8381 (ns)	0.9940 (ns)	0.1598 (ns)	0.0609 (ns)	<0.0001 (****)
**15**	>0.9999 (ns)	>0.9999 (ns)	>0.9999 (ns)	0.0387 (*)	<0.0001 (****)
**16**	>0.9999 (ns)	0.2185 (ns)	0.8476 (ns)	0.0572 (ns)	<0.0001 (****)
**17**	0.1588 (ns)	>0.9999 (ns)	0.3283 (ns)	0.0054 (**)	<0.0001 (****)
**18**	--	--	>0.9999 (ns)	>0.9999 (ns)	0.0181 (*)
**19**	--	--	0.6062 (ns)	0.6257 (ns)	0.0564 (ns)
**20**	--	--	>0.9999 (ns)	>0.9999 (ns)	0.2441 ns)
**21**	--	--	>0.9999 (ns)	0.8556 (ns)	0.1098 (ns)

Table S12. Bonferroni-adjusted p-values for multiple unpaired T-tests.

Table S12: Bonferroni-adjusted p-values for multiple unpaired T-tests

Day	5mg/kg Fc-PRL-13 (repeat dose) v. PBS control
7	>0.999999 (ns)
8	>0.999999 (ns)
9	>0.999999 (ns)
10	>0.999999 (ns)
11	>0.999999 (ns)
12	>0.999999 (ns)
13	>0.999999 (ns)
14	>0.999999 (ns)
15	>0.999999 (ns)
16	>0.999999 (ns)
17	>0.999999 (ns)
18	>0.999999 (ns)
19	0.584205 (ns)
20	>0.999999 (ns)
21	>0.999999 (ns)

SI References

1. Ridgway, J.B., Presta, L.G., and Carter, P. (1996). "Knobs-into-holes" engineering of antibody CH3 domains for heavy chain heterodimerization. Protein Eng. *9*, 617–621. https://doi.org/10.1093/protein/9.7.617.

2. Wei, H., Cai, H., Jin, Y., Wang, P., Zhang, Q., Lin, Y., Wang, W., Cheng, J., Zeng, N., Xu, T., et al. (2017). Structural basis of a novel heterodimeric Fc for bispecific antibody production. Oncotarget *8*, 51037–51049. https://doi.org/10.18632/oncotarget.17558.

3. Von Kreudenstein, T.S., Escobar-Carbrera, E., Lario, P.I., D'Angelo, I., Brault, K., Kelly, J.F., Durocher, Y., Baardsnes, J., Woods, R.J., Xie, M.H., et al. (2013). Improving biophysical properties of a bispecific antibody scaffold to aid developability. MAbs *5*, 646–654. https://doi.org/10.4161/mabs.25632.

4. Can, M., Guven, B., Atmaca, H., Acıkgoz, S., and Mungan, G. (2011). Clinical characterization of patients with macroprolactinemia and monomeric hyperprolactinemia. Kaohsiung J. Med. Sci. *27*, 173–176. https://doi.org/10.1016/j.kjms.2010.07.002.

5. Strohl, W.R. (2015). Fusion Proteins for Half-Life Extension of Biologics as a Strategy to Make Biobetters. BioDrugs *29*, 215–239. https://doi.org/10.1007/s40259-015-0133-6.

6. Lee, C.-H., Kang, T.H., Godon, O., Watanabe, M., Delidakis, G., Gillis, C.M., Sterlin, D., Hardy, D., Cogné, M., Macdonald, L.E., et al. (2019). An engineered human Fc domain that behaves like a pH-toggle switch for ultra-long circulation persistence. Nat. Commun. *10*, 5031. https://doi.org/10.1038/s41467-019-13108-2.

7. Bruhns, P., Iannascoli, B., England, P., Mancardi, D.A., Fernandez, N., Jorieux, S., and Daëron, M. (2009). Specificity and affinity of human Fcγ receptors and their polymorphic variants for human IgG subclasses. Blood *113*, 3716–3725. https://doi.org/10.1182/blood-2008-09-179754.

8. Arduin, E., Arora, S., Bamert, P.R., Kuiper, T., Popp, S., Geisse, S., Grau, R., Calzascia, T., Zenke, G., and Kovarik, J. (2015). Highly reduced binding to high and low affinity mouse Fc gamma receptors by L234A/L235A and N297A Fc mutations engineered into mouse IgG2a. Mol. Immunol. *63*, 456–463. https://doi.org/10.1016/j.molimm.2014.09.017.

9. Lo, M., Kim, H.S., Tong, R.K., Bainbridge, T.W., Vernes, J.-M., Zhang, Y., Lin, Y.L., Chung, S., Dennis, M.S., Zuchero, Y.J.Y., et al. (2017). Effector-attenuating Substitutions That Maintain Antibody Stability and Reduce Toxicity in Mice*. J. Biol. Chem. *292*, 3900–3908. https://doi.org/10.1074/jbc.M116.767749.

10. Tao, M.H., and Morrison, S.L. (1989). Studies of aglycosylated chimeric mouse-human IgG. Role of carbohydrate in the structure and effector functions mediated by the human IgG constant region. J. Immunol. *143*, 2595–2601.

11. Jasion, V.S., and Burnett, B.P. (2015). Survival and digestibility of orally-administered immunoglobulin preparations containing IgG through the gastrointestinal tract in humans. Nutr. J. *14*, 22. https://doi.org/10.1186/s12937-015-0010-7.

12. Zwolak, A., Leettola, C.N., Tam, S.H., Goulet, D.R., Derebe, M.G., Pardinas, J.R., Zheng, S., Decker, R., Emmell, E., and Chiu, M.L. (2017). Rapid Purification of Human Bispecific Antibodies via Selective Modulation of Protein A Binding. Sci. Rep. *7*, 15521. https://doi.org/10.1038/s41598-017-15748-0.

13. Tustian, A.D., Endicott, C., Adams, B., Mattila, J., and Bak, H. (2016). Development of purification processes for fully human bispecific antibodies based upon modification of protein A binding avidity. MAbs *8*, 828–838. https://doi.org/10.1080/19420862.2016.1160192.

14. Svensson, L.A., Bondensgaard, K., Nørskov-Lauritsen, L., Christensen, L., Becker, P., Andersen, M.D., Maltesen, M.J., Rand, K.D., and Breinholt, J. (2008). Crystal Structure of a Prolactin Receptor Antagonist Bound to the Extracellular Domain of the Prolactin Receptor*. J. Biol. Chem. *283*, 19085–19094. https://doi.org/10.1074/jbc.M801202200.

15. Saleem, M., Martin, H., and Coates, P. (2018). Prolactin Biology and Laboratory Measurement: An Update on Physiology and Current Analytical Issues. Clin. Biochem. Rev. *39*, 3–16.

16. Hashim, I.A., Aston, R., Butler, J., McGregor, A.M., Smith, C.R., and Norman, M. (1990). The proportion of glycosylated prolactin in serum is decreased in hyperprolactinemic states. J. Clin. Endocrinol. Metab. *71*, 111–115. https://doi.org/10.1210/jcem-71-1-111.

17. Jomain, J.-B., Tallet, E., Broutin, I., Hoos, S., van Agthoven, J., Ducruix, A., Kelly, P.A., Kragelund, B.B., England, P., and Goffin, V. (2007). Structural and thermodynamic bases for the design of pure prolactin receptor antagonists: X-ray structure of Del1-9-G129R-hPRL. J. Biol. Chem. *282*, 33118–33131. https://doi.org/10.1074/jbc.M704364200.

18. Prolactin: Structure, Function, and Regulation of Secretion | Physiological Reviews https://journals.physiology.org/doi/full/10.1152/physrev.2000.80.4.1523.

19. Zhu, J., Garrigues, L., Van den Toorn, H., Stahl, B., and Heck, A.J.R. (2019). Discovery and Quantification of Nonhuman Proteins in Human Milk. J. Proteome Res. *18*, 225–238. https://doi.org/10.1021/acs.jproteome.8b00550.

20. Beck, K.L., Weber, D., Phinney, B.S., Smilowitz, J.T., Hinde, K., Lönnerdal, B., Korf, I., and Lemay, D.G. (2015). Comparative Proteomics of Human and Macaque Milk Reveals Species-Specific Nutrition during Postnatal Development. J. Proteome Res. *14*, 2143–2157. https://doi.org/10.1021/pr501243m.

21. Silva, F.D., Oliveira, J.E., Freire, R.P., Suzuki, M.F., Soares, C.R., and Bartolini, P. (2019). Expression of glycosylated human prolactin in HEK293 cells and related N-glycan composition analysis. AMB Express *9*, 135. https://doi.org/10.1186/s13568-019-0856-8.

22. Morohoshi, K., Komatani, Y., and Harigaya, T. (2020). Estrogen induces phosphorylation of prolactin through p21-activated kinase 2 activation in the mouse pituitary gland. J. Reprod. Dev. *66*, 571–578. https://doi.org/10.1262/jrd.2020-080.

23. Bernard, V., Young, J., Chanson, P., and Binart, N. (2015). New insights in prolactin: pathological implications. Nat. Rev. Endocrinol. *11*, 265–275. https://doi.org/10.1038/nrendo.2015.36.

24. Kasum, M., Pavičić-Baldani, D., Stanić, P., Orešković, S., Sarić, J.-M., Blajić, J., and Juras, J. (2014). Importance of macroprolactinemia in hyperprolactinemia. Eur. J. Obstet. Gynecol. Reprod. Biol. *183*, 28–32. https://doi.org/10.1016/j.ejogrb.2014.10.013.

25. Bruhns, P., and Jönsson, F. (2015). Mouse and human FcR effector functions. Immunol. Rev. *268*, 25–51. https://doi.org/10.1111/imr.12350.

26. Moriwaki, M., and Welt, C.K. (2021). PRL Mutation Causing Alactogenesis: Insights Into Prolactin Structure and Function Relationships. J. Clin. Endocrinol. Metab. *106*, e3021–e3026. https://doi.org/10.1210/clinem/dgab201.

27. Lund, J., Pound, J.D., Jones, P.T., Duncan, A.R., Bentley, T., Goodall, M., Levine, B.A., Jefferis, R., and Winter, G. (1992). Multiple binding sites on the CH2 domain of IgG for mouse Fc gamma R11. Mol. Immunol. *29*, 53–59. https://doi.org/10.1016/0161-5890(92)90156-r.

28. Bailey, M.J., Duehr, J., Dulin, H., Broecker, F., Brown, J.A., Arumemi, F.O., Bermúdez González, M.C., Leyva-Grado, V.H., Evans, M.J., Simon, V., et al. (2018). Human antibodies targeting Zika virus NS1 provide protection against disease in a mouse model. Nat. Commun. *9*, 4560. https://doi.org/10.1038/s41467-018-07008-0.
